# From sequence to dynamics: the effects of transcription factor and polymerase concentration changes on activated and repressed promoters

**DOI:** 10.1186/1471-2199-10-92

**Published:** 2009-09-22

**Authors:** Abel González Pérez, Vladimir Espinosa Angarica, Julio Collado-Vides, Ana Tereza Ribeiro Vasconcelos

**Affiliations:** 1 Centro Nacional de Bioinformática, Industria y San José, Capitolio Nacional, CP 10200, Habana Vieja, Ciudad de la Habana, Cuba; 2 Departamento de Bioquímica y Biología Molecular y Celular, Facultad de Ciencias, Universidad de Zaragoza. Pedro Cerbuna 12, 50009 Zaragoza, Spain; 3 Instituto de Biocomputación y Física de Sistemas Complejos, Universidad de Zaragoza, Corona de Aragón 42 Edificio Cervantes, 50009 Zaragoza, Spain; 4 Programa de Genómica Computacional, Centro de Ciencias Genómicas, Universidad Nacional Autónoma de México, Av. Universidad s/n, Colonia Chamilpa 62210, Cuernavaca, Morelos, México; 5 Laboratório Nacional de Computação Científica, Av. Getulio Vargas 333, Quitandinha, CEP 25651-075, Petrópolis, Rio de Janeiro, Brasil

## Abstract

**Background:**

The fine tuning of two features of the bacterial regulatory machinery have been known to contribute to the diversity of gene expression within the same regulon: the sequence of Transcription Factor (TF) binding sites, and their location with respect to promoters. While variations of binding sequences modulate the strength of the interaction between the TF and its binding sites, the distance between binding sites and promoters alter the interaction between the TF and the RNA polymerase (RNAP).

**Results:**

In this paper we estimated the dissociation constants (*K*_*d*_) of several *E. coli *TFs in their interaction with variants of their binding sequences from the scores resulting from aligning them to Positional Weight Matrices. A correlation coefficient of 0.78 was obtained when pooling together sites for different TFs. The theoretically estimated *K*_*d *_values were then used, together with the dissociation constants of the RNAP-promoter interaction to analyze activated and repressed promoters. The strength of repressor sites -- i.e., the strength of the interaction between TFs and their binding sites -- is slightly higher than that of activated sites. We explored how different factors such as the variation of binding sequences, the occurrence of more than one binding site, or different RNAP concentrations may influence the promoters' response to the variations of TF concentrations. We found that the occurrence of several regulatory sites bound by the same TF close to a promoter -- if they are bound by the TF in an independent manner -- changes the effect of TF concentrations on promoter occupancy, with respect to individual sites. We also found that the occupancy of a promoter will never be more than half if the RNAP concentration-to-*K*_*p *_ratio is 1 and the promoter is subject to repression; or less than half if the promoter is subject to activation. If the ratio falls to 0.1, the upper limit of occupancy probability for repressed drops below 10%; a descent of the limits occurs also for activated promoters.

**Conclusion:**

The number of regulatory sites may thus act as a versatility-producing device, in addition to serving as a source of robustness of the transcription machinery. Furthermore, our results show that the effects of TF concentration fluctuations on promoter occupancy are constrained by RNAP concentrations.

## Background

Bacteria regulate gene expression in response to changing environmental conditions mainly through the modulation of transcription initiation. Regulatory proteins -- i.e., transcription factors, TFs -- change the probability with which the RNAP binds to promoter sequences, thus affecting the formation of a productive open complex and the success of messenger RNA synthesis [1-5]. In principle, two features of this regulatory machinery that encompasses the RNAP, transcription factors, and *cis*-acting sequences are subject to fine tuning: the sequence of TF binding sites and their location with respect to promoters [6-9]. The former influences the probability of transcription initiation by affecting the strength of the interaction established between the TFs and their binding sites [6,10], whereas changes in the latter alter the interaction between TFs and the RNAP and, consequently, the stability of the initial binary complex [1,4,8,9]. In a previous report, Buchler *et al*. [6] have compared the way this logic operates with a programmable computer.

Collections of experimentally verified TF binding sequences in model bacteria such as *E. coli *[11] have been used in the past two decades to assess the variability of regulatory sequences bound by the same TF [12-15]. These studies have taken a first step in the aim of explaining how this variability may serve the purpose of influencing different genes under the control of the same TF in the proportions required by the metabolic machinery for the cell to be able to adapt to a given environmental change. For instance, under a given stimulus, the transcription of some genes may be activated, while others are repressed by the same TF depending basically on where its binding sites are located with respect to the promoter [8,9]. The variability of "strength" of TF binding sequences -- estimated using various approaches -- has also been found in the transcription regulatory machinery of other bacteria [13,14,16], giving support to the hypothesis that it confers a clear evolvability advantage with respect to the alternative logic of placing the variability in binding strength solely in the TFs [6].

In this paper we explore how the variability of the "strength" of TF binding sites and promoter sequences influences the probability of RNAP-promoter interaction. (Throughout this paper, the words strength and strong are used in relation to binding sites or promoters to denote the intensity of the interaction between them and their respective TF or the RNAP.) The study was circumscribed to promoters affected by a single TF. First, we designed a way to assess the dissociation constants *K*_*d *_and *K*_*p *_-- i.e., those that govern the interaction between a TF and a binding site, and the RNAP and a promoter sequence, respectively. Our methodology consisted of interpolating the score of a regulatory or promoter sequence given by a Positional Weight Matrix (PWM) within a line that fits experimentally determined *K*_*d *_values to PWM scores calculated for the same sequences. Then, we used the thermodynamic approach and equations developed by Buchler *et al*. [6] from the original methodology by Shea and Ackers [17] to compute the probability of RNAP binding to the promoter as a function of TF and RNAP concentration. Unlike several recent works that have proposed meticulous kinetic models to explain detailed experimental observations of several phases of transcription initiation and elongation [18,19], we used the aforementioned equations to explore the variability of promoter occupancy of TUs within the same regulon.

We were able to find that arrays of closely located regulatory sites that are bound independently by the same TF -- i.e., those at which the mechanism of action of the TF may be fulfilled upon binding to an individual site -- change the probability of transcription initiation at promoters under their control with respect to a single-site scenario. In other words, the number of binding sites of a TF that are located within the regulatory region of a transcription unit (TU) have an impact on the level of occupancy of its promoter. The variability of regulatory sequences [6,10,20] and promoter-site distances have traditionally been recognized as mechanisms that produce this type of versatility on the effects of TFs. Nevertheless, this is to our best knowledge the first time that the occurrence of several regulatory sites near a single promoter has been recognized -- using theoretical modeling -- as a mechanism that contributes to the complexity and versatility of gene regulation. (See discussion in Ref. [7].) Finally, we also found that RNAP concentration constrains the impacts of TF concentration changes on promoter occupancy.

## Results

### Correlating *K*_*d *_and PWM scores

We obtained experimentally determined *K*_*d *_values for the interactions of several *E. coli *TFs with variations of their binding sequences from the ProNIT database [21]. At the same time, we extracted all TF binding sites from RegulonDB [11]. The set of binding sequence from ProNIT was filtered to minimize variability within the set (see Methods). The PWMs of the respective TFs -- obtained from RegulonDB [11] -- were employed to score the binding sequences in the filtered set. Sequences with identical PWM scores were grouped and their *K*_*d *_values were averaged, in order to produce single points for assessing the correlation between PWM scores and minus the logarithm of *K*_*d *_values. Table 1 summarizes the changes of set size through the steps outlined above, and a detailed description of the process may be found in the Methods section.

**Table 1:** Number of DNA sequences through the filtering process.

**TF**	**ProNIT**	**Filtered**	**Averaged**
**CRP**	81	60	24
**IHF**	1	1	1
**LacI**	17	3	1
**MetR**	8	0	0
**PurR**	18	8	1
**TrpR**	68	25	2
**Total**	193	97	29

**ProNIT**: Number of sequences originally extracted from ProNIT; **Filtered**: Number of sequences remaining after filtering by pH and Temperature; **Averaged**: Number of sequences after grouping by equal PWM score.

**Figure 1:**
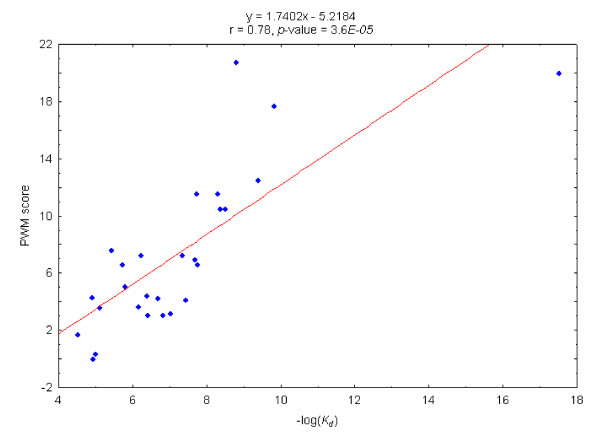
**Correlation between -*log(K_*d*_) *and PWM scores of DNA sequences downloaded from ProNIT**. The equation of the fitting line, the Pearson's correlation coefficient, and its associated *p-*value, resulting from 1000 randomizations of the original set are shown at the upper corner of the graph.

We used the Pearson's coefficient to measure the correlation between PWM scores and minus the logarithm of the *K*_*d *_of DNA binding sequences. In Figure 1, each point represents a DNA sequence, whose abscissa is the -*log(K*_*d*_*)*, and whose ordinate is the score resulting from aligning it to the PWM of the TF that specifically recognizes it. The Pearson's correlation coefficient of the two variables is 0.78, and its *p*-value estimated from 1000 randomizations of the data set, as described in the Methods section is 3.6E-05, indicating a fairly good agreement between the distribution of experimentally determined *K*_*d *_values and the scores calculated for the same DNA sequences.

This outcome highlights the usefulness of PWM scores as predictors of the *K*_*d *_(or *ΔG*) of the interaction between a given DNA sequence and the protein whose binding motif is represented by the PWM [10,20,22,23]. Furthermore, we estimated the *K*_*d *_of the interaction of a TF and a given DNA sequence by interpolating the score resulting from aligning the DNA sequence to the PWM within the fitting line. A previous work, circumscribed to *K*_*d *_values obtained by EMSA experiments that used FIS artificial binding sequences showed a similar trend and used the regression obtained between *K*_*d *_and sequence Information Content in an analogous manner [23]. Our results extend this view to a group of DNA sequences recognized by different TFs, suggesting that experimental data on protein-DNA interaction thermodynamics may be pooled together in order to obtain accurate theoretical estimates of the interaction parameters of new DNA sequences.

### The kinetic of transcription initiation

The *K*_*d *_values of TF binding sequences extracted from RegulonDB [11], with PWMs included in this database, were calculated interpolating the PWM scores within the fitting line (see Figure 1). We then interpolated the promoters' scores obtained as described in the Methods section into the fitting line in order to approximate the corresponding *K*_*p *_values -- i.e., dissociation constants of the interactions between promoter sequences and the RNAP. Both datasets (TF binding sites and promoters) were then crossed in order to form promoter-site pairs. These units were formed in a combinatorial manner in the cases of TUs with more than one TF binding site. We retained within the study only TUs for which both the promoter and the site had known scores and hence for which dissociation constants could be calculated for both.

**Figure 2:**
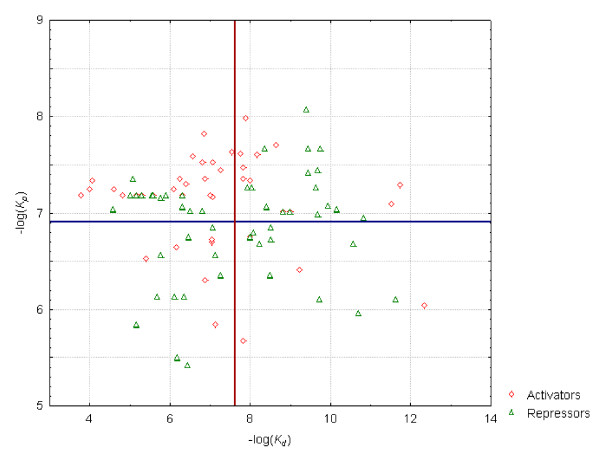
**Distribution of regulatory sites according to their *K*_*d *_values and the *K*_*p *_values of their corresponding promoter sequences**. The vertical and horizontal lines correspond to the mean -*log(K_*d*_)*, 7.63 and mean -*log(K*_*p*_*)*, 6.91 respectively.

The distribution of -*log(K*_*d*_*) *and -*log(K*_*p*_*) *values of the set of binding sequences and their corresponding promoter sequences appears in Table 2 (grouped by regulons) and is depicted graphically in Figure 2 (organized into activators and repressors). No clear trend may be discerned within the whole set when analyzing the relationship between the -*log(K*_*d*_*) *(and hence the strength) of a given regulatory site and the -*log(K*_*p*_*) *of its associated promoter. Nevertheless, while the -*log(K*_*d*_*) *values of activator sites are preferentially (65%) below the mean of the distribution, 57% of the repressor sites possess -*log(K*_*d*_*) *values higher than the mean of the distribution. With regard to the distribution of -*log(K*_*p*_*) *values, 77% of the promoters subject to activation have -*log(K*_*p*_*) *values above the mean; that fraction is reduced to 51% of the promoters associated with repressor sites. The mean -*log(K*_*p*_*)*of repressed promoters is hence lower than that of activated promoters: 6.77 *vs*. 7.12.

**Table 2:** Distribution of -*log(K_*d*_) *values of TFs binding sequences and -*log(K_*p*_) *values of their corresponding promoter sequences.

**TF**	**Units**	**-*log(K_*d*_)***						**-*log(K_*p*_)***					
		
		**Mean**	**SD**	**Max**	**Min**	**M-SD**	**M+SD**	**Mean**	**SD**	**Max**	**M-SD**	**M+SD**	**Min**
Ada	1	12.33	0	12.33	12.33	12.33	12.33	6.03	0	6.03	6.03	6.03	6.03
ArgR	2	7.36	1.47	8.39	6.33	5.90	8.83	7.06	0	7.06	7.06	7.06	7.06
CpxR	1	6.43	0	6.43	6.43	6.43	6.43	5.42	0	5.42	5.42	5.42	5.42
CRP	7	6.46	0.22	7.84	5.17	6.24	6.68	6.00	0.22	6.30	5.78	6.22	5.676
CsgD	1	8.23	0	8.23	8.23	8.23	8.23	6.68	0	6.68	6.68	6.68	6.68
CysB	2	11.62	0.16	11.73	11.51	11.46	11.78	7.19	0.14	7.29	7.05	7.33	7.0935
FIS	42	6.26	1.16	8.64	5.50	5.09	7.42	7.20	0.38	7.82	6.83	7.58	5.50
FNR	1	7.88	0	7.88	7.88	7.88	7.88	7.98	0	7.98	7.98	7.98	7.98
FruR	2	9.57	0.19	9.70	9.43	9.37	9.76	7.20	0.31	7.42	6.90	7.51	6.99
Fur	9	9.18	1.62	10.70	6.46	7.56	10.80	6.54	0.70	8.07	5.85	7.24	5.96
LexA	8	8.84	1.48	10.15	5.80	7.36	10.32	6.96	0.54	7.45	6.42	7.49	6.11
Lrp	1	5.07	0	5.07	5.07	5.07	5.07	7.35	0	7.35	7.35	7.35	7.35
MarA	1	8.08	0	8.08	8.08	8.08	8.08	6.80	0	6.80	6.80	6.80	6.80
NagC	4	8.91	0.11	9.00	8.82	8.80	9.02	7.01	0	7.01	7.01	7.01	7.01
PhoB	1	9.22	0	9.22	9.22	9.22	9.22	6.41	0	6.41	6.41	6.41	6.41
PhoP	2	4.58	0	4.58	4.58	4.58	4.58	7.03	0	7.03	7.03	7.03	7.03
PurR	3	9.22	1.18	10.58	8.54	8.04	10.40	6.71	0.03	6.73	6.69	6.74	6.68
TrpR	6	11.08	0.68	11.62	9.93	10.41	11.76	6.54	0.49	7.07	6.06	7.03	6.10
TyrR	11	7.96	1.17	9.76	5.78	6.80	9.13	6.84	0.57	7.67	6.27	7.41	6.34

M-SD: mean minus one standard deviation; M+SD: mean plus one standard deviation.

In general, the RNAP may bind to repressed promoters in the absence of the repressor TF [4], while its binding to activated promoters normally requires the establishment of protein-protein interactions with the activator TF. (This different behavior guarantees that, while genes controlled by activated promoters are expressed only in the presence of the activator TF, those regulated by repressed promoters may be expressed only if the repressor TF is absent.) Therefore, one should expect that the interaction between the RNAP and repressed promoters be as a rule stronger than its interaction with activated promoters. This reasoning is contradicted by the above described findings. However, it is important to bear in mind that we are working with an incomplete set of promoters which may not represent well the universe of simple promoters. Furthermore the *Kp *characterizes only the strength of the interaction between the RNAP and the promoter, whereas the efficiency of transcription initiation may be submitted to influences that affect different stages of the process. Promoters that bind RNAP weakly may be strong if the rest of the steps of transcription initiation are optimized [4].

In order to simulate the kinetics of transcription initiation -- i.e., the probability of RNAP-promoter binding -- we followed the formalism developed by Buchler *et al*. [6] from an original approach by Shea and Ackers [17]. This model computes the probability of RNAP-promoter interaction -- as an indicator of TU transcription initiation probability -- as the fraction of time that the RNAP is bound to the promoter;

(I)

where *Z*_*on *_and *Z*_*off *_represent the partition sum of the Boltzmann weights W over all states of TF binding for the promoter bound and not bound respectively. Since we worked only with simple promoters -- i.e., those for which the binding of the RNAP is affected by a single TF --, these quantities may be calculated by:

(II)

(III)

where [*Pol*] and [*TF*] are, respectively, the concentration of the RNAP and the TF; *K*_*d *_and *K*_*p *_are, respectively, the dissociation constants of the binding of the TF to its site and that of the RNAP to the promoter; and *ω *is a qualitative factor that represents the type of interaction established between the TF and the RNAP. In the case of repressors, *ω *equals 0, which represent the mutual exclusion of the TF and the RNAP from their respective binding loci. In the case of activators, the cooperative binding of the TF and the RNAP is represented by a ω value of 20. See Methods for details.

**Figure 3:**
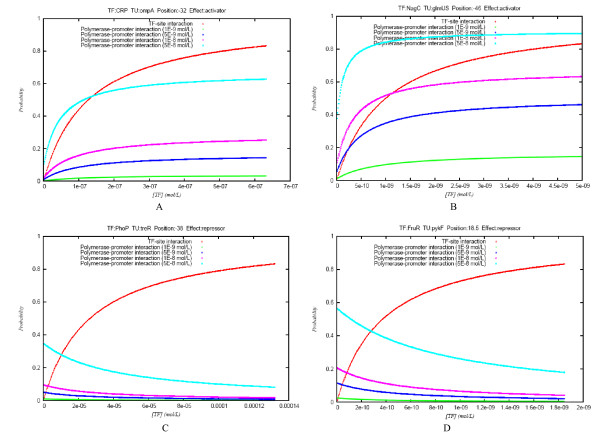
**Probability of TF-site interaction and transcription initiation (RNAP-promoter interaction) as a function of TF concentration at four simple promoters**. Panels A and B correspond to activator sites; C and D represent repressor sites.

The graph depicted in Figure 3 shows the probability of interaction between the TF and its binding site (red curve), calculated as  and the probability of RNAP-promoter binding at four different RNAP concentrations, from 1E-09 to 5E-08 (remaining curves). In order to facilitate comparisons, TF concentration values in all graphs range from 0 to approximately 4.5 times *K*_*d*_; therefore, the red curves of all graphs are identical. As may be readily inferred from the previous equation, for activator sites (panels A and B in Figure 3) the greater the TF concentration at constant RNAP concentration, the more likely for the RNAP to bind to the promoter. The increase of RNAP concentration, on the other hand, decreases the amount of TF necessary to attain the saturation of the promoter. Nonetheless, while at [*TF*] = *K*_*d *_the promoter of graph A is occupied by the RNAP roughly little above 50% of the time at the highest RNAP concentration, at the same TF and RNAP concentrations, the promoter of graph B remains occupied more than 80% of the time. This dissimilarity of behavior can only be explained by the difference in *K*_*p *_values between the two promoters. As a rule, for activator sites, the stronger the promoter, the lower the RNAP amount required to saturate it at the same TF concentration. The graphs of the kinetic behavior of all simple promoters can be found in Additional file 1. The results for repressor sites (Figures 3C and 3D) are the exact opposite. As TF concentration increases, the less likely the RNAP will be bound to the promoter. This decrease in promoter activity is less dramatic the higher the RNAP concentration or the lower the *K*_*p *_value.

**Figure 4:**
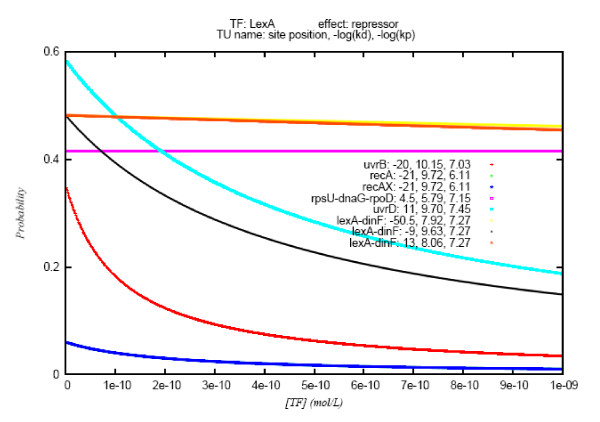
**Probability of RNAP-promoter binding *vs *TF concentration for several promoters repressed by LexA**. RNAP concentration: 5E-08 mol/L. Graphs' legend: Transcription Unit name (gene names concatenated by underscores): position of the regulatory site, -*log(Kd)*, -*log(Kp)*.

The simultaneous effects of the two dissociation constants affecting the same promoter may be further appreciated in Figure 4, which illustrates the kinetics of several promoters repressed by LexA within the same range of TF concentrations and at the same RNAP concentration. In the case of the promoter of the *lexA_dinF *TU, for instance, the binding of LexA to three different sites hinders the binding of the RNAP to the promoter. This allows the comparison of the kinetic behavior of three binding sites affecting the same promoter (and hence, with identical *K*_*p*_). The strongest site (located at -9 bps with kinetic curve in yellow) encounters LexA concentration values that range very close to its *K*_*d *_(2.3E-10); on the other hand, the *K*_*d *_values of the other two LexA sites are lower by roughly one and two orders of magnitude (dark blue, 8.7E-09) than the site located at +13 bps and (brown 1.2E-08) than the one at -50.5 bps, respectively. Therefore, at the range of LexA concentrations employed in the simulations, the -9 bps site causes a drop of over 30% of promoter occupancy, while the effect of the other two sites is almost unchanged along the range.

The comparison of the behavior of the *recA *and the *uvrD *promoters enables a similar analysis for sites with comparable *K*_*d *_values (2E-10 and 1.91E-10, respectively). Although the TF concentrations evaluated are of the same order of the *K*_*d *_of both regulatory sites, while the variation in promoter occupancy experienced by *recA *is below 10% along all the range of TF concentrations, *uvrD *experiences a drop from almost 60% to less than 20%. *K*_*p *_values that differ by almost one order of magnitude are the key to this variation. Whereas the RNAP concentration employed in the calculation (5E-08) is very similar to the *K*_*p *_of the *uvrD *promoter (3.55E-08), it is significantly lower than that of *recA *(7.76E-07), thus causing the noticeably lower occupancy of the latter promoter within the range of TF concentrations evaluated.

**Figure 5:**
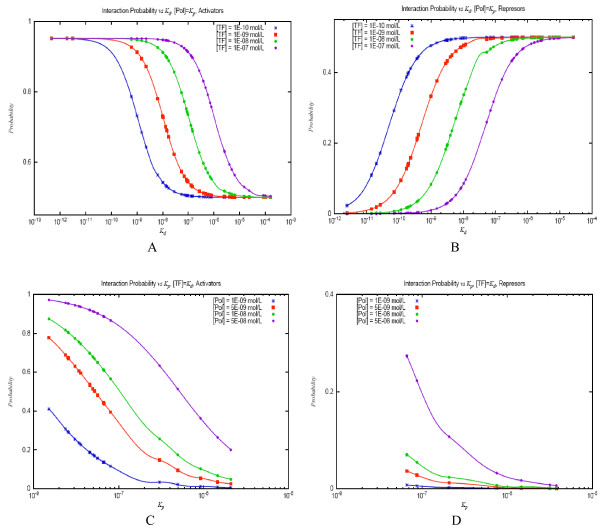
**Probability of RNAP-promoter interaction**. A, activator sites, at RNAP concentration equal to *K*_*p *_of promoters, at four TF concentrations; B, repressor sites at RNAP concentration equal to *K*_*p *_of promoters, at four TF concentrations; C, activator sites, at TF concentration equal to *K*_*d *_of sites, at four RNAP concentrations; D, repressor sites, at TF concentration equal to the *K*_*d *_of sites, at four RNAP concentrations.

Finally, we obtained a global representation of the reactions of all simple promoters within the study to wide variations of TF (and alternatively RNAP) concentrations. In order to carry out this analysis, we separated the promoters associated with repressor sites from those associated with activator sites. To assess the response to varying TF concentrations, the RNAP concentrations in the simulations were kept equal to the *K*_*p*_of promoters; therefore, we compared the influence of regulatory sites' strength on equivalent conditions for all promoters. In the alternative analysis, we maintained TF concentrations equal to the *K*_*d *_of regulatory sites, hence assessing how promoters' strength affects their occupancy when their associated regulatory sites are comparably (half) occupied. The results of both analyses are presented in Figure 5.

Every point in panel A of Figure 5 represents a promoter-site unit, its abscissa being the *K*_*d *_of the TF binding site and its ordinate, the probability of RNAP-promoter binding. The color of the point corresponds to the TF concentration at which the probability was calculated, according to the legend at the left side of the pane. Combining equations I, II, and III, applying the restriction [*Pol*] = *K*_*p*_, and substituting *ω *by 20, we find that the probability of RNAP-promoter binding for activators may be calculated by:



The graph in panel A shows that if differences in promoters' strength are disregarded (with [*Pol*] = *K*_*p*_), at the same TF concentrations, the dependency of the probability of promoter occupancy on regulatory site strength follows roughly a sigmoid curve. This may be obtained from the previous equation: if the TF concentration is kept at negligible values with respect to *K*_*d*_, the promoter is half occupied. On the other hand, at high TF concentration values relative to *K*_*d*_, activated promoters tend to be occupied almost permanently. These two boundaries of the equation determine the sigmoid shape of the probability (semi-*log*) graph, with its linear portion populated by regulatory sites with *K*_*d *_values approximately within two orders of magnitude immediately above the TF concentration. Varying the TF concentration causes a shift of the probability distribution: as the former increases, the latter is displaced to the right, with more promoters close to saturation and fewer promoters in the half occupied state.

The probability of RNAP-promoter interaction in the case of repressor sites applying the aforementioned conditions (with *ω *= 0) and restrictions may be computed by the equation:



whose results on the set of promoter-site units within our study are represented graphically in pane B of Figure 5. In this case, the upper and lower limits of the probability values are 0.5 and 0, respectively, and the analysis of the graph shows that, as expected, promoters associated with stronger sites tend to have lower probability of occupancy and the shift imposed on the graph by varying TF concentrations is the opposite of the one observed for activators. In this case, the linear portion of the sigmoid is composed of promoter-site units with *K*_*d *_values approximately within the two orders of magnitude at both sites of TF concentration.

Panels C and D of Figure 5 correspond to the assessment of the probability of RNAP-promoter binding *vs *the *K*_*p *_of simple promoters, calculated at TF concentrations equal to the *K*_*d *_of their associated regulatory sites. In this analysis, all regulatory sites are half occupied; therefore, by combining Equations I, II, and III, and transforming them accordingly ([*TF*] = *K*_*d*_), the probability of RNAP-promoter binding for activator sites (*ω *= 20) may be expressed as:



The graphs that correspond to promoters with activator sites (panel C) exhibit some similarities with their counterparts from the previous analysis (panel A). Their shapes tend to follow a sigmoid as the RNAP concentration increases. The lowest RNAP concentration used in this analysis is one order of magnitude lower than the *K*_*p *_of the strongest promoters. However, these graphs present no discernible (and invariant with RNAP concentration) limits: instead, they occupy the entire scale of probability values, and their lowest and highest values are determined by RNAP concentration. This means that if an activator site is half occupied, the occupancy of the promoter it regulates may be close to 100% given that RNAP concentration is within the same order of magnitude as its *K*_*p*_. On the other hand, if the RNAP concentration is between two and three orders of magnitude lower than the promoter *K*_*p*_, it will be unoccupied almost 100% of the time, even if the activator site that regulates it is half occupied.

The results for promoters associated with repressor sites are somewhat different. The probability of the RNAP binding them, given all previously mentioned conditions, is described by:



In this case, the shape of the graph is closer to a negative exponential whose fall becomes steeper as the RNAP concentration increases. Obtaining a sigmoid curve in this case requires RNAP concentrations on the order of 10^-6^, much higher than that observed physiologically [2]. A RNAP concentration higher by almost one order of magnitude than the *K*_*p *_of a promoter increases its probability of occupancy to a value below 80%. This number decreases rapidly with promoters' strength: for a value of RNAP concentration lower, by exactly one order of magnitude than promoter *K*_*p*_, its occupancy is around 25%.

## Discussion

The *log*-likelihood function employed to compute the information content of a group of known TF binding sequences is associated with the free energy of interaction between the TF and the DNA sequences. Specifically, this information is an estimate of the average specific binding energy for this set of known binding sites [10,20,22]. Several studies have used this property either to compute an experimental free energy matrix for a TF [24] or to correlate the information content of individual binding sites of a TF -- calculated from a previously constructed PWM -- to their experimentally estimated *K*_*d *_[23]. In addition, some other papers have used several structure-based theoretical approaches to calculate interaction energies between DNA sequences and TFs and compared them with experimentally determined values, in some cases with the aim of discovering new TF binding sites [25-27].

In this work, we combined a set of TF-DNA sequence dissociation constants calculated by different experimental strategies for a group of six TFs (under similar experimental conditions) to assess their correlation with the information content obtained for those same sequences when they were scored against PWMs of the TFs. Not surprisingly, the correlation between these two variables was weaker (r = 0.78 against r = 0.85) than the one found by Shultzaberger *et al*. [23] for sequences of a single TF (FIS), whose *K*_*d *_were determined by a unique experimental approach. This weaker correlation is probably a consequence of the difference in quality of the PWMs of different TFs and the fact that *K*_*d *_values in ProNIT are generated by different experimental procedures. These two factors produce the outliers in the graph of Figure 1. Since the accuracy of the calculation of *K*_*d *_and *K*_*p *_depends on the quality of this fitting line, it would be important to consider, as part of the proposed extension of this work, to improve the starting data -- including refining the PWMs and experimental data sources -- of promoters and TF binding sites.

Nevertheless, we decided that at this stage the obtained coherence between theory and experiment was sufficient towards the main goal of our work: to produce a primary estimation of the *K*_*d *_values of real *E. coli *regulatory sites and use them to study the kinetic response of their associated promoters to variations in site strength. In other words, we intended to explore first, the dynamical behavior of activated and repressed promoters as TF and RNAP concentrations change, and second, how transcription initiation at various promoters regulated by the same TF may respond differently to changes of its concentration through the influence of different factors, such as the variation of its binding sequences, the occurrence of more than one binding site, or RNAP concentration.

The analysis of the kinetic of RNAP-promoter binding, exemplified through the behavior of LexA in Figure 4, revealed several interesting insights. One first aspect that becomes apparent from the analysis of Table 2 is that the standard deviation of -*log(K*_*d*_*) *does not surpass 20% of the mean within any regulon. This implies that over 60% of the regulatory sites possess *K*_*d *_values that fall roughly within the three orders of magnitude centered at the mean of the distribution. This is approximately the range within which TF concentration value fluctuations may produce changes of promoter occupancy, at RNAP concentration values that are lower by 1-3 orders of magnitude than the promoter *K*_*p*_, as is the case in physiological conditions [2]. Figure 5A illustrates that promoter-sites are responsive to changes in TF concentration values within the two orders of magnitude immediately below the *K*_*d*_, when the promoter is half occupied. Moreover, most outliers of regulons' *K*_*d *_distributions correspond to sites associated with promoters with other sites whose *K*_*d *_values are closer to the distribution mean.

The previous discussion implies that the versatility of TFs, understood as the ability to produce different outcomes at the level of (simple) promoter occupancy only by virtue of modulations of its binding sequences, has precise limitations. In other words, only a limited number of base pair modifications in the site will produce binding sequences that are still responsive to physiological TF concentrations. Another mechanism that resulted in the increase of versatility in the course of evolution is the modulation of protein-protein interactions through changes in promoter-site distances [7-9,14].

The comparison of the strength of several binding sites upstream various LexA regulated promoters allowed us to explore the effect of the existence of several binding sites that may be independently bound by a TF. Although some TFs exert their action on promoters through their simultaneous binding to several sites leading to the formation of tetrameric molecules [28,29], this is not the case for LexA, which *in vivo *binds to each site as a dimeric molecule [30]. Our findings suggest that placing more than one LexA binding site in the vicinity of a promoter may be a mechanism of modulation of transcription initiation rate at that promoter. This "redundant" design is illustrated in Figure 4. For instance, the regulatory region of the *lexA_dinF *TU has three LexA binding sites with *K*_*d *_values of 1.2E-08, 8.65E-09, and 2.32E-10. One may assume that for sufficiently distant sites a LexA molecule bound to one site does not hinder occupation of another site, as may be the case for the first site (at -50.5 bp) with respect to the other two (at +13 bp and -9 bp, respectively). On the other hand, close sites may interact with each other in a way that only one of them may be occupied at a given time, such as sites two and three in the previous example. The actual outcome, in terms of promoter occupancy, in both scenarios would be different than the one calculated for each separate site, shown in Figure 4A. The probabilistic nature of the interaction between the TF and the DNA would determine that the chance that at least one of the sites be occupied is higher than the likelihood of occupancy of any of them separately.

**Figure 6:**
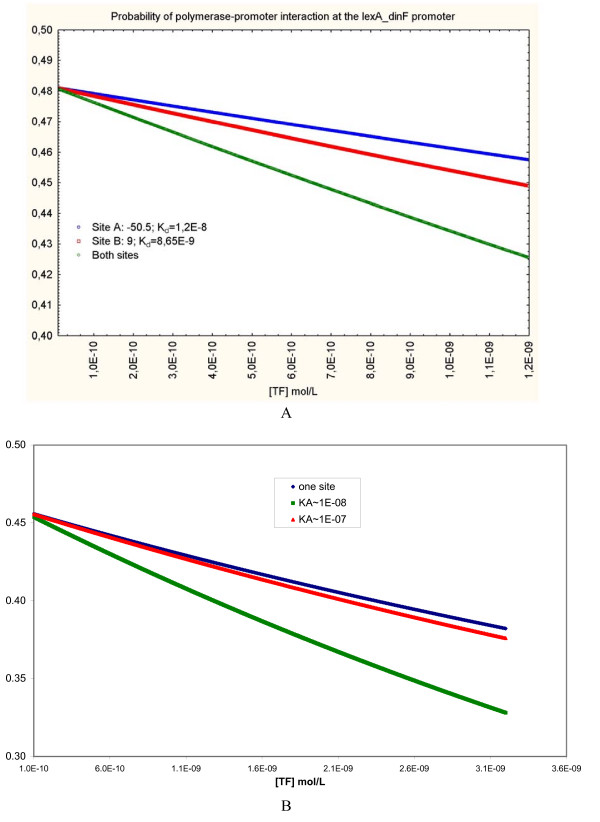
**Probabilities of *lexA_dinF *promoter occupancy calculated only on the basis of two of the LexA binding sites located within the regulatory region of the TU**. Panel A: The blue dots represent probability of promoter occupancy calculated considering only occupation of the -50.5 LexA binding site. The red dots represent probability of promoter occupancy calculated considering only occupation of the -9 site. The green dots represent probabilities calculated relying on the possibility of occupancy of either site by LexA. (RNAP concentration: 1E-08). Panel B: The blue and green dots represent exactly the same calculations as in panel A; the red dots represent the probability of *lexA_dinF *promoter occupancy calculated assuming that the -9 site mutates to produce a sequence whose binding to LexA is weaker by one order of magnitude than the wild type.

One way to estimate the probability of promoter occupancy considering that either one of two LexA binding sites may be occupied is by using the equation of the logical OR gate implemented by Buchler *et al*. [6]. Although they originally employed it to compute the probability of RNAP-promoter interaction in regulatory constructs at which a promoter is under the regulation of two TFs, it may be applied to the case of a promoter regulated by two sites bound by the same TF. We selected the -50.5 bp and the -9 bp sites to study how multiple independent sites affect promoter occupancy. Let the dissociation constants of their respective interaction with LexA be labeled *K*_*A *_and *K*_*B*_, then the probability of RNAP-promoter interaction, applying the OR gate logic, may be calculated as follows:



Figure 6A shows the results of substituting *K*_*A*_, *K*_*B*_, *K*_*P*_, and [*Pol*] by their values in this example, and calculating the probabilities of the *lexA_dinF *promoter occupancy considering a) only occupancy of site A by LexA, b) only occupancy of site B by LexA, and c) occupancy of either site by LexA. As expected, within the range of TF concentrations assayed the probability of promoter occupancy decays at a higher rate when both sites are considered than for each individual site. (Opposite results will be obtained for promoters under the control of more than one activator site). In sum, our results suggest that the location of multiple LexA binding sites in regulatory regions may be regarded not only as a source of robustness of the SOS system -- that increases its resistance to mutations affecting LexA binding sites -- but also as a device of gene expression fine tuning in response to changes of LexA concentration. This conclusion may be generalized to TFs that bind independently to several sites upstream a promoter: the occurrence of multiple binding sites of this nature may act, together with variations of TF binding sequences and promoter-site distances, as a modulator of the effects of TF binding upon promoter occupancy by the RNAP.

However, there is a limit to the effect of a weak site on the probability of polymerase-promoter interaction. A mutation that rendered site B weaker by only one order of magnitude would almost override its effect on *lexA_dinF *promoter occupancy, as presented in Figure 6B. The *K*_*d *_of this theoretically mutated site B corresponds to a PWM score of approximately 7.02, very close to the weaker LexA site (7.034, upstream uvrB) in our starting data set. This limit is probably a mechanism that prevents any sequence within regulatory regions from significantly affecting the effect of strong TF sites on promoter occupancy. In other words, while weak sites may indeed ploy a role in affecting the regulatory output of strong sites located in their vicinity, only true TF binding sites may do this; and the stronger the strong site, the stronger the weak site must be in order to have a significant effect on polymerase-promoter interaction.

Finally, we analyzed the general landscape of transcription initiation regulation through simple promoters by assessing the probability of promoter occupancy, first as the response of promoter-site units when the former is half occupied and second as their response to changes in RNAP concentration when regulatory sites are half occupied changing TF concentrations. Clearly, the first theoretical situation is far from the natural behavior of promoter-site units, which respond as a whole set to a single RNAP concentration. Nevertheless, some interesting extrapolations on the kinetic behavior of promoter-site units can be made from these theoretical results. First, it becomes apparent from panes A and B of Figure 5 that RNAP concentration imposes boundaries to promoter-site units' responsiveness to changes in TF concentrations. In this case, these limits are set to 0.5-1 for activators and 0-0.5 for repressors. In other words, if RNAP concentrations are about the *K*_*p *_of a promoter, and it is under the regulation of a repressor site, its occupancy will never be higher than 50% (or lower than 50% if its associated site is an activator), irrespective of the concentration of the TF that binds to the site. The variation of RNAP concentration changes these limits. For instance, if it falls to one-tenth of the *K*_*p*_, the upper limit of occupancy probability for promoters associated with repressor sites drops below 10%; a descent of the limits occurs also in the case of activator sites (Additional file 2).

In general terms, these results imply that RNAP concentrations impose restrictions on the effects that changes of TF concentrations may produce on promoter occupancy. They constrain the growth of promoter occupancy that may result from increasing -- or activating -- an activator TF, or from decreasing a repressor TF -- for instance, through the presence of an inducer.

### Testing model predictions

In order to indirectly test the validity of our model, we compared theoretical predictions made using our equations with microarray data from FNR-activated TUs in three experimental conditions (aerobiosis, presence of nitrate, and presence of nitrate). Briefly, we obtained microarray data for FNR-activated TUs in three experimental conditions from Constantinidou *et al*. [31] and used them to compute activated FNR concentration in those three situations. Using each TU iteratively as predictor in this manner, we calculated the theoretical Polymerase-promoter binding probability for all other promoter-site complexes under the same simulated condition with respect to the reference culture. Finally, we evaluated the degree (or trend) of activation of each TU in cells cultivated with nitrate (or nitrite) with respect to those cultivated under aerobiosis, both experimentally and theoretically, and computed the consistency between experimental and theoretical equivalent ratios. (The procedure is described in detail in Additional file 3 which also presents the results of calculations and comparisons between experimental and theoretical trends.)

To analyze the results, we considered three levels of consistency between theoretically predicted ratios and experimental ones. First, the ratio of theoretical to experimental quotient is between 0.5 and 2 -- i.e.: the disagreement between theoretical and experimental ratios is no more than two-fold --; second, the ratio is between 0.25 and 4; third, the ratio is between 0.1 and 10. We found that 65% of the predicted ratios are consistent with experimental ones according to the first; the disagreement of 81% of them with experimental ratios is no more than four-fold; and 93% of them are of the same order of magnitude than experimental ratios. Several points may be raised to explain why we fail obtaining a perfect consistency between theoretical and experimental trends. First, it is important to bear in mind the limitations of the model we employed, limited to representing the first step of transcription initiation, a complex processed whose dynamical behavior is influenced by a number of other stages. Second, the computations rely on thermodynamic constants approximated from a correlation, which was obtained employing fragmented experimental data. Finally, the noisy nature of microarray data [32] is another point to take into consideration. Taking all these factors into account, the levels of consistency found in this confrontation may be considered acceptable.

## Conclusion

A fairly good correlation between experimentally determined *K*_*d *_values and PWM scores of regulatory sites allowed us to approximate theoretical *K*_*d *_values of *E. coli *known regulatory sequences and *K*_*p *_values of their associated simple promoters. Using a formalism developed somewhere else [6], we explored how variations of TF concentrations impact the probability of RNAP-promoter interaction, and thus influence the process of transcription initiation, in order to understand how diverse promoters under the control of the same TF may produce different outcomes by virtue of different variables, such as the variations of their regulatory sequences, the location of several sequences bound independently by the same TF, or RNAP concentrations.

The variations of regulatory sequences bound by the same TF and changes of promoter-site distances have long been recognized as mechanisms that have resulted in increasing the versatility of gene expression outcomes within a regulon in the course of the evolutionary process. Nevertheless, we found that placing several regulatory sites bound by the same TF close to a promoter -- if they are bound by the TF in an independent manner -- may act as a third versatility-producing device, in addition to serving as a source of robustness of the transcription machinery. We also observed that RNAP concentrations impose well-defined constraints to the impact of fluctuations of TF concentrations on promoter occupancy. These results open the perspective of extending this study in three main areas: a) improving promoter and TF starting data in order to improve the correlation between PWM scores and *K*_*d*_; b) extending the model to promoters regulated by more than one TF (with the aim of studying the dynamics of the regulation of genes involved in closely related biochemical processes) and relaxing protein-protein interaction coefficients (ω) to more accurately reflect the wide repertoire of contacts between the TFs and the RNAP; and c) designing new strategies to confront theoretical predictions with microarray data.

## Methods

### Obtaining and processing data

Experimentally calculated thermodynamics constants (*K*_*d *_and *ΔG*) of the interaction between 6 *E. coli *TFs and variants of their DNA binding sequences (which totaled 193; see Table 1) were downloaded from the ProNIT database [21] in July 2008. (Most data collected in this database were obtained from experiments of gel shift, fluorescence, filter binding, calorimetry, among others.) From this original set we extracted the data that corresponded to experiments carried out at 25°C, and within a range of pH from 7.3 to 7.8, in order to reduce the sources of variation in the set. As a consequence, the number of sequences was reduced to 97.

At the same time, we obtained the PWMs representing *E. coli *TF binding sites (and all the information on regulatory sites) from RegulonDB, release 6.2 [11], and scored the binding site variants from ProNIT using the Patser program included within the Consensus package [33,34]. Certain DNA sequences bound by the same TF (that produced identical PWM scores) presented different *K*_*d *_values. This was the case not only for identical DNA sequences, where experimental variability or the employment of two or more different methods may have led to computing slightly different *K*_*d *_values, but also with very similar ones, which may be discriminated by the TF (thus producing different interaction *K*_*d *_values), but not by the PWM, which relies solely on the information of binding sequences positional conservation [20,32]. The *K*_*d *_values of these sequences with equal PWM score were averaged, thus resulting in a set of 29 sequences described by unique pairs of *K*_*d *_and PWM values.

The data on *E. coli *Sigma 70 promoters were downloaded from the Center of Genomics' Repository at [35]. Promoters' scores in this repository are calculated as a simple sum of the PWM scores of the -10 and -35 boxes. The information contained in this file regarding promoters' location was used to link the promoters to the corresponding TF binding sites. We used TUs -- or promoter-site units -- as the basic elements of our analysis in the subsequent parts of the study. Each TU was therefore represented as a pair of PWM scores: the score of the TF binding site (TUs regulated by more than one TF were eliminated from the set), and that of the promoter. Only TUs with a full pair of scores were maintained within the study. On the other hand, TUs with more than one binding site for the TF were multiplied as many times as necessary to include all of their binding sites. After all these processes, the set was composed of 105 TUs.

### Assessing the correlation between experimental Kd values and PWM scores

Let *K*_*di *_be the *K*_*d *_value of the i-*th *DNA binding site within the ProNIT filtered set; let score_*i *_be the score calculated for that same DNA sequence using the PWM of the corresponding TF. The mean of the *K*_*d *_and PWM scores distributions may be written as  and , respectively. Then, the equation to calculate the Pearson's correlation coefficient (*p*) is:



To assess the statistical significance of the *p *computed, we reshuffled the pairs of *K*_*d*_-PWM score values 1000 times and re-calculated the Pearson's correlation coefficient of each randomized set. We then computed the *Z*-score and the associated *p*-value of the Pearson's correlation coefficient that corresponded to the original set.

### Parameters of kinetic simulations

Whereas in the work by Buchler *et al*. (2003) the values of *q*_*p *_([*Pol*]/*K*_*p*_) and *K*_*d *_are set theoretically, here we estimated the values of *K*_*p *_and *K*_*d *_for each individual promoter and site, respectively from experimental data. Therefore, we only changed in each simulation the values of [*Pol*] and [*TF*]. The values of the RNAP concentration have been calculated within the nanomolar range in the *E. coli *cytoplasm (DeHaseth *et al*., 1998). Therefore, we employed [*Pol*] values between 10^-10 ^and 10^-8 ^in all the simulations. The concentration of TFs was taken, most of the times, very close to the *K*_*d *_of the site, in order to explore the behavior of promoter-site units within the responsive [*TF*] range. Alternatively, in some simulations, [*TF*] was varied across wider ranges, in order to explore the response of arrays of sites.

The other parameter in the equation, *ω*, was given a qualitative two-level treatment: it took value 20 for activators and 0 for repressors. Although these two fixed values are set from empirical knowledge they do not invalidate our main findings, which are related to regulatory sites bound by the same TF. As the graph in Additional file 4 shows for an example TU, variations of the value of the *ω *parameter only displace the curve of RNAP-promoter interaction probability thus attaining saturation of the promoter at lower TF concentrations. Nevertheless, the shape of the curve remains unaltered, indicating that all simulations, if performed at lower TF concentrations will render identical results. In a future extension of this work, a mechanism of finer tuning should be put in place to better represent the variety of TF-RNAP interactions, in order to realistically expand this model to promoters regulated by more than one TF. All simulations were implemented by *ad hoc *PERL scripts; individual TUs' graphs were automatically built using GNUPLOT scripts.

## Authors' contributions

AGP and VEA participated in the conception of the study. AGP designed and implemented the study and drafted the manuscript. ATV and JCV supported the project and provided meaningful guidance. VEA, ATV and JCV helped revising the manuscript. All authors have read and approved the manuscript.

## Supplementary Material

Additional file 1 **Kinetic graphs of all *E. coli *simple promoters obtained as described in the Methods section.** This file can be opened with tar. Click here for file

Additional file 2 **Boundaries imposed by the reduction of RNAP concentration to one tenth of *K*_*p *_on the sigmoid dependence of promoter occupancy probability on *K*_*d *_for A) activator sites and B) repressor sites.** This file can be open with PDF viewer. Click here for file

Additional file 3 **Results of confronting data produced by our model with microarray data from FNR-activated TUs in three experimental conditions.** This file can be open with PDF viewer. Click here for file

Additional file 4 **Polymerase-promoter probability *vs *TF concentration calculated using four different ω values.** This fail can be open with PDF viewer. Click here for file
