# Supplementary figures and images for: From sequence to dynamics: the effects of transcription factor and polymerase concentration changes on activated and repressed promoters (part 2 of 2)
Source: BMC Mol Biol. 2009 Sep 22;10:92. doi: 10.1186/1471-2199-10-92 (PMC2761915; doi:10.1186/1471-2199-10-92)

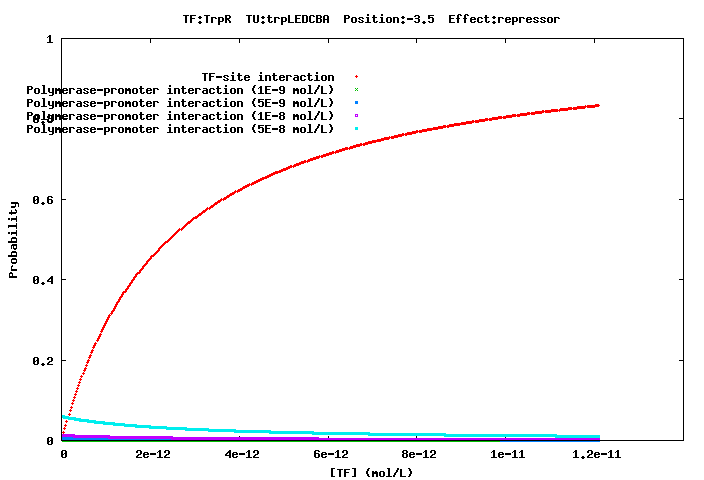

Supplement: Additional file 1 — Kinetic graphs of all E. coli simple promoters obtained as described in the Methods section. This file can be opened with tar. [file 1471-2199-10-92-S1.zip › sm1/TrpR_tcgaactagtTAACTAGTACGCAAGTTCacgtaaaaag_-3.5.pwm.plot.gif]

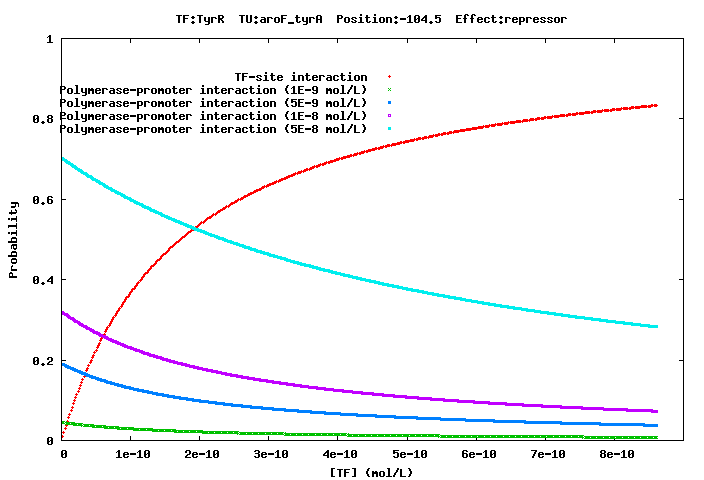

Supplement: Additional file 1 — Kinetic graphs of all E. coli simple promoters obtained as described in the Methods section. This file can be opened with tar. [file 1471-2199-10-92-S1.zip › sm1/TyrR_tcaaagggagTGTAAATTTATCTATACAgaggtaaggg_-104.5.pwm.plot.gif]

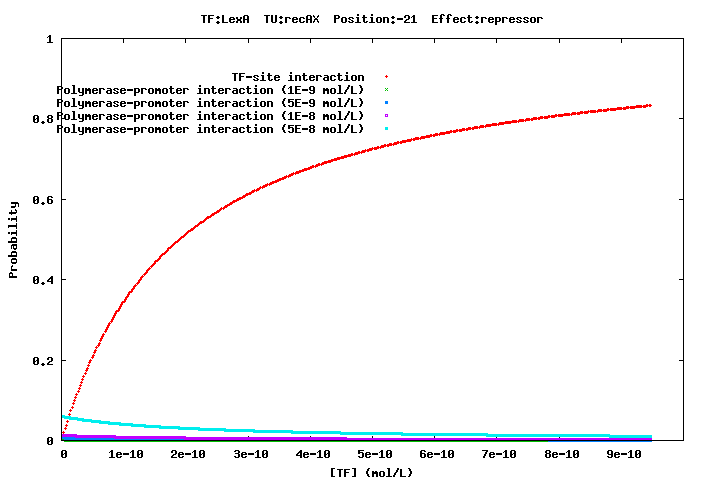

Supplement: Additional file 1 — Kinetic graphs of all E. coli simple promoters obtained as described in the Methods section. This file can be opened with tar. [file 1471-2199-10-92-S1.zip › sm1/LexA_aaacacttgaTACTGTATGAGCATACAGTAtaattgcttc_-21.pwm.plot.gif]

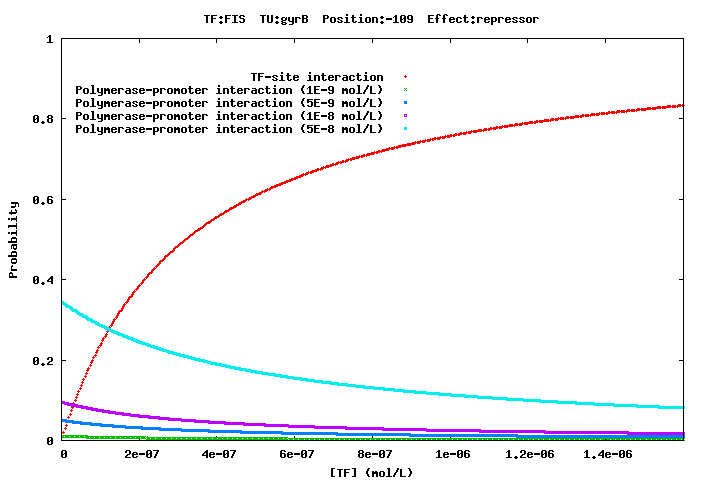

Supplement: Additional file 1 — Kinetic graphs of all E. coli simple promoters obtained as described in the Methods section. This file can be opened with tar. [file 1471-2199-10-92-S1.zip › sm1/FIS_cttgccagccGCTTAAAAGCGACGCaatcacaggt_-109.pwm.plot.gif]

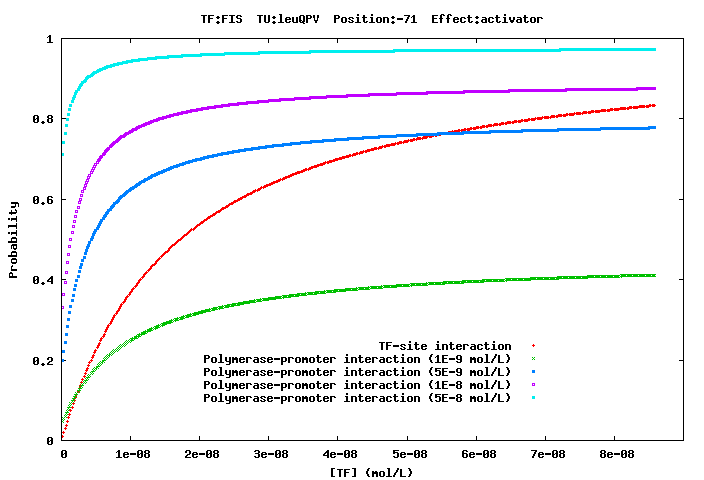

Supplement: Additional file 1 — Kinetic graphs of all E. coli simple promoters obtained as described in the Methods section. This file can be opened with tar. [file 1471-2199-10-92-S1.zip › sm1/FIS_ttcctgatggACATTTTTCCAGCAAttacacctct_-71.pwm.plot.gif]
