# Supplementary figures and images for: From sequence to dynamics: the effects of transcription factor and polymerase concentration changes on activated and repressed promoters (part 1 of 2)
Source: BMC Mol Biol. 2009 Sep 22;10:92. doi: 10.1186/1471-2199-10-92 (PMC2761915; doi:10.1186/1471-2199-10-92)

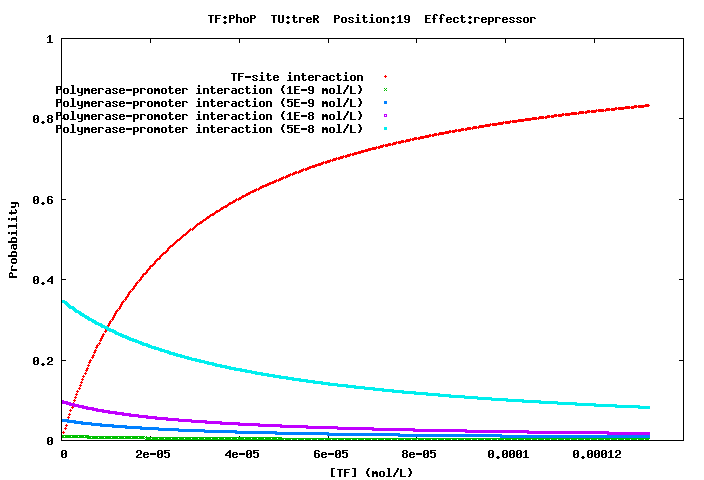

Supplement: Additional file 1 — Kinetic graphs of all E. coli simple promoters obtained as described in the Methods section. This file can be opened with tar. [file 1471-2199-10-92-S1.zip › sm1/PhoP_tgctgacaacTAAACCAACGATAAACCagactttacc_19.pwm.plot.gif]

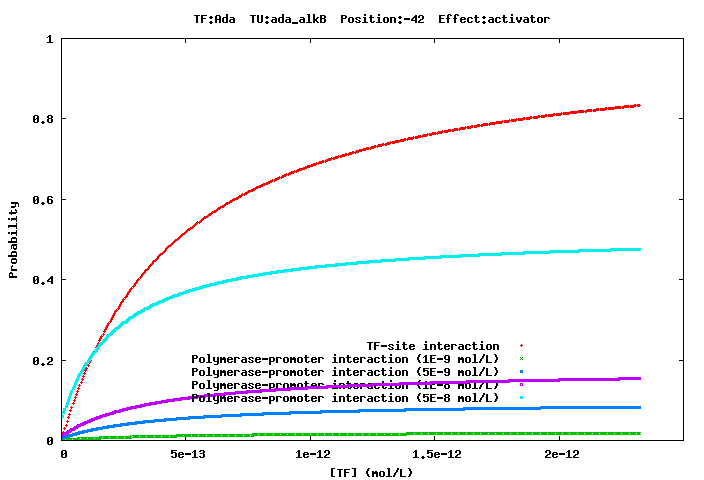

Supplement: Additional file 1 — Kinetic graphs of all E. coli simple promoters obtained as described in the Methods section. This file can be opened with tar. [file 1471-2199-10-92-S1.zip › sm1/Ada_gcgaaaaaaaTTAAAGCGCAAGATTGTTGGTTTTTGCGtgatggtgac_-42.pwm.plot.gif]

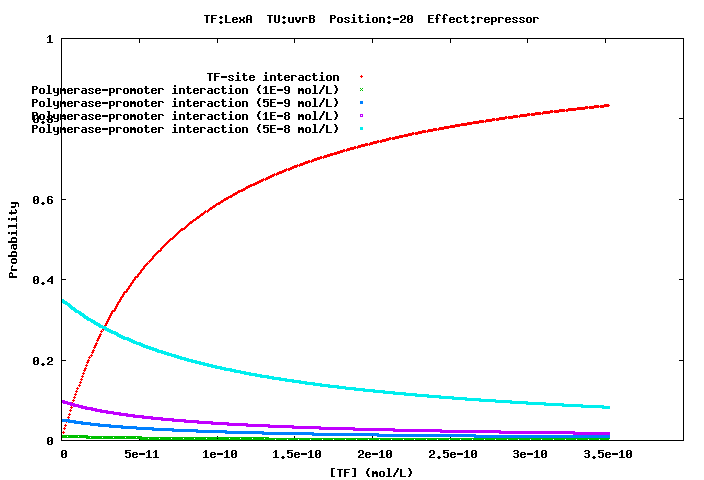

Supplement: Additional file 1 — Kinetic graphs of all E. coli simple promoters obtained as described in the Methods section. This file can be opened with tar. [file 1471-2199-10-92-S1.zip › sm1/LexA_tatggtgatgAACTGTTTTTTTATCCAGTAtaatttgttg_-20.pwm.plot.gif]

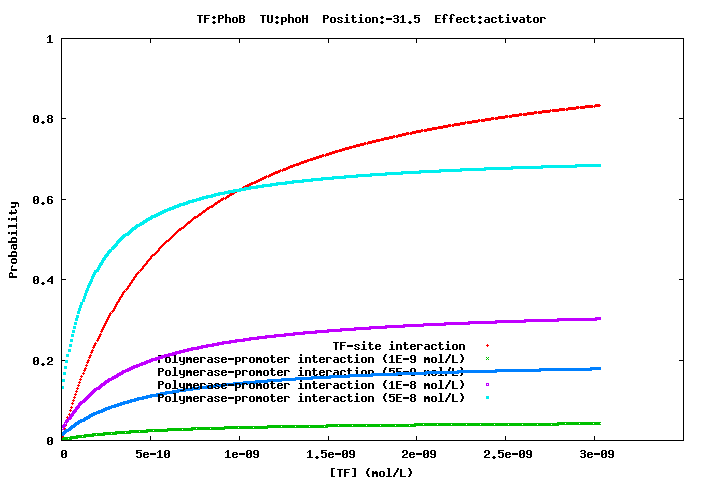

Supplement: Additional file 1 — Kinetic graphs of all E. coli simple promoters obtained as described in the Methods section. This file can be opened with tar. [file 1471-2199-10-92-S1.zip › sm1/PhoB_ttttttcatcACTGTCATCACTCTGTCATCtttccagtag_-31.5.pwm.plot.gif]

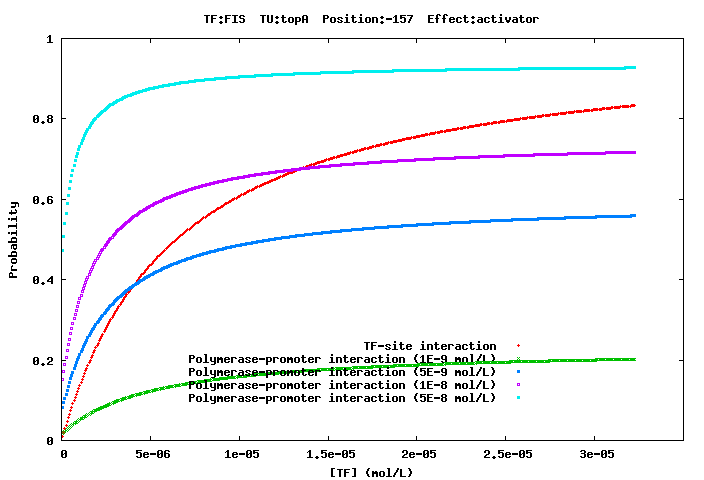

Supplement: Additional file 1 — Kinetic graphs of all E. coli simple promoters obtained as described in the Methods section. This file can be opened with tar. [file 1471-2199-10-92-S1.zip › sm1/FIS_gtacagtgtgACGCTTTCGTCAATCtggcaataga_-157.pwm.plot.gif]

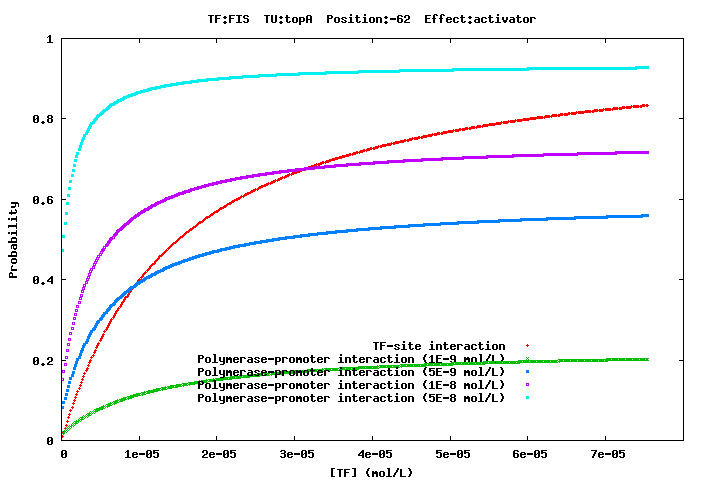

Supplement: Additional file 1 — Kinetic graphs of all E. coli simple promoters obtained as described in the Methods section. This file can be opened with tar. [file 1471-2199-10-92-S1.zip › sm1/FIS_aactcagtcaCCTGAATTTTCGTGAacagagtcac_-62.pwm.plot.gif]

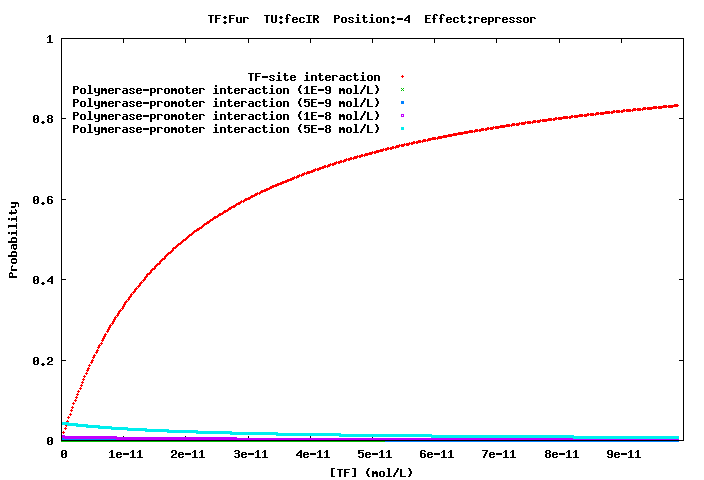

Supplement: Additional file 1 — Kinetic graphs of all E. coli simple promoters obtained as described in the Methods section. This file can be opened with tar. [file 1471-2199-10-92-S1.zip › sm1/Fur_ttccaattgtAATGATAACCATTCTCATAttaatatgac_-4.pwm.plot.gif]

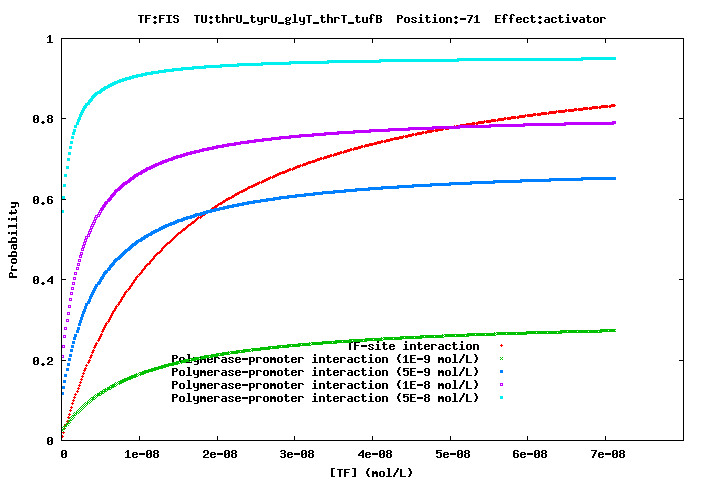

Supplement: Additional file 1 — Kinetic graphs of all E. coli simple promoters obtained as described in the Methods section. This file can be opened with tar. [file 1471-2199-10-92-S1.zip › sm1/FIS_tcatcgtgtcGCATAAAATGTGACCaataaaacaa_-71.pwm.plot.gif]

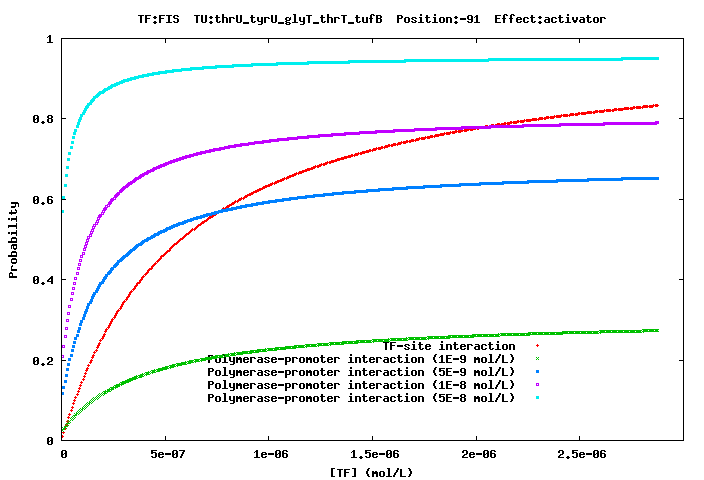

Supplement: Additional file 1 — Kinetic graphs of all E. coli simple promoters obtained as described in the Methods section. This file can be opened with tar. [file 1471-2199-10-92-S1.zip › sm1/FIS_ccctccgttcGGCTGTTTCTTCATCgtgtcgcata_-91.pwm.plot.gif]

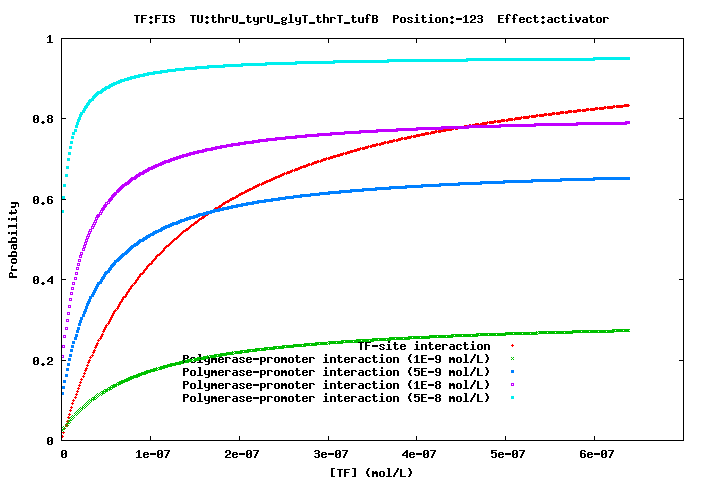

Supplement: Additional file 1 — Kinetic graphs of all E. coli simple promoters obtained as described in the Methods section. This file can be opened with tar. [file 1471-2199-10-92-S1.zip › sm1/FIS_gcacaatgatGTTGAAAAAGTGTGCtaatctgccc_-123.pwm.plot.gif]

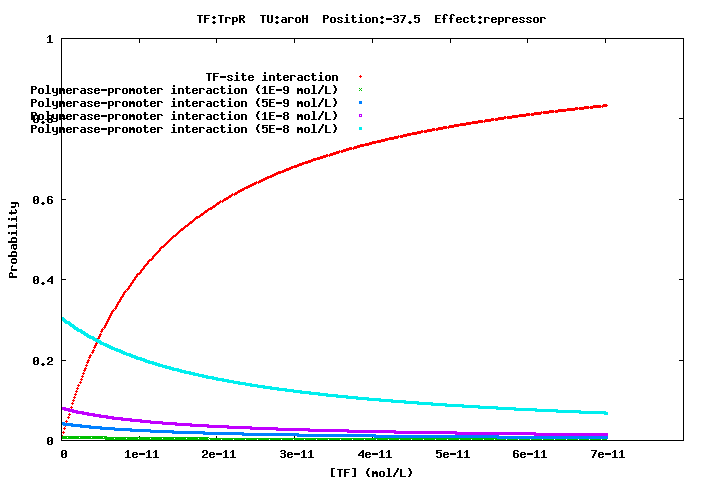

Supplement: Additional file 1 — Kinetic graphs of all E. coli simple promoters obtained as described in the Methods section. This file can be opened with tar. [file 1471-2199-10-92-S1.zip › sm1/TrpR_gtcgccgaatGTACTAGAGAACTAGTGCattagcttat_-37.5.pwm.plot.gif]

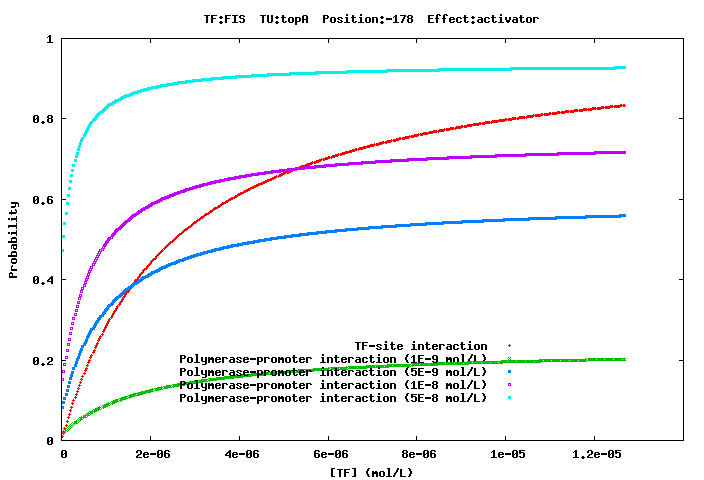

Supplement: Additional file 1 — Kinetic graphs of all E. coli simple promoters obtained as described in the Methods section. This file can be opened with tar. [file 1471-2199-10-92-S1.zip › sm1/FIS_ttgcgtatcgGATTTTATCAGGTACagtgtgacgc_-178.pwm.plot.gif]

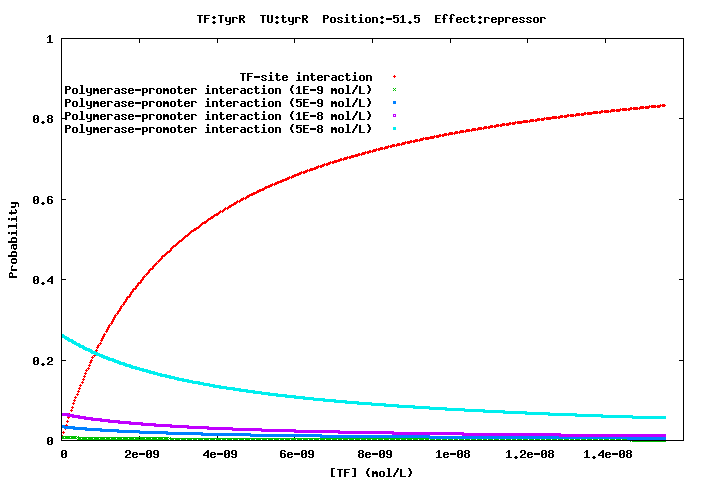

Supplement: Additional file 1 — Kinetic graphs of all E. coli simple promoters obtained as described in the Methods section. This file can be opened with tar. [file 1471-2199-10-92-S1.zip › sm1/TyrR_tccgtctttgTGTCAATGATTGTTGACAgaaaccttcc_-51.5.pwm.plot.gif]

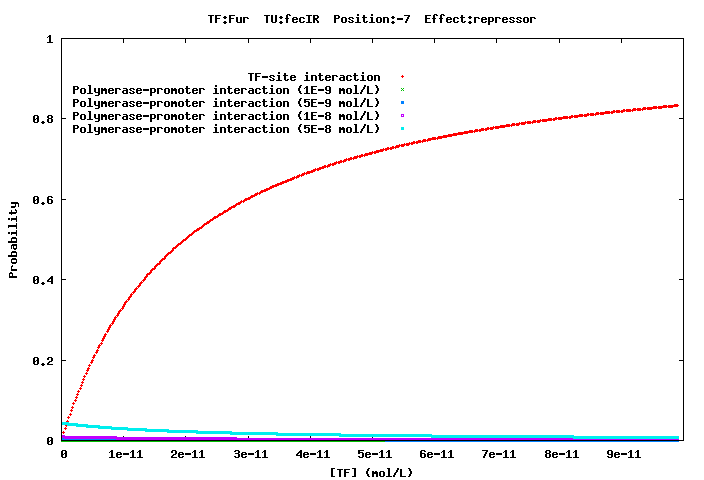

Supplement: Additional file 1 — Kinetic graphs of all E. coli simple promoters obtained as described in the Methods section. This file can be opened with tar. [file 1471-2199-10-92-S1.zip › sm1/Fur_tatttccaatTGTAATGATAACCATTCTCatattaatat_-7.pwm.plot.gif]

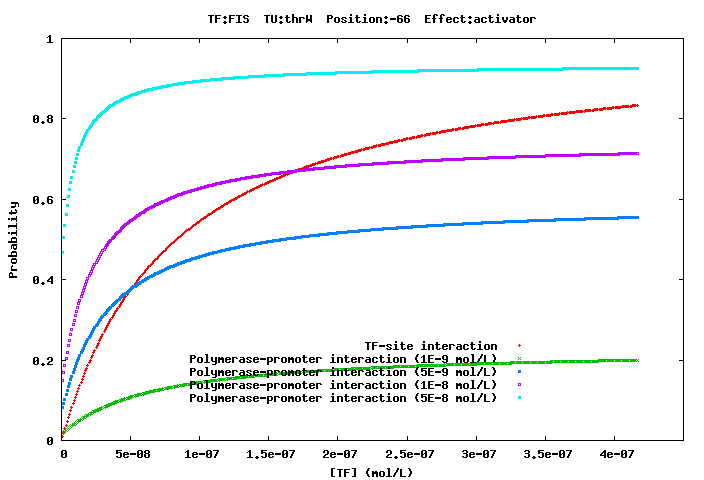

Supplement: Additional file 1 — Kinetic graphs of all E. coli simple promoters obtained as described in the Methods section. This file can be opened with tar. [file 1471-2199-10-92-S1.zip › sm1/FIS_taaaaccgggTGATGCAAAAGTAGCcatttgattc_-66.pwm.plot.gif]

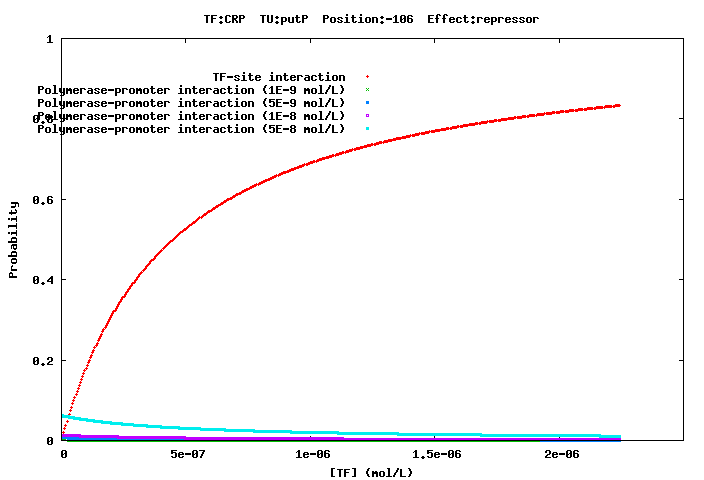

Supplement: Additional file 1 — Kinetic graphs of all E. coli simple promoters obtained as described in the Methods section. This file can be opened with tar. [file 1471-2199-10-92-S1.zip › sm1/CRP_aggtgcaaccGCAAAAAATGTGAGAGAGTGCAacctgatgaa_-106.pwm.plot.gif]

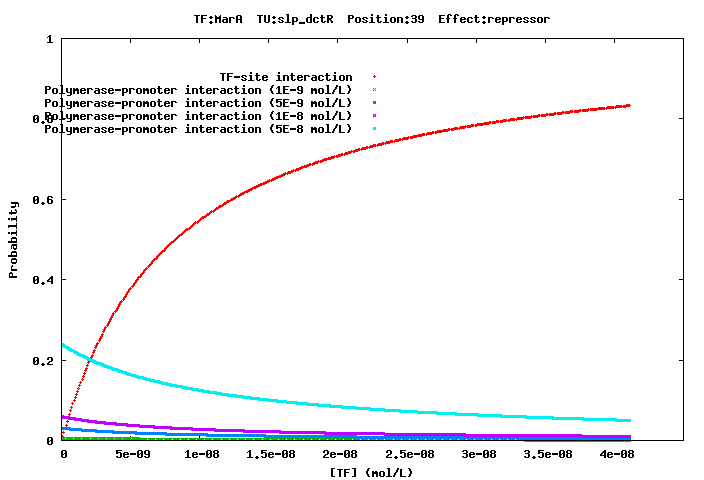

Supplement: Additional file 1 — Kinetic graphs of all E. coli simple promoters obtained as described in the Methods section. This file can be opened with tar. [file 1471-2199-10-92-S1.zip › sm1/MarA_tagtaacatgAACATGACAAAAGGTGCACTCatcctcagcc_39.pwm.plot.gif]

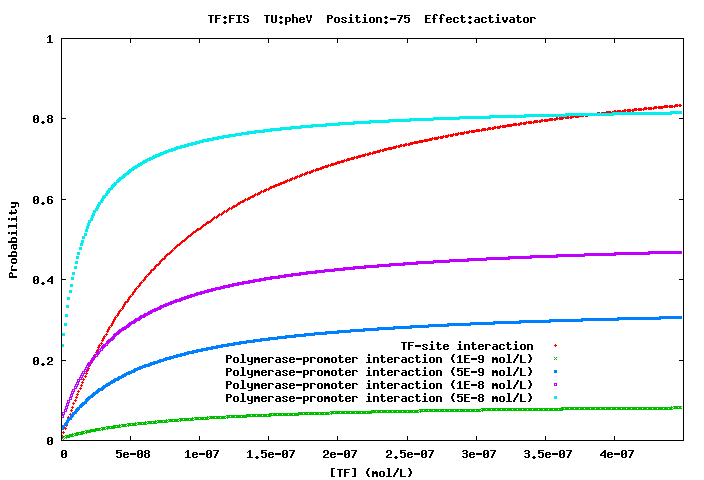

Supplement: Additional file 1 — Kinetic graphs of all E. coli simple promoters obtained as described in the Methods section. This file can be opened with tar. [file 1471-2199-10-92-S1.zip › sm1/FIS_tatcggcaaaGTGATGATAGATTGTgcagtctgca_-75.pwm.plot.gif]

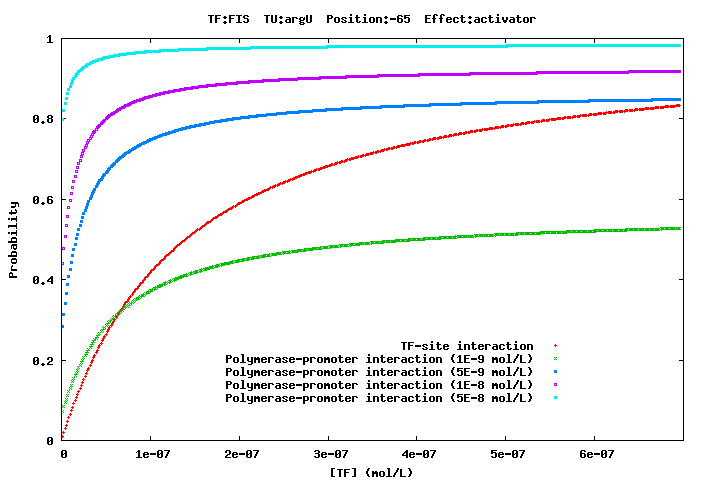

Supplement: Additional file 1 — Kinetic graphs of all E. coli simple promoters obtained as described in the Methods section. This file can be opened with tar. [file 1471-2199-10-92-S1.zip › sm1/FIS_cagacgcggtCGTTCACTTGTTCAGcaaccagatc_-65.pwm.plot.gif]

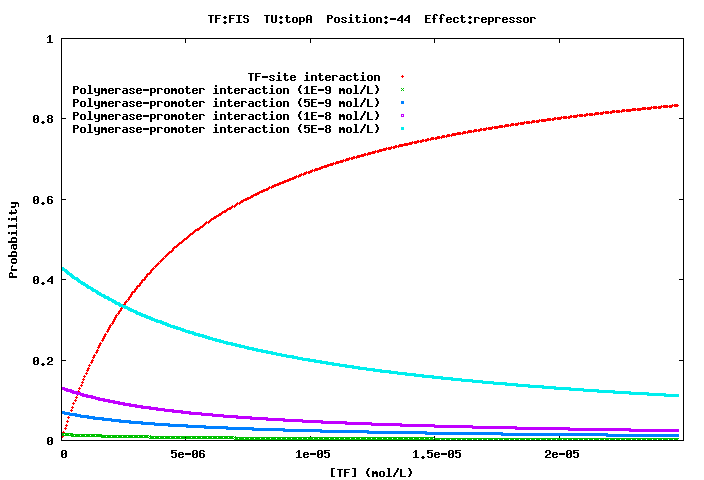

Supplement: Additional file 1 — Kinetic graphs of all E. coli simple promoters obtained as described in the Methods section. This file can be opened with tar. [file 1471-2199-10-92-S1.zip › sm1/FIS_ggattttgcaTGCTAATAAAGTTGCgtatcggatt_-44.pwm.plot.gif]

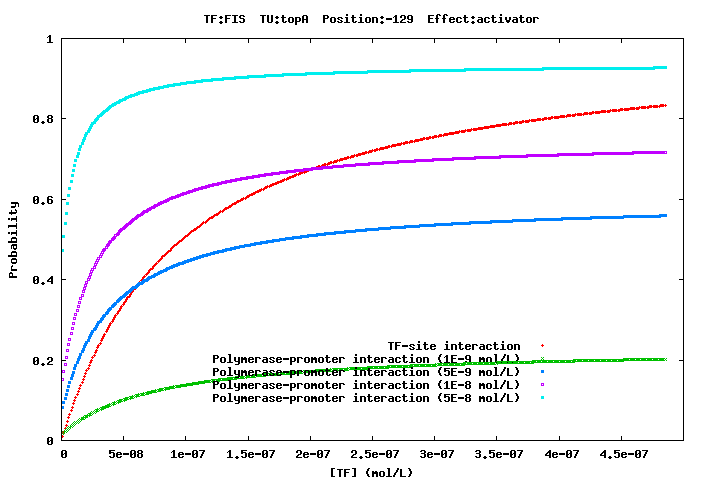

Supplement: Additional file 1 — Kinetic graphs of all E. coli simple promoters obtained as described in the Methods section. This file can be opened with tar. [file 1471-2199-10-92-S1.zip › sm1/FIS_caatagatttGCTTGACATTCGACCaaaattccgt_-129.pwm.plot.gif]

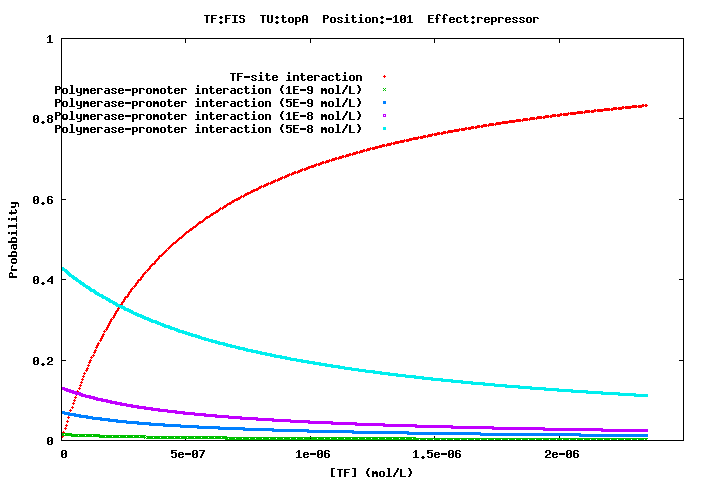

Supplement: Additional file 1 — Kinetic graphs of all E. coli simple promoters obtained as described in the Methods section. This file can be opened with tar. [file 1471-2199-10-92-S1.zip › sm1/FIS_catggcttgcAGACAAATATACCACgctggtggca_-101.pwm.plot.gif]

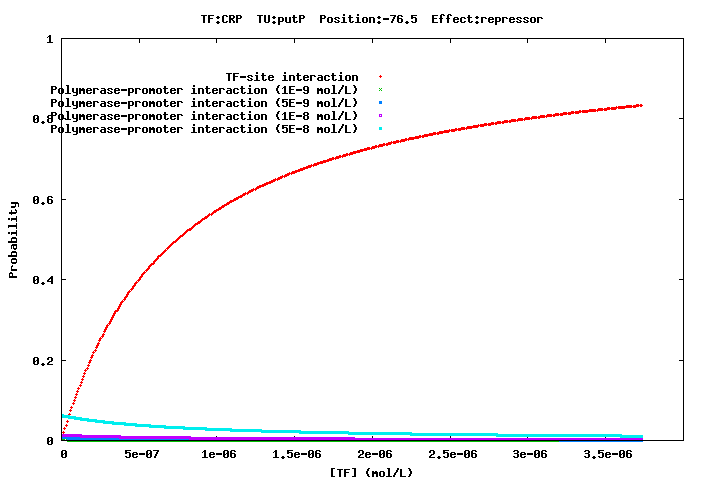

Supplement: Additional file 1 — Kinetic graphs of all E. coli simple promoters obtained as described in the Methods section. This file can be opened with tar. [file 1471-2199-10-92-S1.zip › sm1/CRP_caacctgatgAAAAATAGTGTCGCTGAGCACTaaaatttaat_-76.5.pwm.plot.gif]

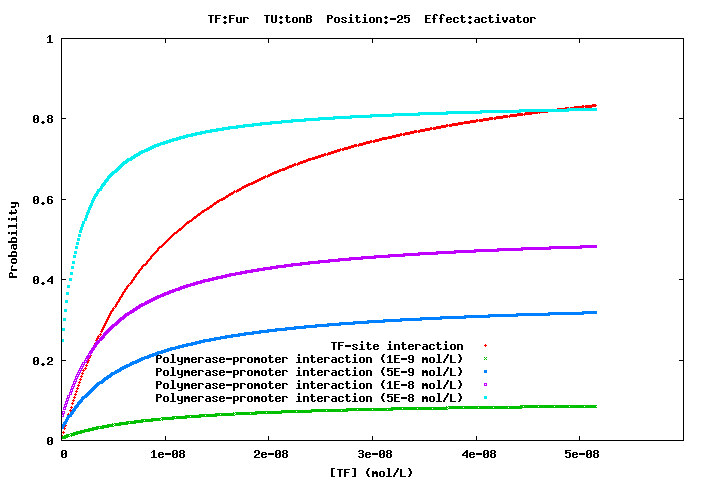

Supplement: Additional file 1 — Kinetic graphs of all E. coli simple promoters obtained as described in the Methods section. This file can be opened with tar. [file 1471-2199-10-92-S1.zip › sm1/Fur_gtcttgccttATTGAATATGATTGCTATTtgcatttaaa_-25.pwm.plot.gif]

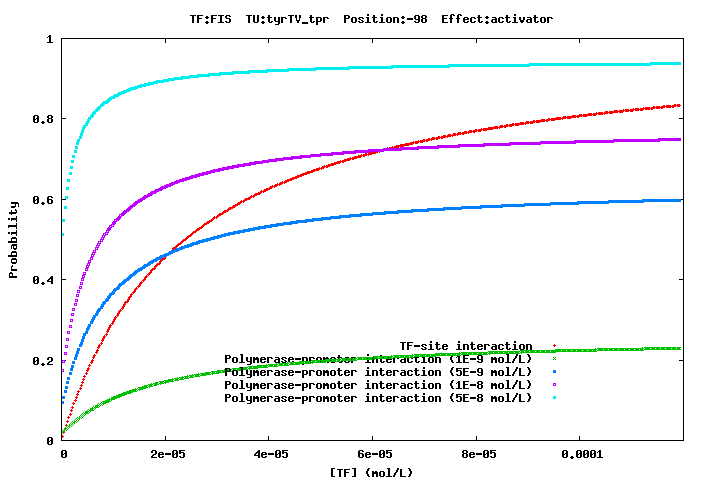

Supplement: Additional file 1 — Kinetic graphs of all E. coli simple promoters obtained as described in the Methods section. This file can be opened with tar. [file 1471-2199-10-92-S1.zip › sm1/FIS_cgccagcaaaAATAACTGGTTACCTttaatccgtt_-98.pwm.plot.gif]

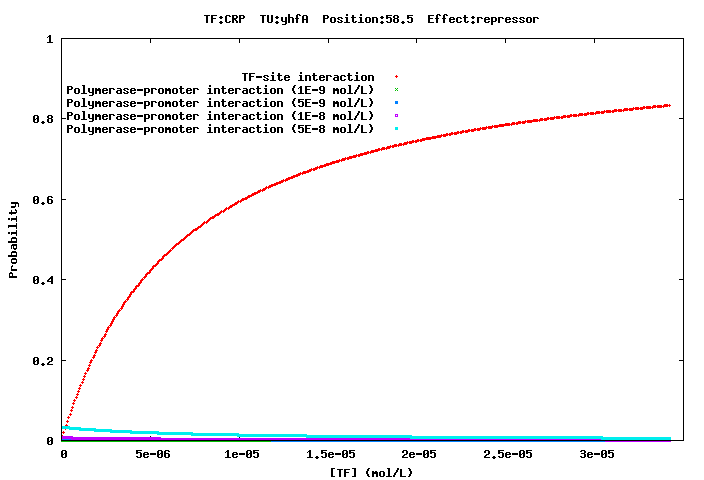

Supplement: Additional file 1 — Kinetic graphs of all E. coli simple promoters obtained as described in the Methods section. This file can be opened with tar. [file 1471-2199-10-92-S1.zip › sm1/CRP_gtgtctcgctTCAGCATGACCCAGGTCGCCTTccgttgcgcg_58.5.pwm.plot.gif]

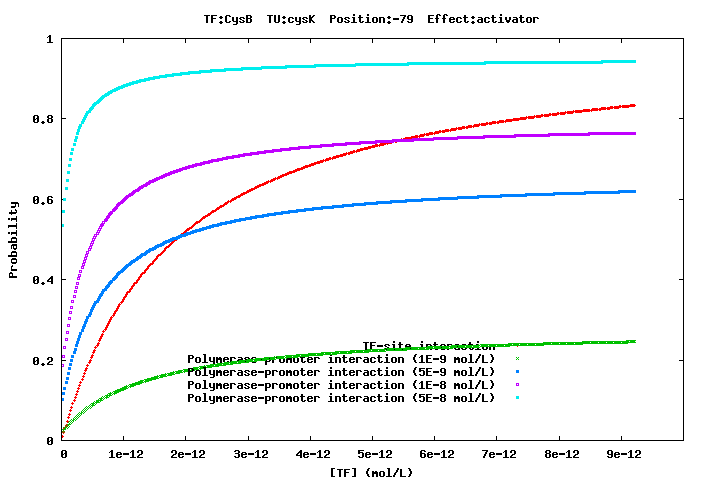

Supplement: Additional file 1 — Kinetic graphs of all E. coli simple promoters obtained as described in the Methods section. This file can be opened with tar. [file 1471-2199-10-92-S1.zip › sm1/CysB_gtggcttatgCCGCCCCTTATTCCATCTTGCATGTCATTATTTCCCTTCTGTatatagatat_-79.pwm.plot.gif]

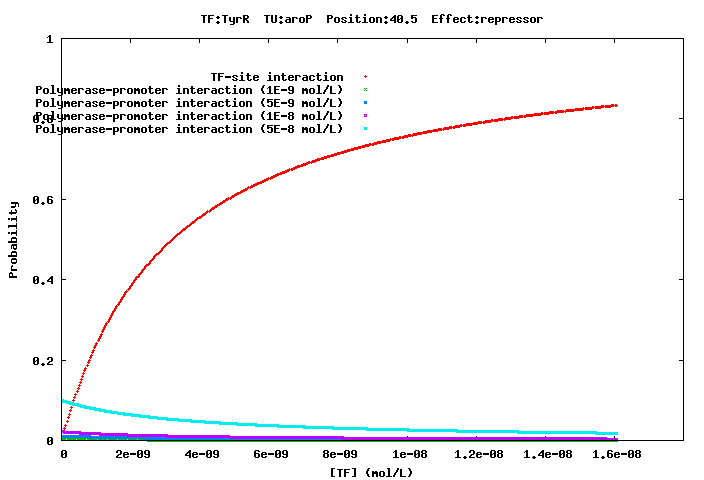

Supplement: Additional file 1 — Kinetic graphs of all E. coli simple promoters obtained as described in the Methods section. This file can be opened with tar. [file 1471-2199-10-92-S1.zip › sm1/TyrR_aacttctttgATGTAAACAAATTAATACaacaaacgga_40.5.pwm.plot.gif]

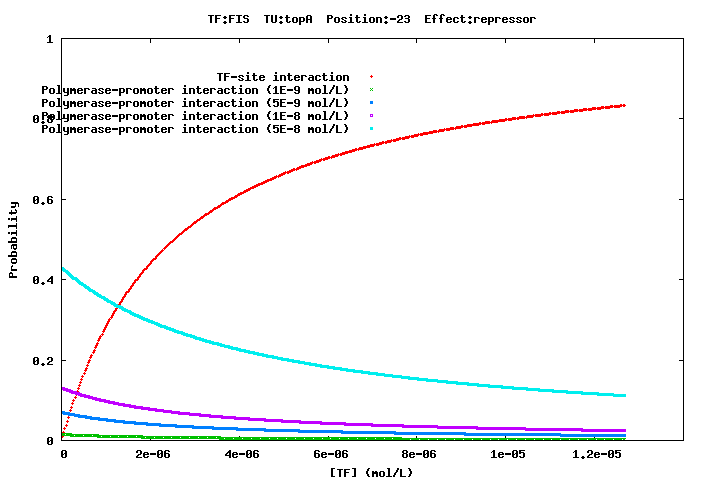

Supplement: Additional file 1 — Kinetic graphs of all E. coli simple promoters obtained as described in the Methods section. This file can be opened with tar. [file 1471-2199-10-92-S1.zip › sm1/FIS_ttgcgtatcgGATTTTATCAGGTACagtgtgacgc_-23.pwm.plot.gif]

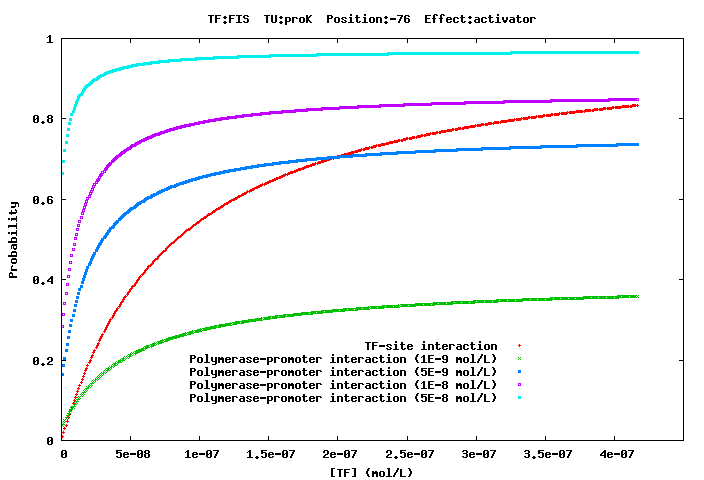

Supplement: Additional file 1 — Kinetic graphs of all E. coli simple promoters obtained as described in the Methods section. This file can be opened with tar. [file 1471-2199-10-92-S1.zip › sm1/FIS_ctaagcggccTGCTGACTTTCTCGCcgatcaaaag_-76.pwm.plot.gif]

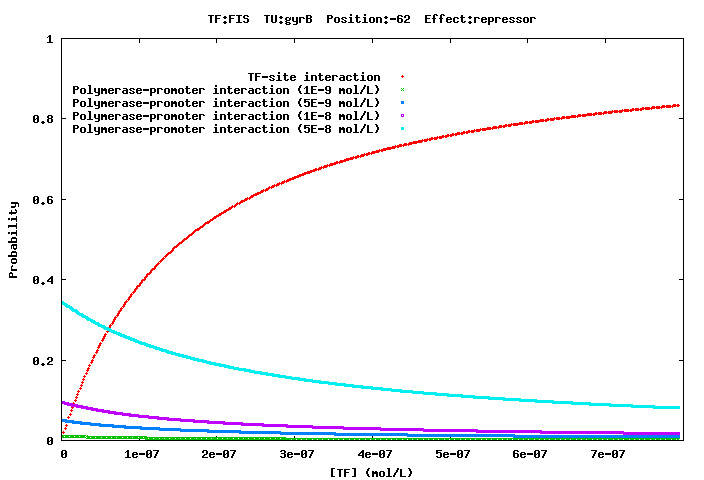

Supplement: Additional file 1 — Kinetic graphs of all E. coli simple promoters obtained as described in the Methods section. This file can be opened with tar. [file 1471-2199-10-92-S1.zip › sm1/FIS_gatcagtgctGAACACGTTATAGACatgtcggacg_-62.pwm.plot.gif]

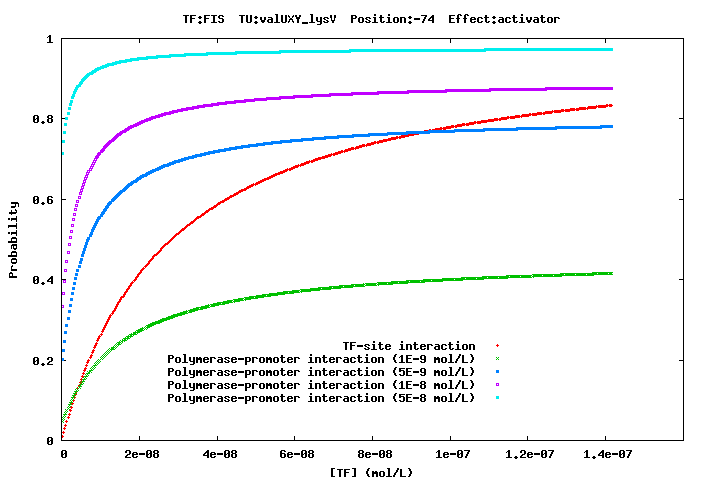

Supplement: Additional file 1 — Kinetic graphs of all E. coli simple promoters obtained as described in the Methods section. This file can be opened with tar. [file 1471-2199-10-92-S1.zip › sm1/FIS_gcgatcatctTGCTTAAATTTACGAcgaacgaaca_-74.pwm.plot.gif]

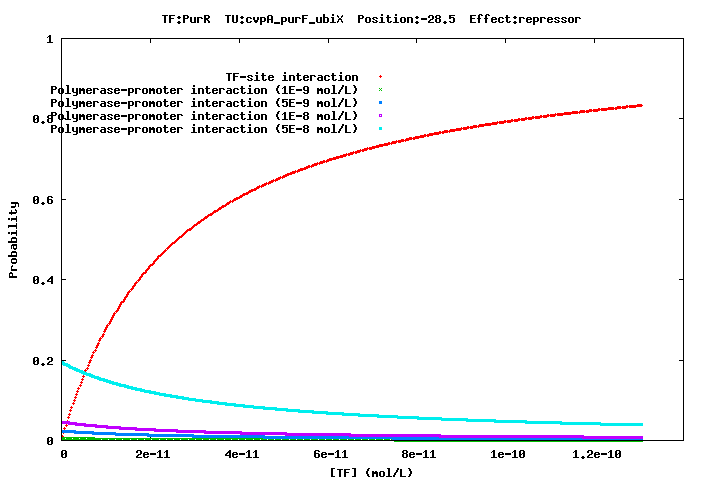

Supplement: Additional file 1 — Kinetic graphs of all E. coli simple promoters obtained as described in the Methods section. This file can be opened with tar. [file 1471-2199-10-92-S1.zip › sm1/PurR_aggaaatcccTACGCAAACGTTTTCTttttctgtta_-28.5.pwm.plot.gif]

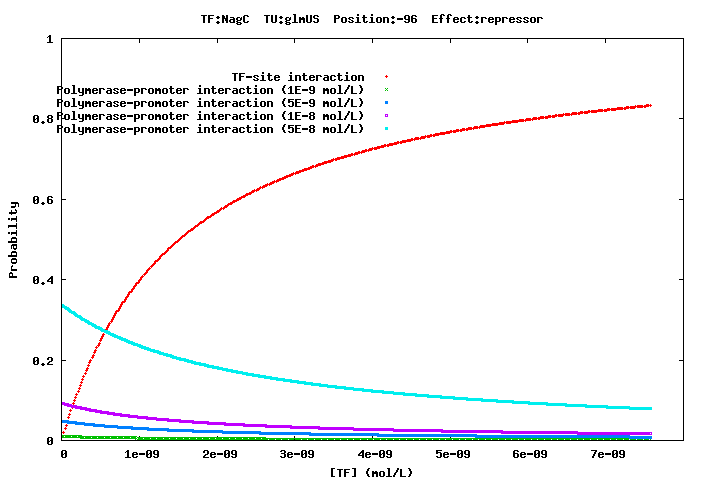

Supplement: Additional file 1 — Kinetic graphs of all E. coli simple promoters obtained as described in the Methods section. This file can be opened with tar. [file 1471-2199-10-92-S1.zip › sm1/NagC_ctgttttcctGTTTATTCATTGATCGAAATAAGagcaaaaaca_-96.pwm.plot.gif]

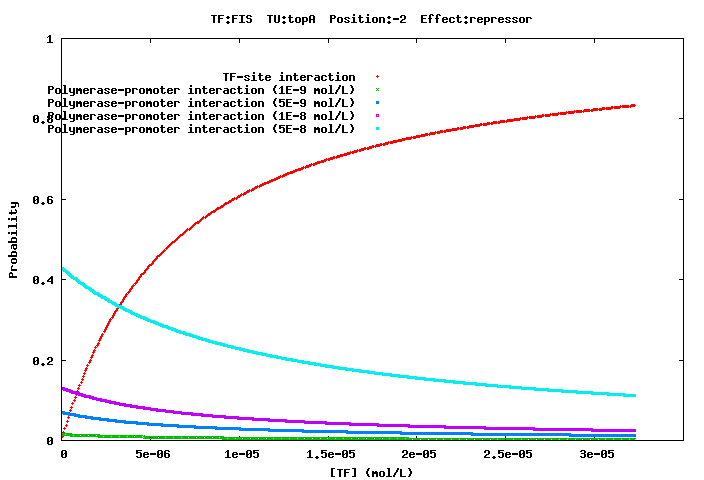

Supplement: Additional file 1 — Kinetic graphs of all E. coli simple promoters obtained as described in the Methods section. This file can be opened with tar. [file 1471-2199-10-92-S1.zip › sm1/FIS_gtacagtgtgACGCTTTCGTCAATCtggcaataga_-2.pwm.plot.gif]

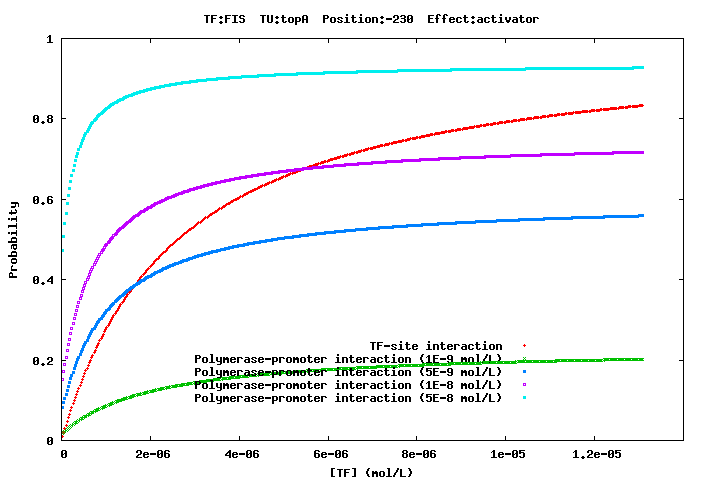

Supplement: Additional file 1 — Kinetic graphs of all E. coli simple promoters obtained as described in the Methods section. This file can be opened with tar. [file 1471-2199-10-92-S1.zip › sm1/FIS_ctggtggcaaGAGCGCCTTACTGGCaactttggat_-230.pwm.plot.gif]

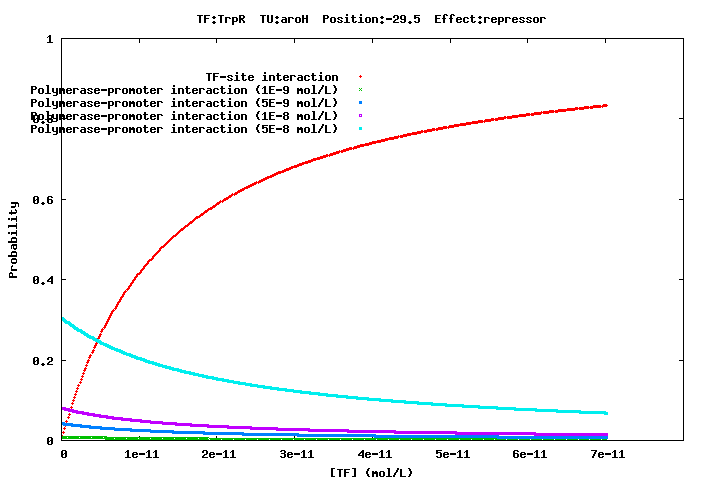

Supplement: Additional file 1 — Kinetic graphs of all E. coli simple promoters obtained as described in the Methods section. This file can be opened with tar. [file 1471-2199-10-92-S1.zip › sm1/TrpR_atgtactagaGAACTAGTGCATTAGCTTatttttttgt_-29.5.pwm.plot.gif]

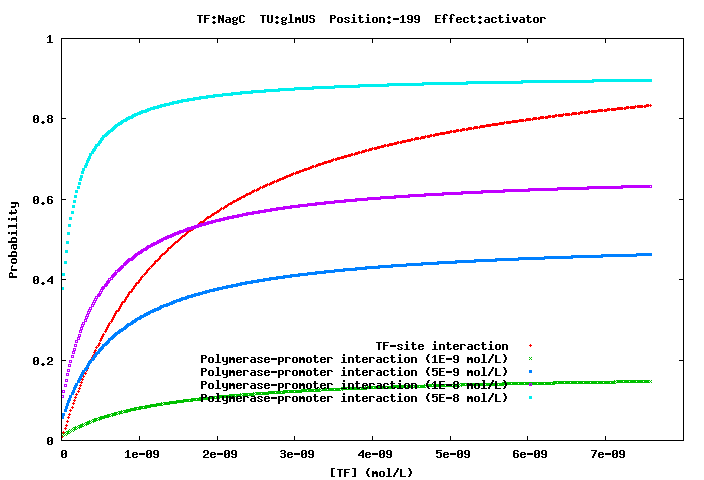

Supplement: Additional file 1 — Kinetic graphs of all E. coli simple promoters obtained as described in the Methods section. This file can be opened with tar. [file 1471-2199-10-92-S1.zip › sm1/NagC_ctgttttcctGTTTATTCATTGATCGAAATAAGagcaaaaaca_-199.pwm.plot.gif]

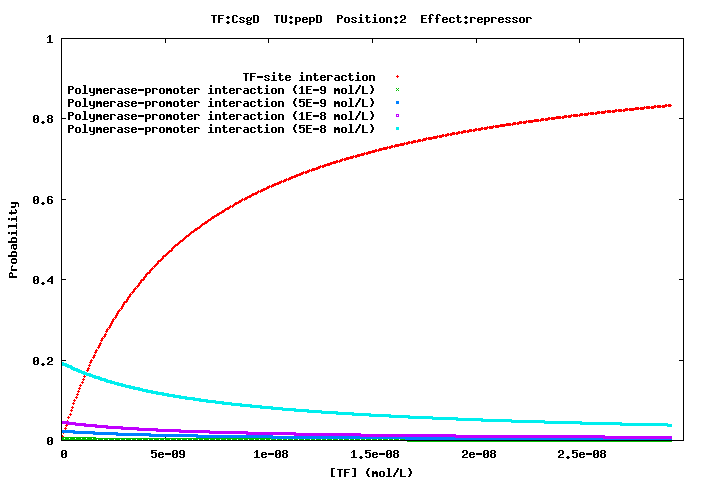

Supplement: Additional file 1 — Kinetic graphs of all E. coli simple promoters obtained as described in the Methods section. This file can be opened with tar. [file 1471-2199-10-92-S1.zip › sm1/CsgD_tactgttttgCGGGTGATCGAcaaggagact_2.pwm.plot.gif]

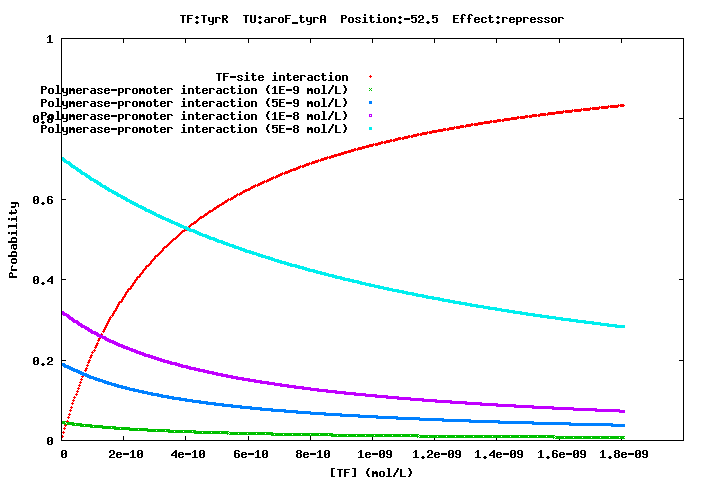

Supplement: Additional file 1 — Kinetic graphs of all E. coli simple promoters obtained as described in the Methods section. This file can be opened with tar. [file 1471-2199-10-92-S1.zip › sm1/TyrR_aaattgcctgTGTAAATAAAAATGTACGaaatatggat_-52.5.pwm.plot.gif]

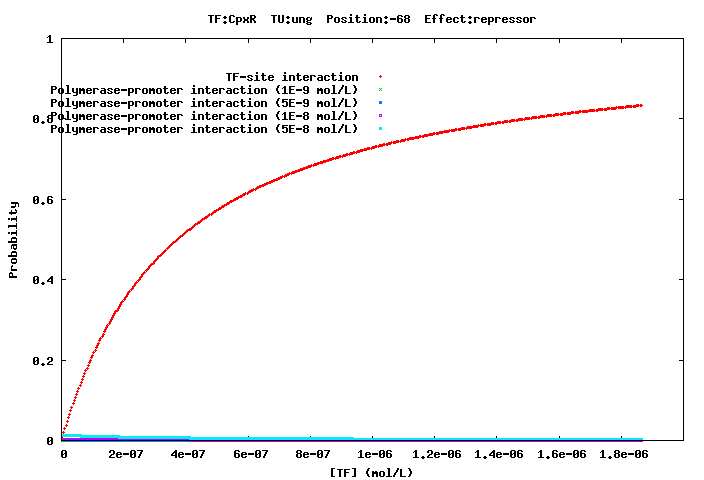

Supplement: Additional file 1 — Kinetic graphs of all E. coli simple promoters obtained as described in the Methods section. This file can be opened with tar. [file 1471-2199-10-92-S1.zip › sm1/CpxR_acatttttttGCACTCGTTTAAGTCtaaaaaatga_-68.pwm.plot.gif]

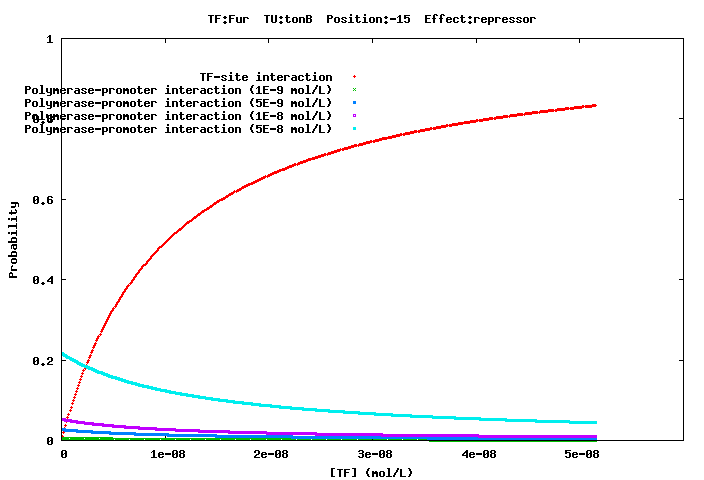

Supplement: Additional file 1 — Kinetic graphs of all E. coli simple promoters obtained as described in the Methods section. This file can be opened with tar. [file 1471-2199-10-92-S1.zip › sm1/Fur_attgaatatgATTGCTATTTGCATTTAAAatcgagacct_-15.pwm.plot.gif]

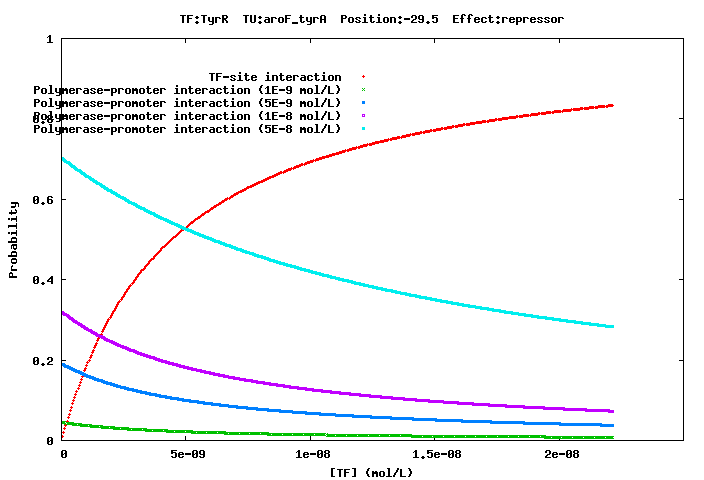

Supplement: Additional file 1 — Kinetic graphs of all E. coli simple promoters obtained as described in the Methods section. This file can be opened with tar. [file 1471-2199-10-92-S1.zip › sm1/TyrR_gtacgaaataTGGATTGAAAACTTTACTttatgtgtta_-29.5.pwm.plot.gif]

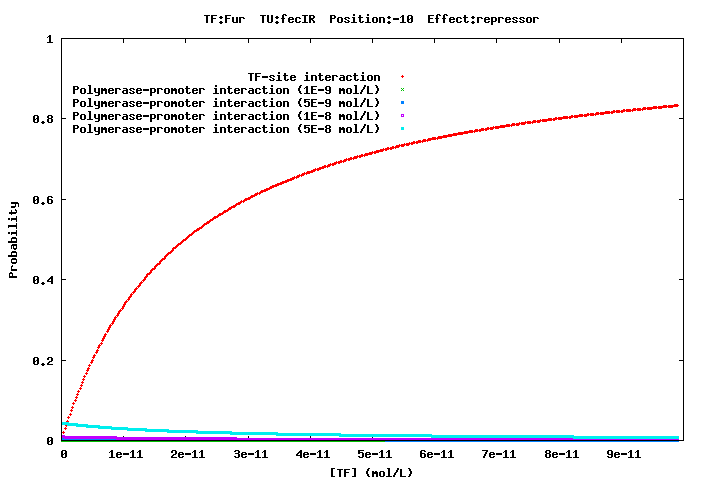

Supplement: Additional file 1 — Kinetic graphs of all E. coli simple promoters obtained as described in the Methods section. This file can be opened with tar. [file 1471-2199-10-92-S1.zip › sm1/Fur_ttttatttccAATTGTAATGATAACCATTctcatattaa_-10.pwm.plot.gif]

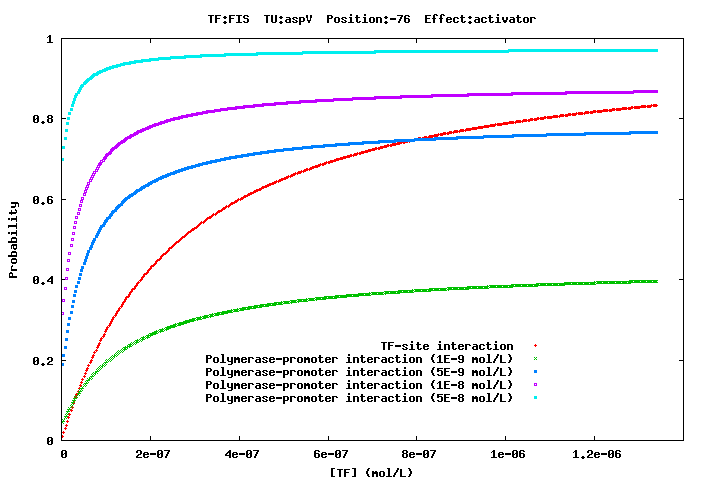

Supplement: Additional file 1 — Kinetic graphs of all E. coli simple promoters obtained as described in the Methods section. This file can be opened with tar. [file 1471-2199-10-92-S1.zip › sm1/FIS_aatagcgactTGGGCGATTTTTGCAgcaaacgatt_-76.pwm.plot.gif]

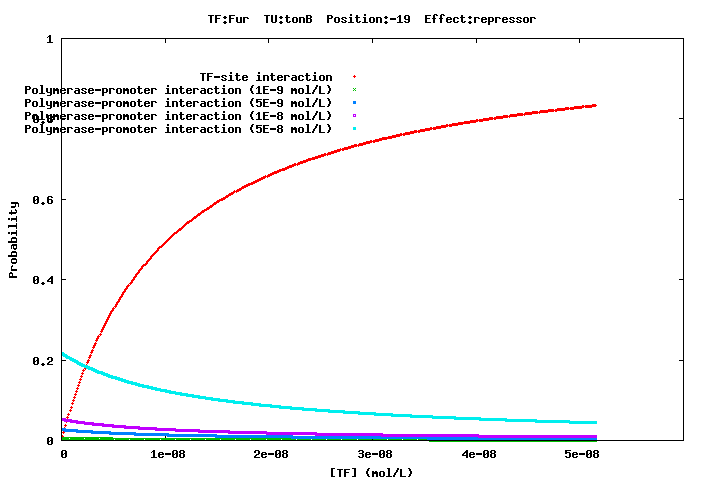

Supplement: Additional file 1 — Kinetic graphs of all E. coli simple promoters obtained as described in the Methods section. This file can be opened with tar. [file 1471-2199-10-92-S1.zip › sm1/Fur_ccttattgaaTATGATTGCTATTTGCATTtaaaatcgag_-19.pwm.plot.gif]

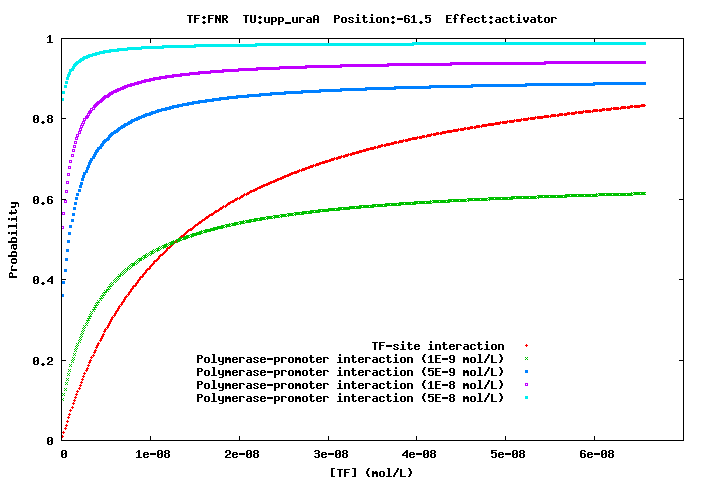

Supplement: Additional file 1 — Kinetic graphs of all E. coli simple promoters obtained as described in the Methods section. This file can be opened with tar. [file 1471-2199-10-92-S1.zip › sm1/FNR_tatctcaaacCGTTATCATTTTGActaaagtcaa_-61.5.pwm.plot.gif]

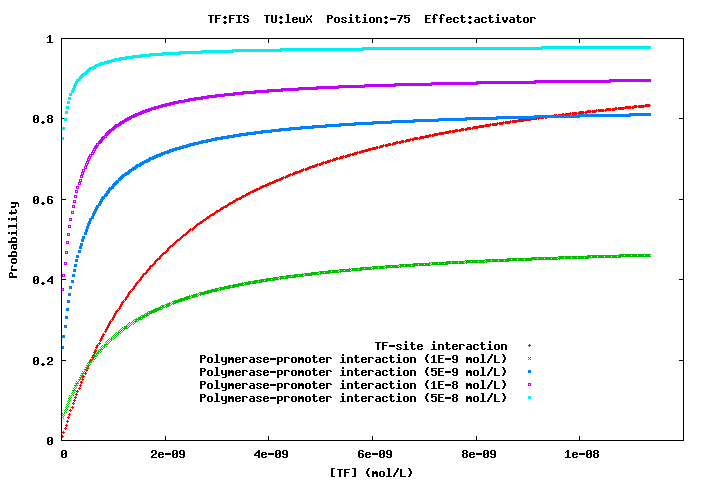

Supplement: Additional file 1 — Kinetic graphs of all E. coli simple promoters obtained as described in the Methods section. This file can be opened with tar. [file 1471-2199-10-92-S1.zip › sm1/FIS_ctgtatgcaaTGCTGAAAATTTCAGcacttagcga_-75.pwm.plot.gif]

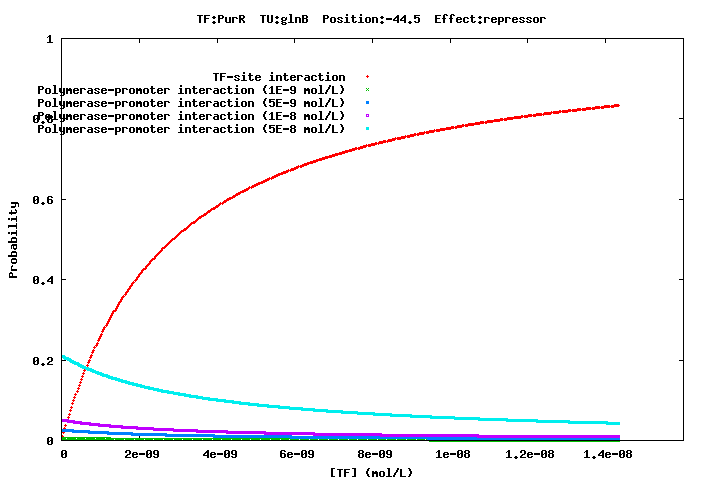

Supplement: Additional file 1 — Kinetic graphs of all E. coli simple promoters obtained as described in the Methods section. This file can be opened with tar. [file 1471-2199-10-92-S1.zip › sm1/PurR_gacacgagctGGATGCAAACGATTTCaaggaatgaa_-44.5.pwm.plot.gif]

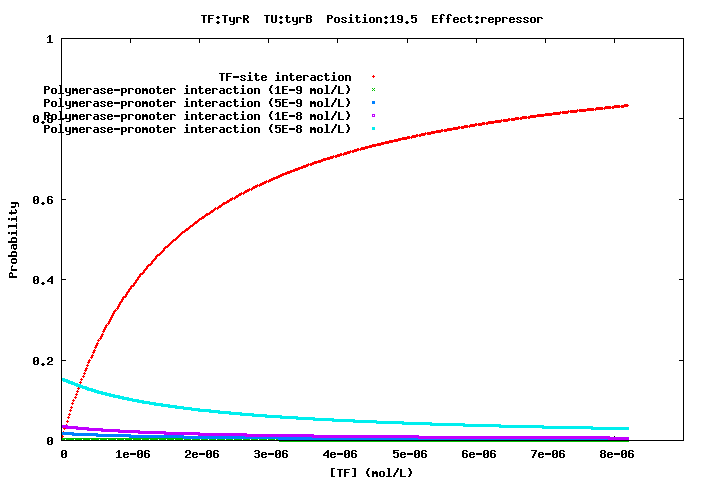

Supplement: Additional file 1 — Kinetic graphs of all E. coli simple promoters obtained as described in the Methods section. This file can be opened with tar. [file 1471-2199-10-92-S1.zip › sm1/TyrR_accacctgccCGTAAACCTGGAGAACCAtcgcgtgttt_19.5.pwm.plot.gif]

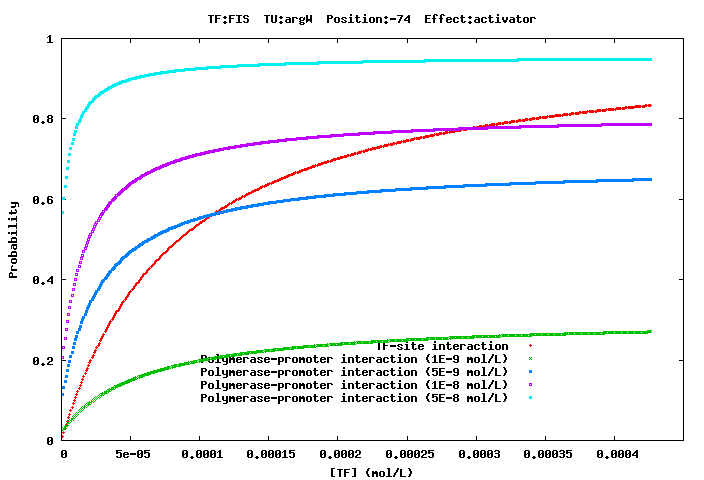

Supplement: Additional file 1 — Kinetic graphs of all E. coli simple promoters obtained as described in the Methods section. This file can be opened with tar. [file 1471-2199-10-92-S1.zip › sm1/FIS_ataaaaacccGGCATAAATGGCGAGggtttaagca_-74.pwm.plot.gif]

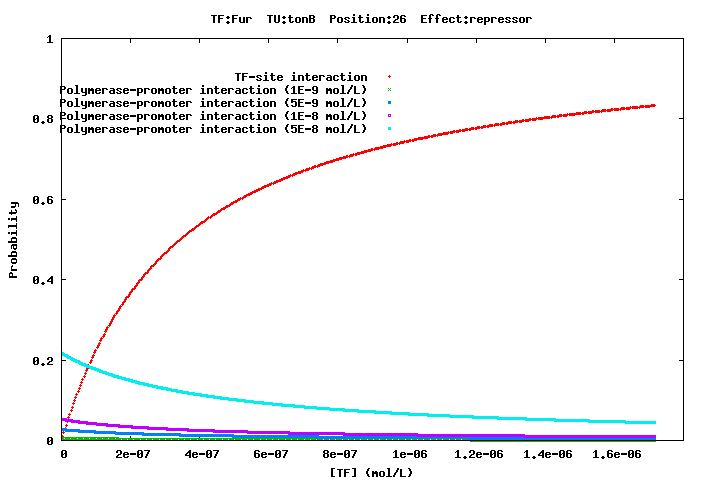

Supplement: Additional file 1 — Kinetic graphs of all E. coli simple promoters obtained as described in the Methods section. This file can be opened with tar. [file 1471-2199-10-92-S1.zip › sm1/Fur_gtttttctacTGAAATGATTATGACTTCAatgacccttg_26.pwm.plot.gif]

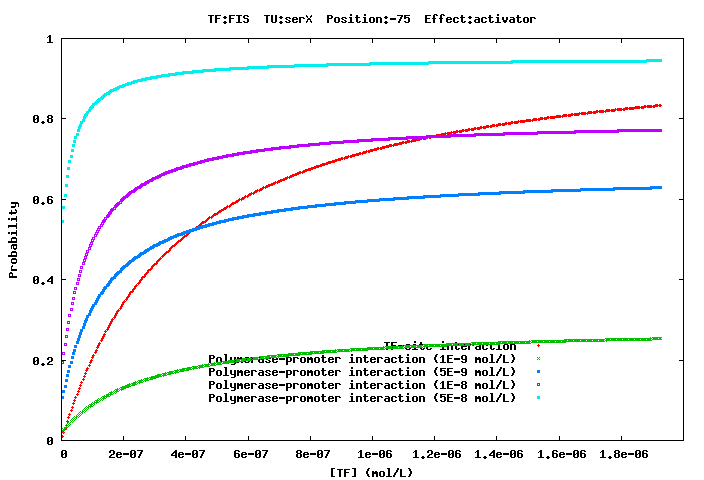

Supplement: Additional file 1 — Kinetic graphs of all E. coli simple promoters obtained as described in the Methods section. This file can be opened with tar. [file 1471-2199-10-92-S1.zip › sm1/FIS_aaccacacatTGATTGAAGTTTGAAtaaacgcgcg_-75.pwm.plot.gif]

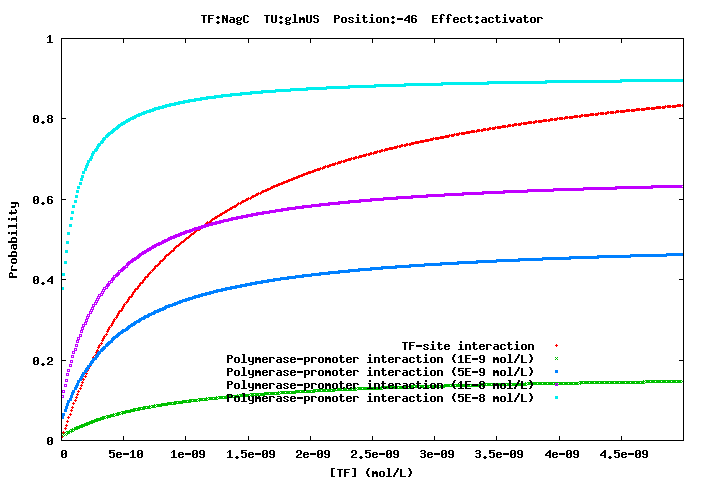

Supplement: Additional file 1 — Kinetic graphs of all E. coli simple promoters obtained as described in the Methods section. This file can be opened with tar. [file 1471-2199-10-92-S1.zip › sm1/NagC_ttatcctctgTCCATTTCACGATGAAAAAAATGtagttttttc_-46.pwm.plot.gif]

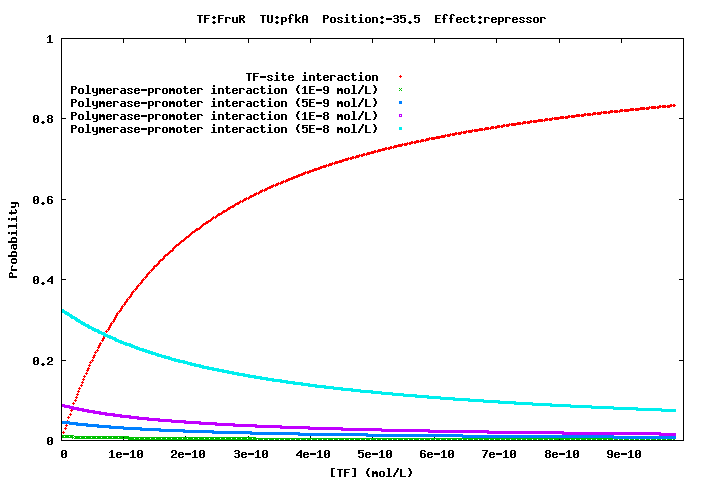

Supplement: Additional file 1 — Kinetic graphs of all E. coli simple promoters obtained as described in the Methods section. This file can be opened with tar. [file 1471-2199-10-92-S1.zip › sm1/FruR_atttggcctgACCTGAATCAATTCAGCAggaagtgatt_-35.5.pwm.plot.gif]

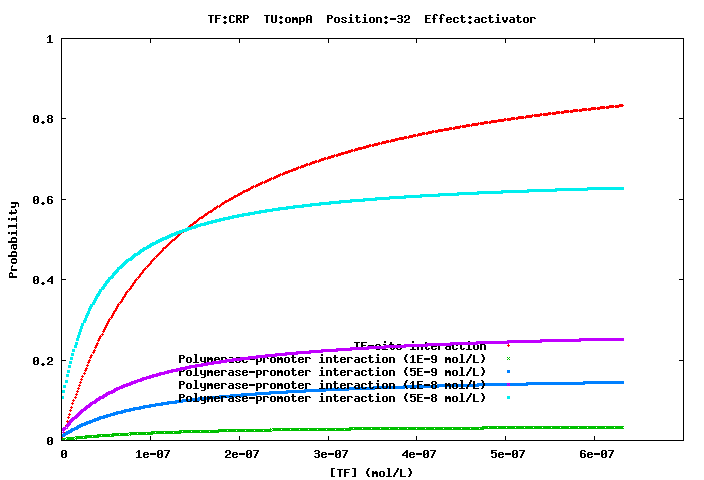

Supplement: Additional file 1 — Kinetic graphs of all E. coli simple promoters obtained as described in the Methods section. This file can be opened with tar. [file 1471-2199-10-92-S1.zip › sm1/CRP_atatgcctgaCGGAGTTCACACTTGTAAGTTTtcaactacgt_-32.pwm.plot.gif]

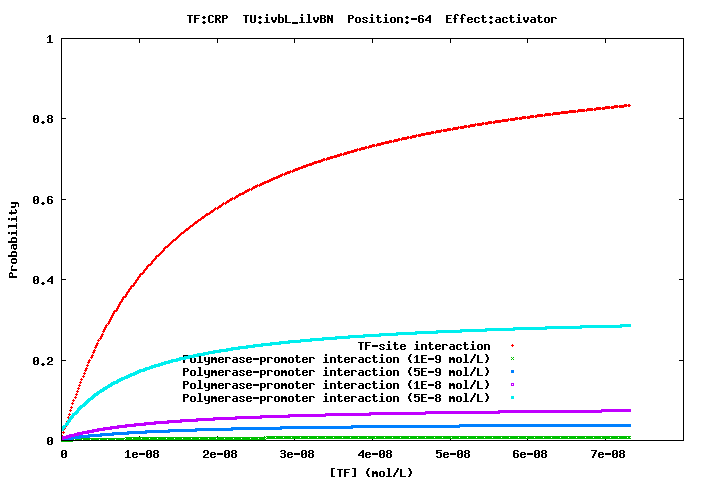

Supplement: Additional file 1 — Kinetic graphs of all E. coli simple promoters obtained as described in the Methods section. This file can be opened with tar. [file 1471-2199-10-92-S1.zip › sm1/CRP_attcagtacaAAACGTGATCAACCCCTCAATTttcccttgct_-64.pwm.plot.gif]

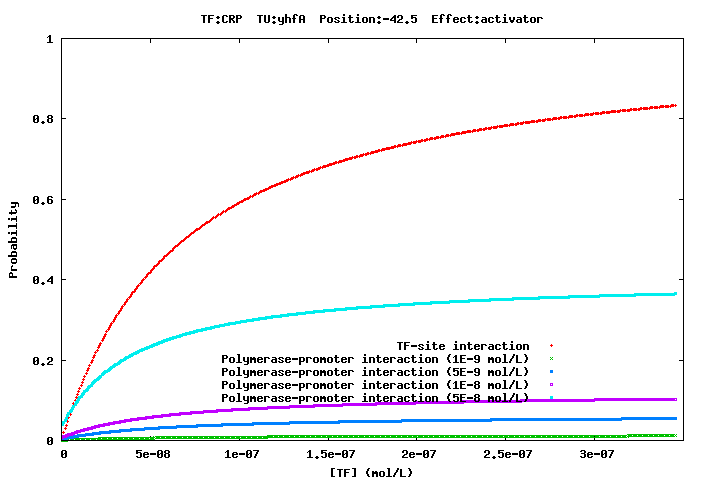

Supplement: Additional file 1 — Kinetic graphs of all E. coli simple promoters obtained as described in the Methods section. This file can be opened with tar. [file 1471-2199-10-92-S1.zip › sm1/CRP_actgcacggtAATGTGACGTCCTTTGCATACAtgcagtacat_-42.5.pwm.plot.gif]

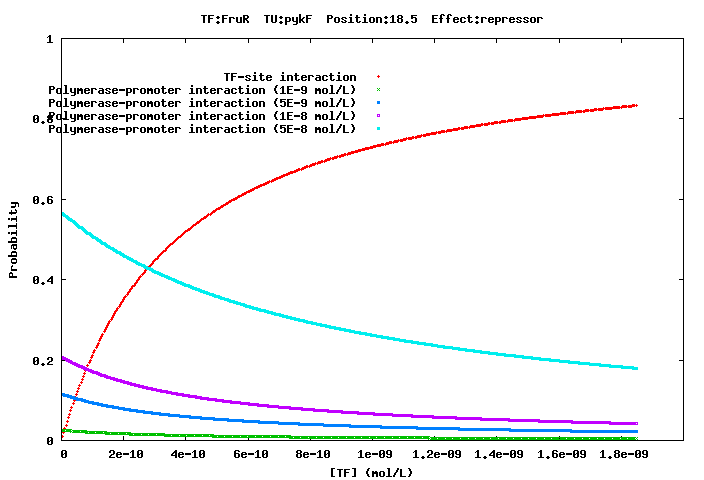

Supplement: Additional file 1 — Kinetic graphs of all E. coli simple promoters obtained as described in the Methods section. This file can be opened with tar. [file 1471-2199-10-92-S1.zip › sm1/FruR_gccaattgacTCTTGAATGGTTTCAGCActttggactg_18.5.pwm.plot.gif]

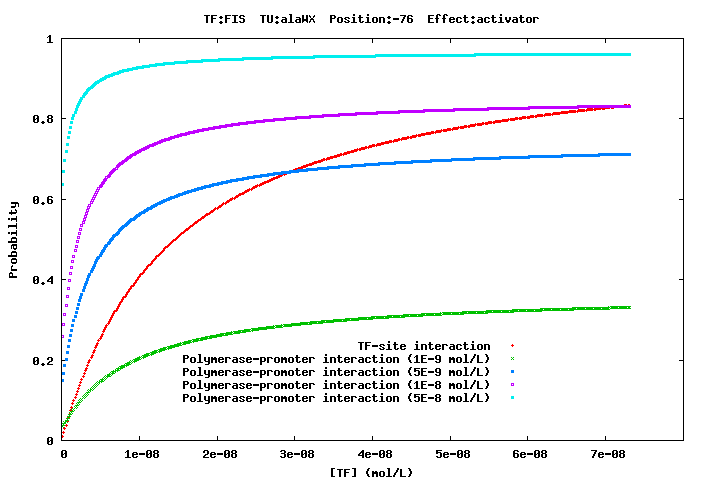

Supplement: Additional file 1 — Kinetic graphs of all E. coli simple promoters obtained as described in the Methods section. This file can be opened with tar. [file 1471-2199-10-92-S1.zip › sm1/FIS_attgttcattTGCCTGATTTTTCAGcgattgaaac_-76.pwm.plot.gif]

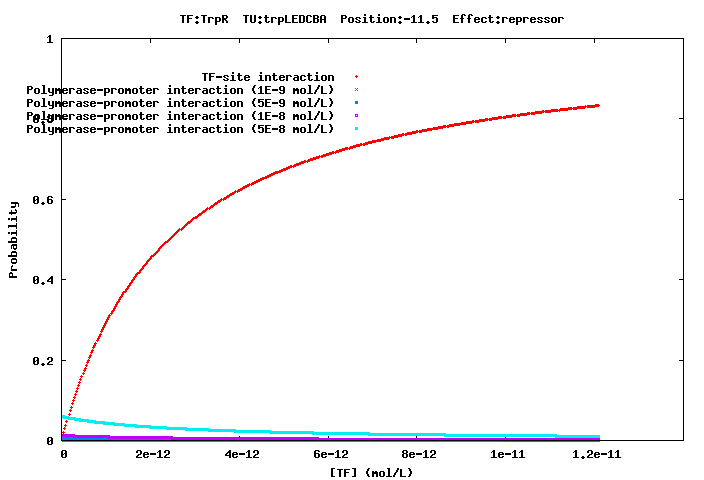

Supplement: Additional file 1 — Kinetic graphs of all E. coli simple promoters obtained as described in the Methods section. This file can be opened with tar. [file 1471-2199-10-92-S1.zip › sm1/TrpR_attaatcatcGAACTAGTTAACTAGTACgcaagttcac_-11.5.pwm.plot.gif]

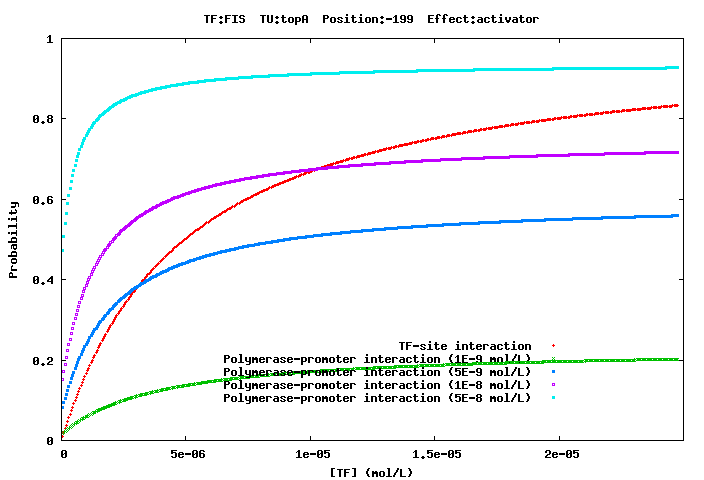

Supplement: Additional file 1 — Kinetic graphs of all E. coli simple promoters obtained as described in the Methods section. This file can be opened with tar. [file 1471-2199-10-92-S1.zip › sm1/FIS_ggattttgcaTGCTAATAAAGTTGCgtatcggatt_-199.pwm.plot.gif]

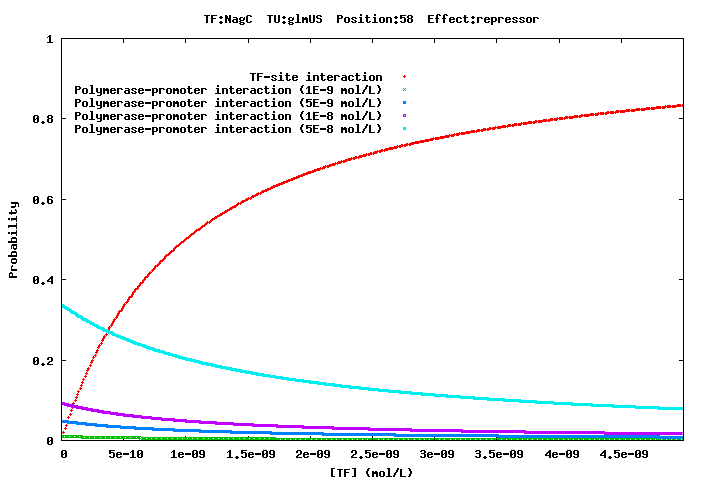

Supplement: Additional file 1 — Kinetic graphs of all E. coli simple promoters obtained as described in the Methods section. This file can be opened with tar. [file 1471-2199-10-92-S1.zip › sm1/NagC_ttatcctctgTCCATTTCACGATGAAAAAAATGtagttttttc_58.pwm.plot.gif]

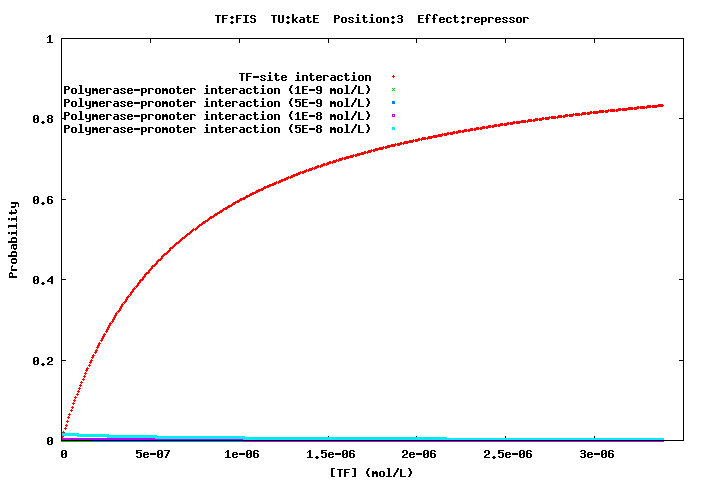

Supplement: Additional file 1 — Kinetic graphs of all E. coli simple promoters obtained as described in the Methods section. This file can be opened with tar. [file 1471-2199-10-92-S1.zip › sm1/FIS_tctatagttaGAGAGTTTTTTGACCaaaacagcgg_3.pwm.plot.gif]

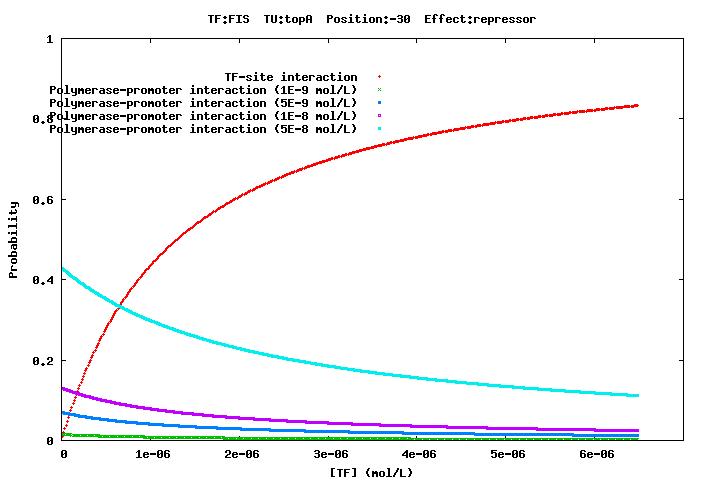

Supplement: Additional file 1 — Kinetic graphs of all E. coli simple promoters obtained as described in the Methods section. This file can be opened with tar. [file 1471-2199-10-92-S1.zip › sm1/FIS_cacgacaaggGGTTGATATCCGCAGagagcgagtc_-30.pwm.plot.gif]

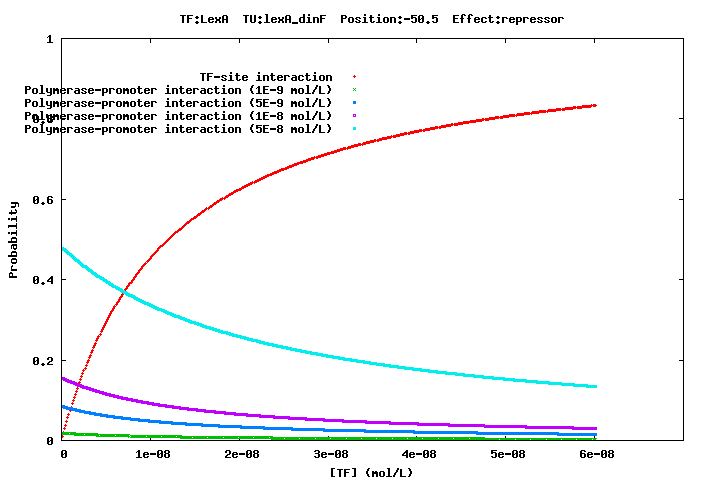

Supplement: Additional file 1 — Kinetic graphs of all E. coli simple promoters obtained as described in the Methods section. This file can be opened with tar. [file 1471-2199-10-92-S1.zip › sm1/LexA_ttcgataaatCTCTGGTTTATTGTGCAGTTtatggttcca_-50.5.pwm.plot.gif]

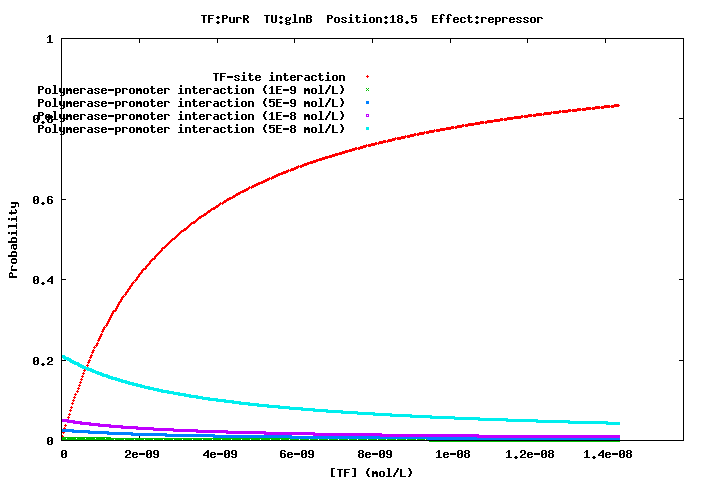

Supplement: Additional file 1 — Kinetic graphs of all E. coli simple promoters obtained as described in the Methods section. This file can be opened with tar. [file 1471-2199-10-92-S1.zip › sm1/PurR_gacacgagctGGATGCAAACGATTTCaaggaatgaa_18.5.pwm.plot.gif]

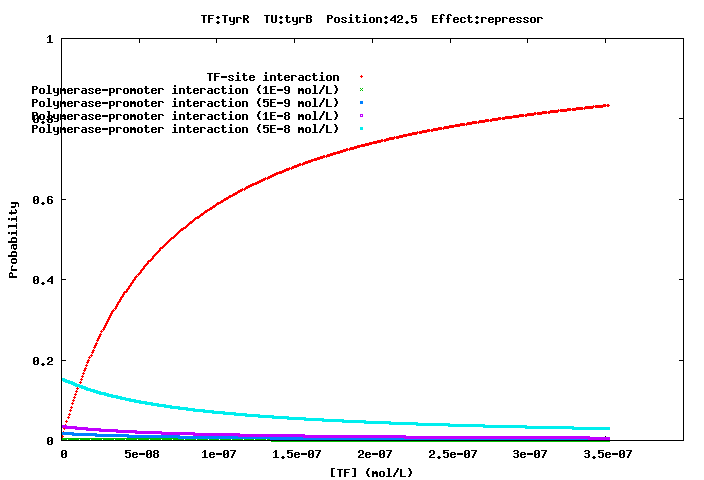

Supplement: Additional file 1 — Kinetic graphs of all E. coli simple promoters obtained as described in the Methods section. This file can be opened with tar. [file 1471-2199-10-92-S1.zip › sm1/TyrR_aaccatcgcgTGTTTCAAAAAGTTGACGcctacgctgg_42.5.pwm.plot.gif]

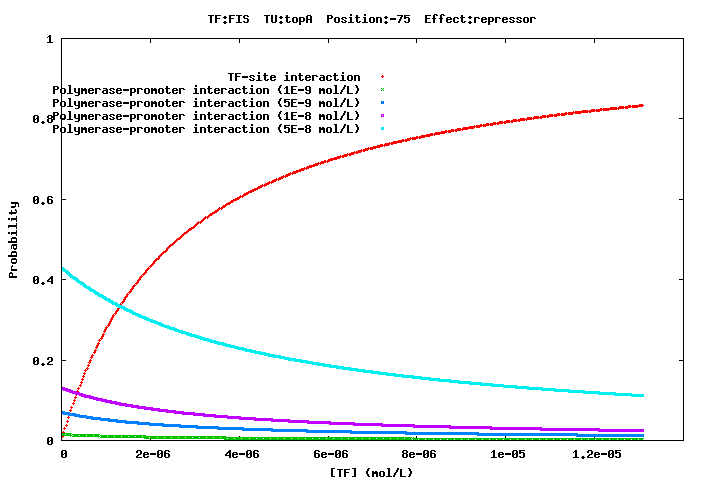

Supplement: Additional file 1 — Kinetic graphs of all E. coli simple promoters obtained as described in the Methods section. This file can be opened with tar. [file 1471-2199-10-92-S1.zip › sm1/FIS_ctggtggcaaGAGCGCCTTACTGGCaactttggat_-75.pwm.plot.gif]

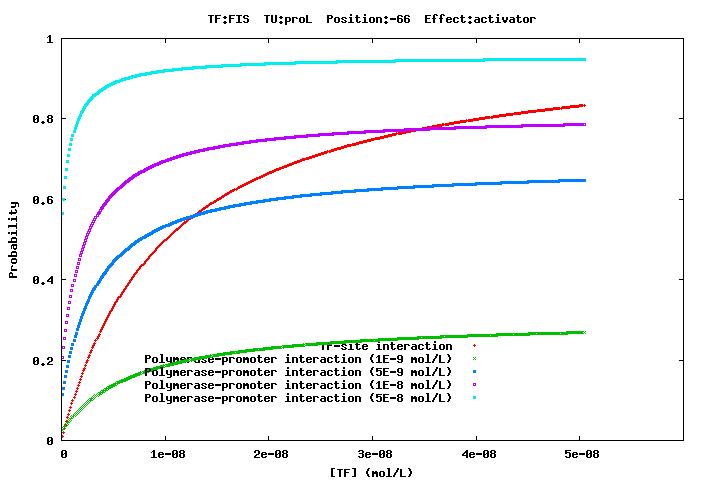

Supplement: Additional file 1 — Kinetic graphs of all E. coli simple promoters obtained as described in the Methods section. This file can be opened with tar. [file 1471-2199-10-92-S1.zip › sm1/FIS_tatcgctaacTGATTAATTATAAATcagttagcga_-66.pwm.plot.gif]

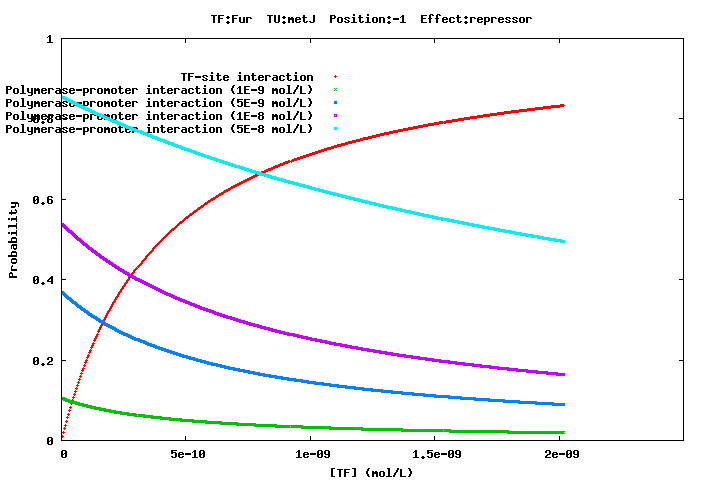

Supplement: Additional file 1 — Kinetic graphs of all E. coli simple promoters obtained as described in the Methods section. This file can be opened with tar. [file 1471-2199-10-92-S1.zip › sm1/Fur_tagccgttaaAATTATATGCATTATCACGccgacaggtg_-1.pwm.plot.gif]

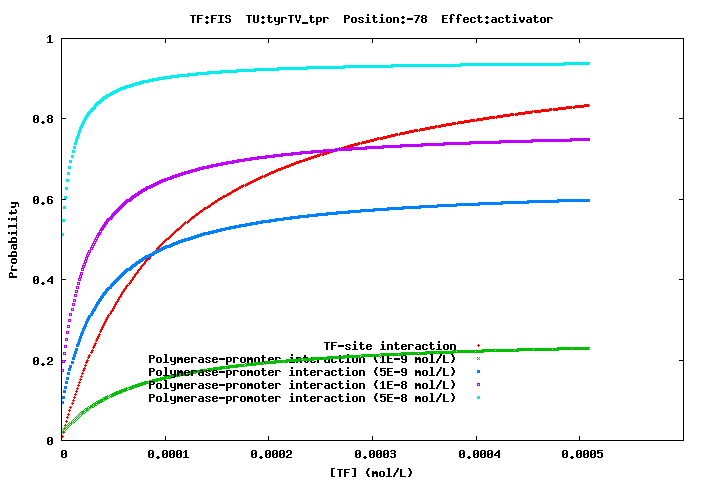

Supplement: Additional file 1 — Kinetic graphs of all E. coli simple promoters obtained as described in the Methods section. This file can be opened with tar. [file 1471-2199-10-92-S1.zip › sm1/FIS_tacctttaatCCGTTACGGATGAAAattacgcaac_-78.pwm.plot.gif]

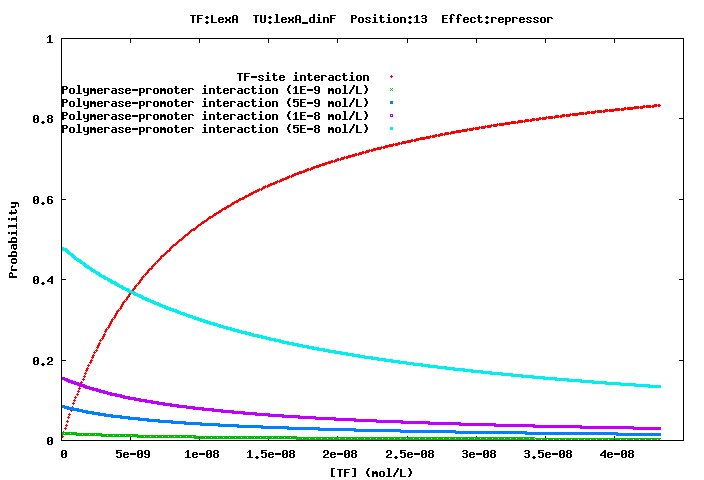

Supplement: Additional file 1 — Kinetic graphs of all E. coli simple promoters obtained as described in the Methods section. This file can be opened with tar. [file 1471-2199-10-92-S1.zip › sm1/LexA_ctcacagcatAACTGTATATACACCCAGGGggcggaatga_13.pwm.plot.gif]

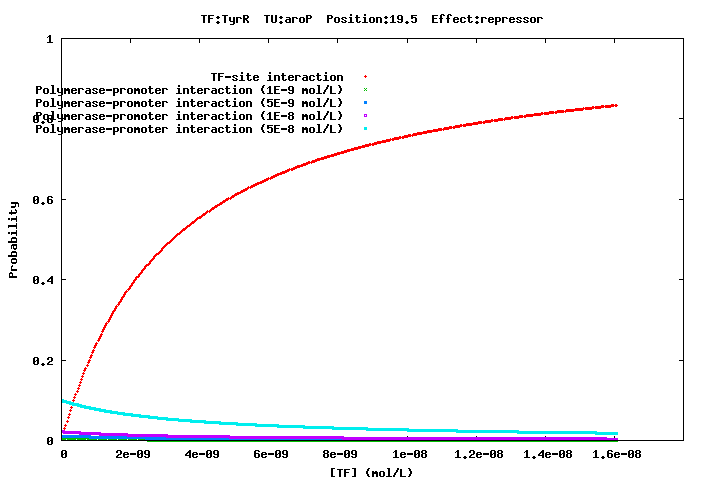

Supplement: Additional file 1 — Kinetic graphs of all E. coli simple promoters obtained as described in the Methods section. This file can be opened with tar. [file 1471-2199-10-92-S1.zip › sm1/TyrR_aacttctttgATGTAAACAAATTAATACaacaaacgga_19.5.pwm.plot.gif]

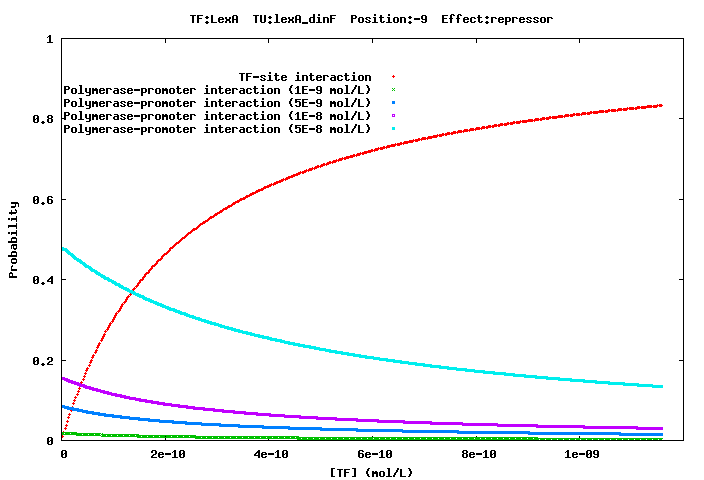

Supplement: Additional file 1 — Kinetic graphs of all E. coli simple promoters obtained as described in the Methods section. This file can be opened with tar. [file 1471-2199-10-92-S1.zip › sm1/LexA_aatcgcctttTGCTGTATATACTCACAGCAtaactgtata_-9.pwm.plot.gif]

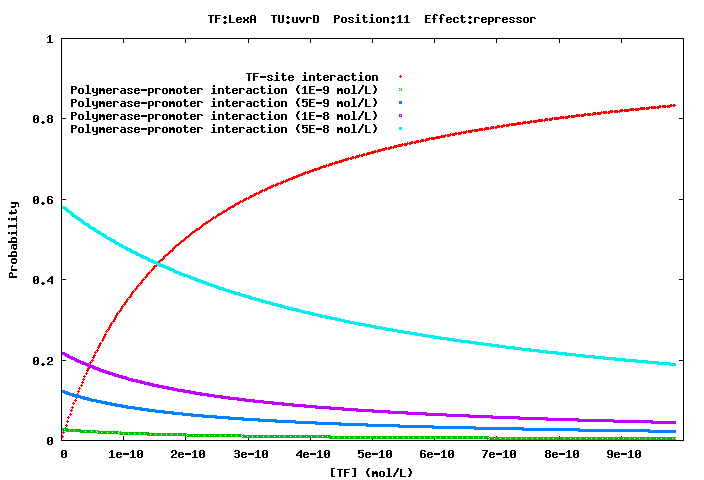

Supplement: Additional file 1 — Kinetic graphs of all E. coli simple promoters obtained as described in the Methods section. This file can be opened with tar. [file 1471-2199-10-92-S1.zip › sm1/LexA_taatcagcaaATCTGTATATATACCCAGCTttttggcgga_11.pwm.plot.gif]

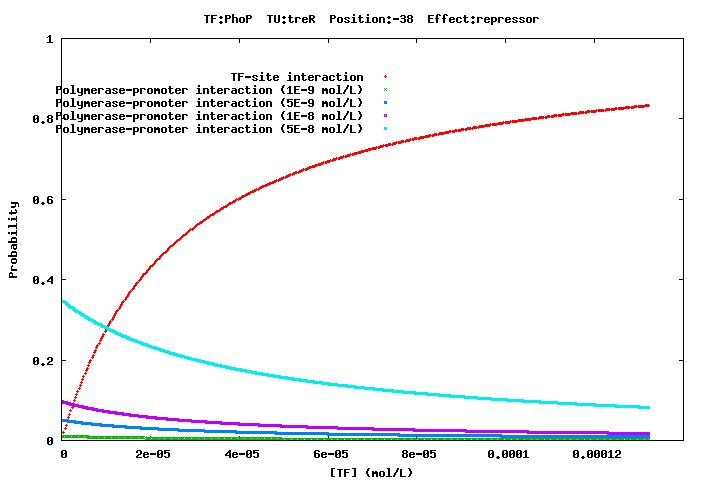

Supplement: Additional file 1 — Kinetic graphs of all E. coli simple promoters obtained as described in the Methods section. This file can be opened with tar. [file 1471-2199-10-92-S1.zip › sm1/PhoP_tgctgacaacTAAACCAACGATAAACCagactttacc_-38.pwm.plot.gif]

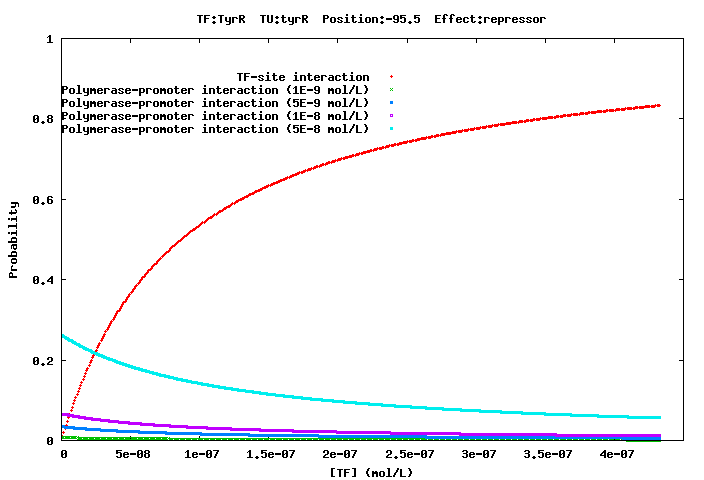

Supplement: Additional file 1 — Kinetic graphs of all E. coli simple promoters obtained as described in the Methods section. This file can be opened with tar. [file 1471-2199-10-92-S1.zip › sm1/TyrR_tatcgggtgcTGACCGGATATCTTTACGccgaagtgcc_-95.5.pwm.plot.gif]

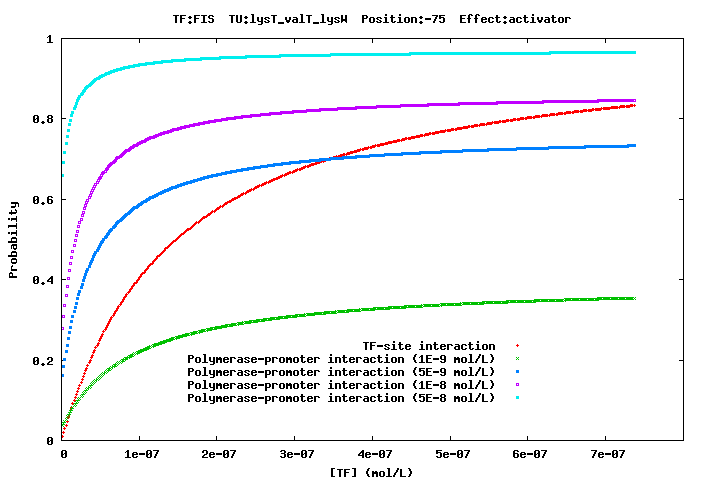

Supplement: Additional file 1 — Kinetic graphs of all E. coli simple promoters obtained as described in the Methods section. This file can be opened with tar. [file 1471-2199-10-92-S1.zip › sm1/FIS_ctggtcgtgtCGTGCGAATCATAAGcagttgagtg_-75.pwm.plot.gif]

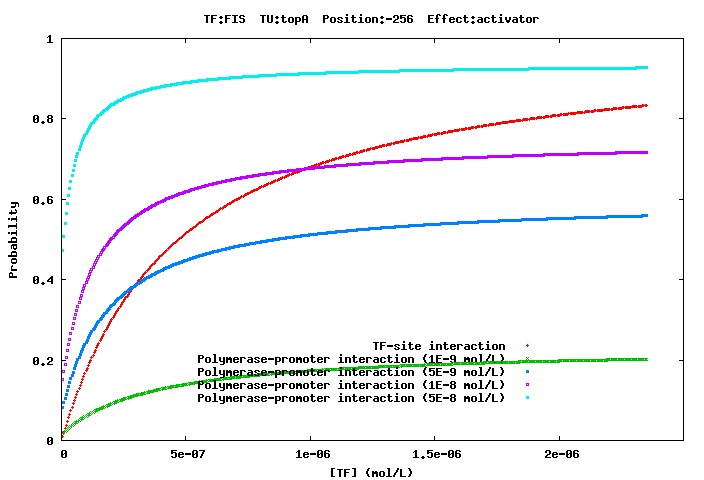

Supplement: Additional file 1 — Kinetic graphs of all E. coli simple promoters obtained as described in the Methods section. This file can be opened with tar. [file 1471-2199-10-92-S1.zip › sm1/FIS_catggcttgcAGACAAATATACCACgctggtggca_-256.pwm.plot.gif]

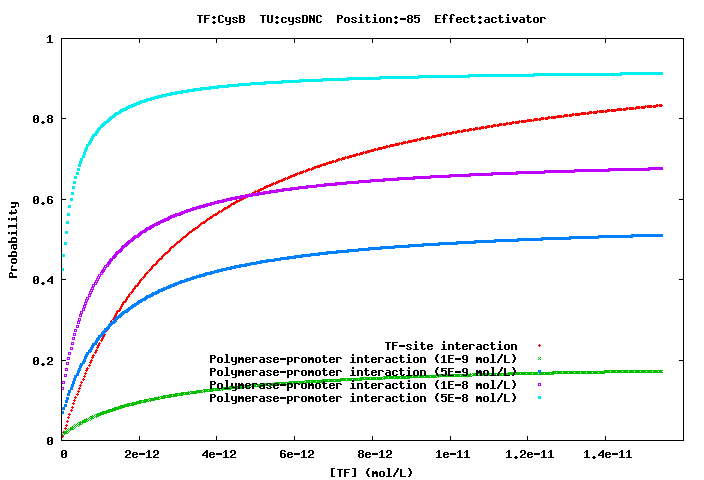

Supplement: Additional file 1 — Kinetic graphs of all E. coli simple promoters obtained as described in the Methods section. This file can be opened with tar. [file 1471-2199-10-92-S1.zip › sm1/CysB_cggtgccttaAGCACTTTTTGATATTAGCTTTGCCAAATCGTTATTCCGTTAaggaactact_-85.pwm.plot.gif]

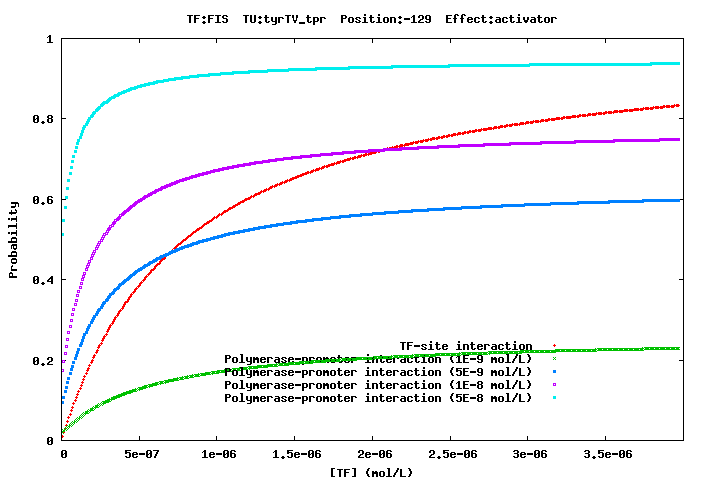

Supplement: Additional file 1 — Kinetic graphs of all E. coli simple promoters obtained as described in the Methods section. This file can be opened with tar. [file 1471-2199-10-92-S1.zip › sm1/FIS_gtttacggtaATCGAACGATTATTCtttaatcgcc_-129.pwm.plot.gif]

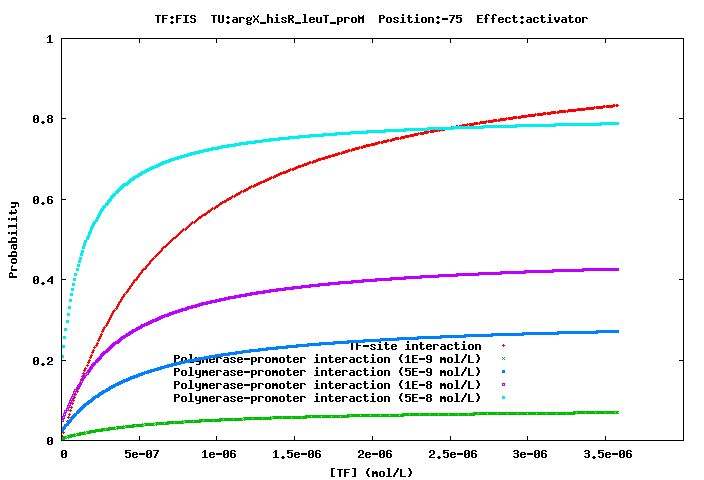

Supplement: Additional file 1 — Kinetic graphs of all E. coli simple promoters obtained as described in the Methods section. This file can be opened with tar. [file 1471-2199-10-92-S1.zip › sm1/FIS_agcagcaaaaCGCACAAACCGTAACcaaacgcgca_-75.pwm.plot.gif]

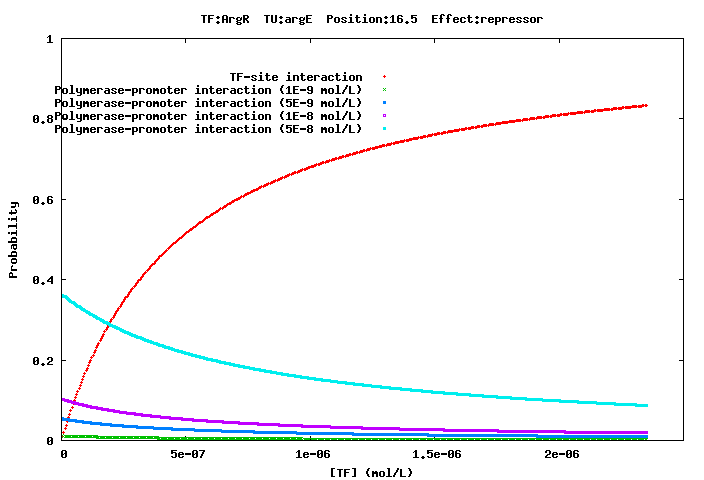

Supplement: Additional file 1 — Kinetic graphs of all E. coli simple promoters obtained as described in the Methods section. This file can be opened with tar. [file 1471-2199-10-92-S1.zip › sm1/ArgR_ttcataaataCTGCATGAATATTGATACtatcatgacc_16.5.pwm.plot.gif]

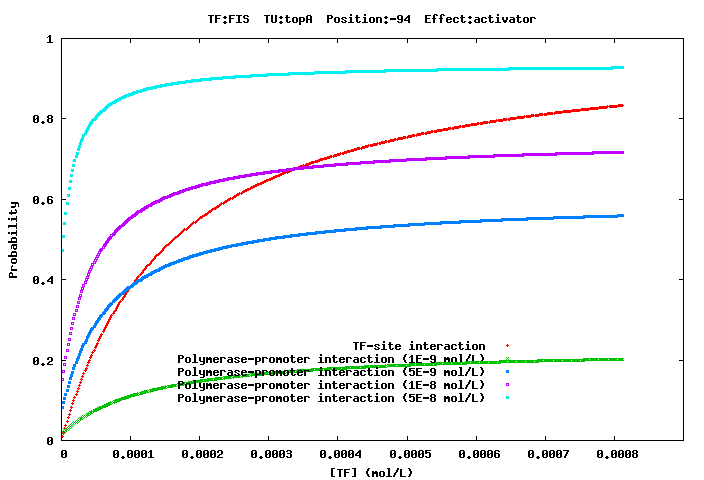

Supplement: Additional file 1 — Kinetic graphs of all E. coli simple promoters obtained as described in the Methods section. This file can be opened with tar. [file 1471-2199-10-92-S1.zip › sm1/FIS_cgtgctatagCGCCTGTAGGCCAAGacctgttaac_-94.pwm.plot.gif]

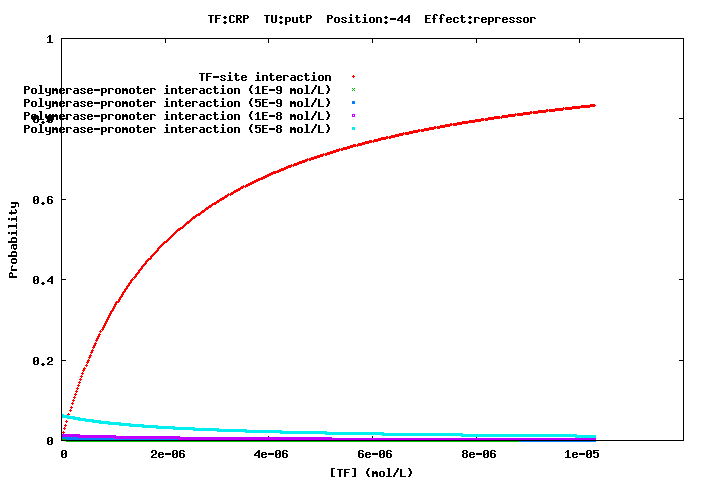

Supplement: Additional file 1 — Kinetic graphs of all E. coli simple promoters obtained as described in the Methods section. This file can be opened with tar. [file 1471-2199-10-92-S1.zip › sm1/CRP_aaaatttaatGTAAATGGTGTGTTAAATCGATtgtgaataac_-44.pwm.plot.gif]

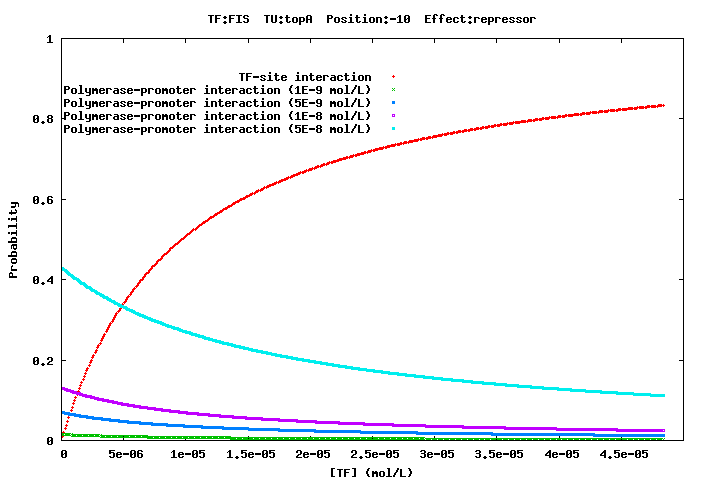

Supplement: Additional file 1 — Kinetic graphs of all E. coli simple promoters obtained as described in the Methods section. This file can be opened with tar. [file 1471-2199-10-92-S1.zip › sm1/FIS_cgcagagagcGAGTCCATATCGGTAactcgttgcc_-10.pwm.plot.gif]

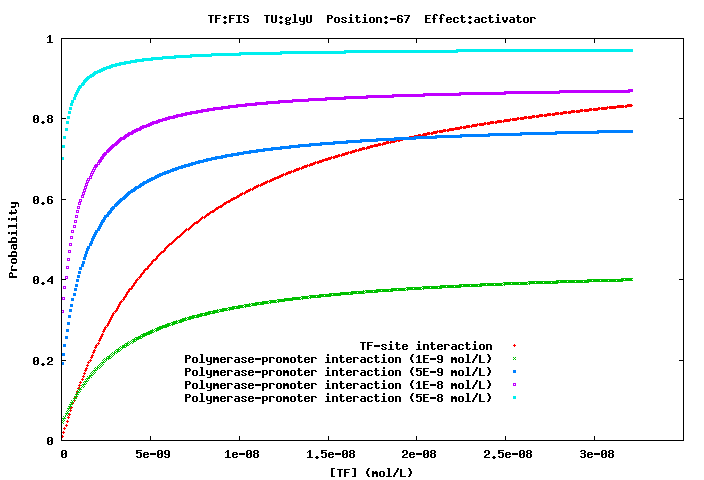

Supplement: Additional file 1 — Kinetic graphs of all E. coli simple promoters obtained as described in the Methods section. This file can be opened with tar. [file 1471-2199-10-92-S1.zip › sm1/FIS_gccaaaatgcTGATGGCGAATTAATcagcagtcag_-67.pwm.plot.gif]

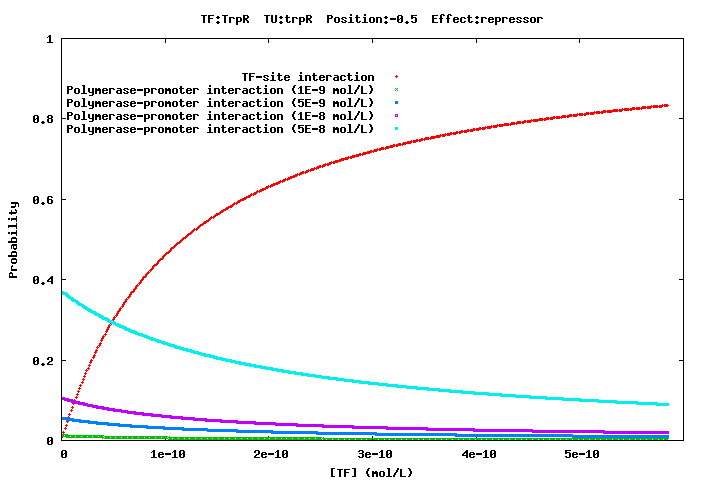

Supplement: Additional file 1 — Kinetic graphs of all E. coli simple promoters obtained as described in the Methods section. This file can be opened with tar. [file 1471-2199-10-92-S1.zip › sm1/TrpR_atatgctatcGTACTCTTTAGCGAGTACaaccggggga_-0.5.pwm.plot.gif]

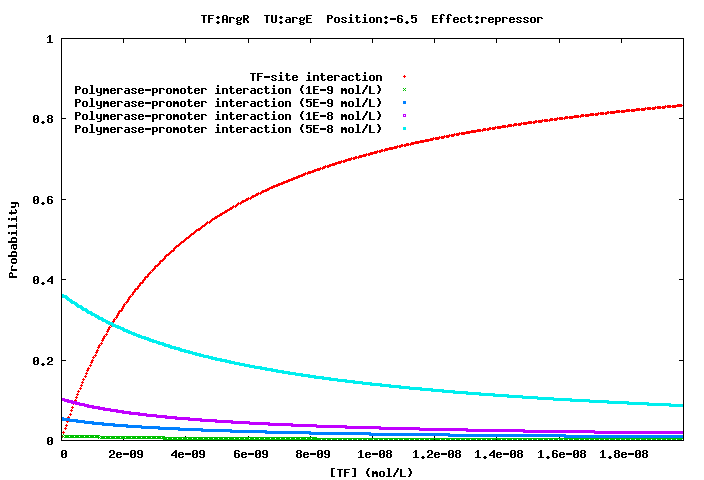

Supplement: Additional file 1 — Kinetic graphs of all E. coli simple promoters obtained as described in the Methods section. This file can be opened with tar. [file 1471-2199-10-92-S1.zip › sm1/ArgR_gctcaacgttAGTGTATTTTTATTCATAaatactgcat_-6.5.pwm.plot.gif]

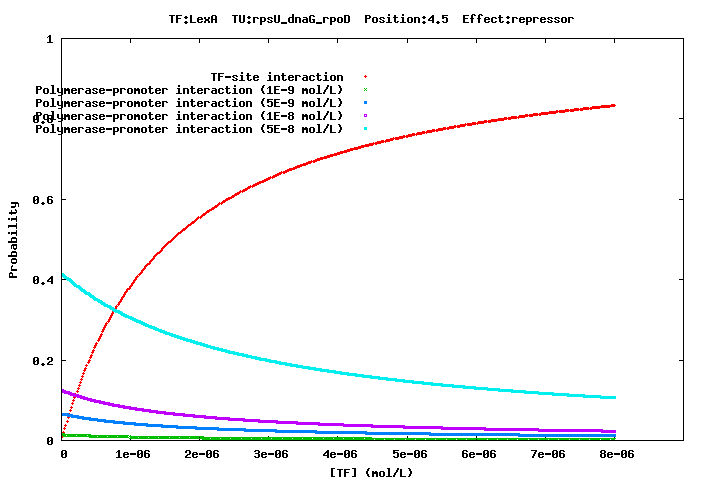

Supplement: Additional file 1 — Kinetic graphs of all E. coli simple promoters obtained as described in the Methods section. This file can be opened with tar. [file 1471-2199-10-92-S1.zip › sm1/LexA_attttgaaatAAGCTGGCGTTGATGCCAGCggcaaaccga_4.5.pwm.plot.gif]

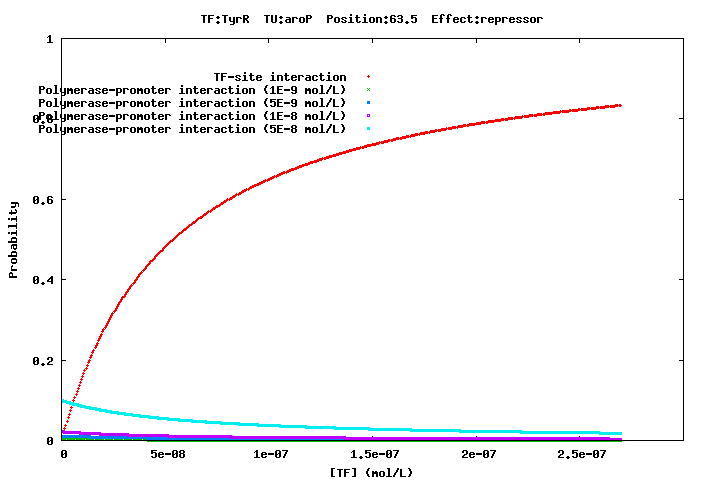

Supplement: Additional file 1 — Kinetic graphs of all E. coli simple promoters obtained as described in the Methods section. This file can be opened with tar. [file 1471-2199-10-92-S1.zip › sm1/TyrR_aatacaacaaACGGAATTGCAAACTTACacacgcatca_63.5.pwm.plot.gif]

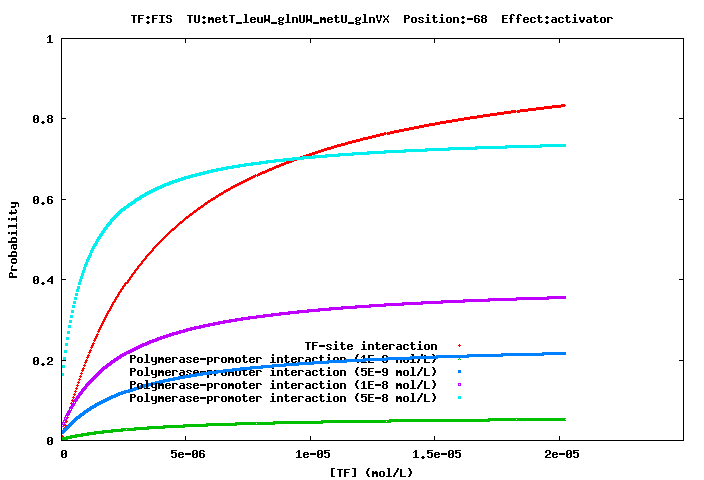

Supplement: Additional file 1 — Kinetic graphs of all E. coli simple promoters obtained as described in the Methods section. This file can be opened with tar. [file 1471-2199-10-92-S1.zip › sm1/FIS_acctaaccaaACAGTCACTTTCGAGcaattttcct_-68.pwm.plot.gif]

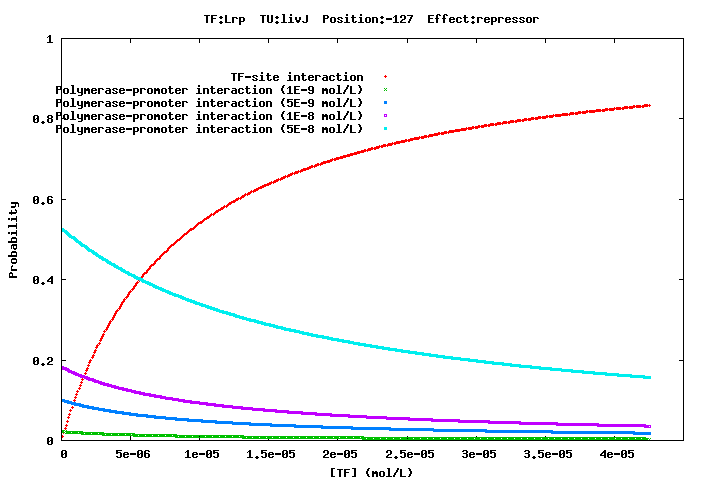

Supplement: Additional file 1 — Kinetic graphs of all E. coli simple promoters obtained as described in the Methods section. This file can be opened with tar. [file 1471-2199-10-92-S1.zip › sm1/Lrp_tggatgagagTCCGGGGTTTTTgttttttggg_-127.pwm.plot.gif]

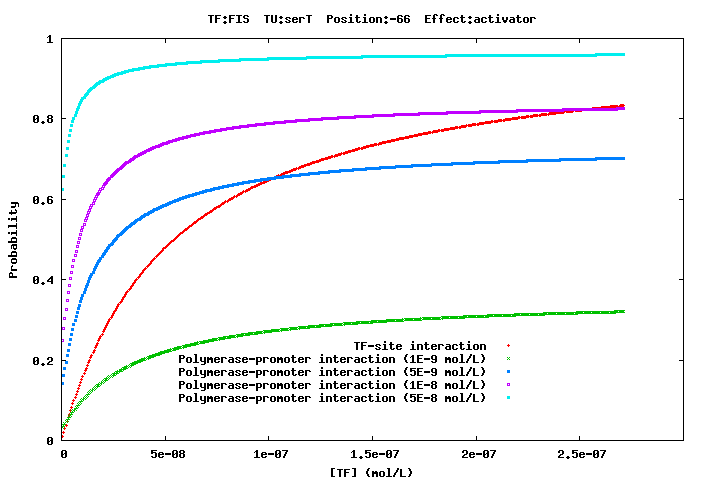

Supplement: Additional file 1 — Kinetic graphs of all E. coli simple promoters obtained as described in the Methods section. This file can be opened with tar. [file 1471-2199-10-92-S1.zip › sm1/FIS_aatgcactttTGGCTGTTTTTCAGGcaaacaaaca_-66.pwm.plot.gif]

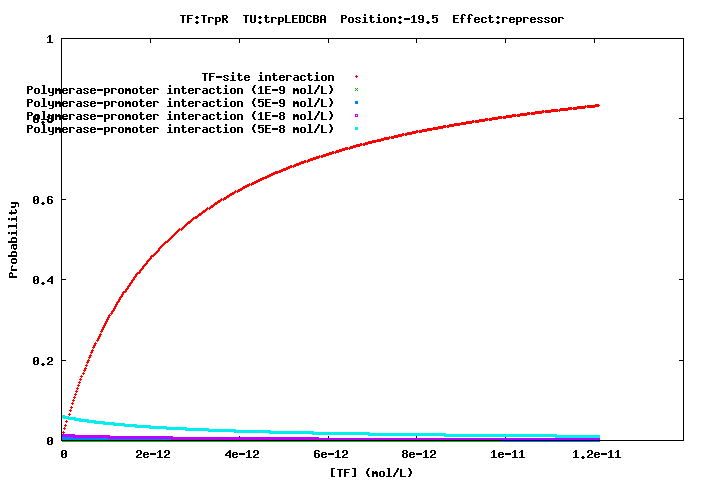

Supplement: Additional file 1 — Kinetic graphs of all E. coli simple promoters obtained as described in the Methods section. This file can be opened with tar. [file 1471-2199-10-92-S1.zip › sm1/TrpR_tgttgacaatTAATCATCGAACTAGTTAactagtacgc_-19.5.pwm.plot.gif]

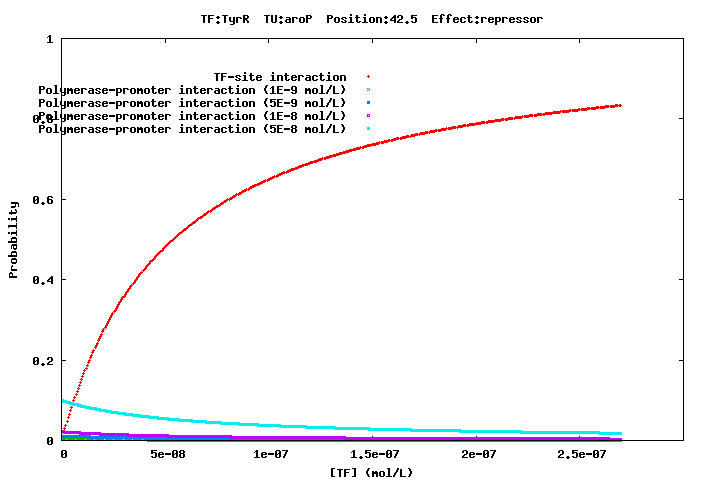

Supplement: Additional file 1 — Kinetic graphs of all E. coli simple promoters obtained as described in the Methods section. This file can be opened with tar. [file 1471-2199-10-92-S1.zip › sm1/TyrR_aatacaacaaACGGAATTGCAAACTTACacacgcatca_42.5.pwm.plot.gif]

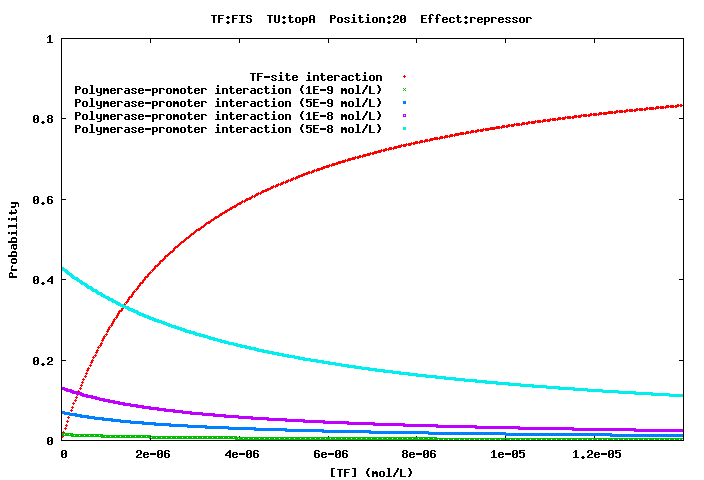

Supplement: Additional file 1 — Kinetic graphs of all E. coli simple promoters obtained as described in the Methods section. This file can be opened with tar. [file 1471-2199-10-92-S1.zip › sm1/FIS_gttgccagtgGAAGGTTTATCAACGtgcgacgcat_20.pwm.plot.gif]

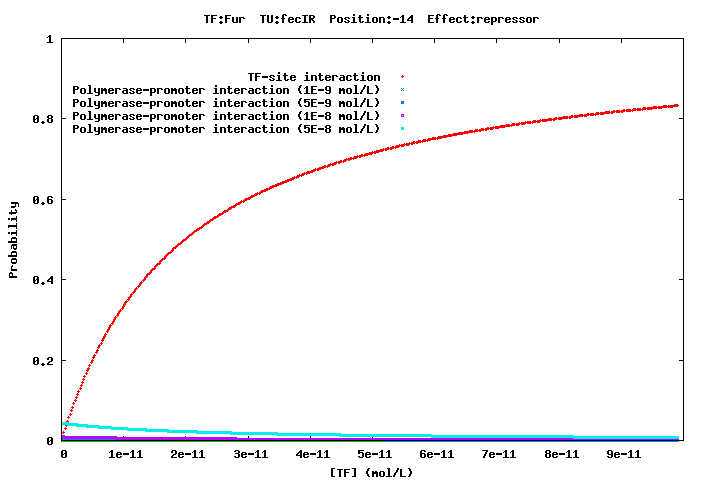

Supplement: Additional file 1 — Kinetic graphs of all E. coli simple promoters obtained as described in the Methods section. This file can be opened with tar. [file 1471-2199-10-92-S1.zip › sm1/Fur_acaattttatTTCCAATTGTAATGATAACcattctcata_-14.pwm.plot.gif]

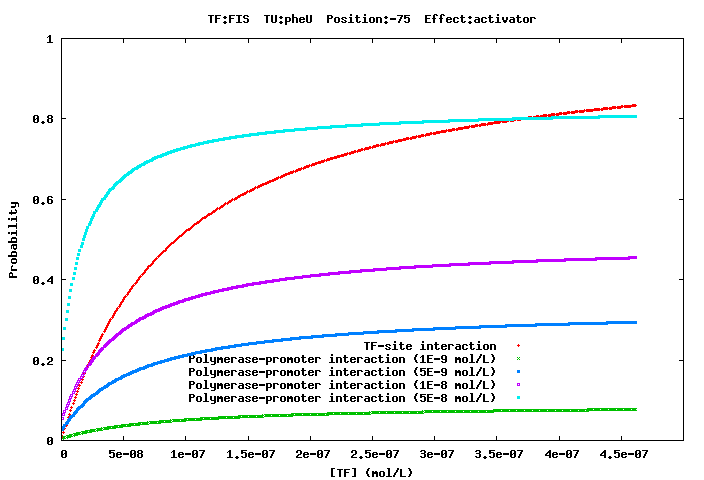

Supplement: Additional file 1 — Kinetic graphs of all E. coli simple promoters obtained as described in the Methods section. This file can be opened with tar. [file 1471-2199-10-92-S1.zip › sm1/FIS_aagaagcgatTTGCCGCAATCTTAAgcagttgaat_-75.pwm.plot.gif]
