# Supplementary material for: From sequence to dynamics: the effects of transcription factor and polymerase concentration changes on activated and repressed promoters
Source: BMC Mol Biol. 2009 Sep 22;10:92. doi: 10.1186/1471-2199-10-92 (PMC2761915; doi:10.1186/1471-2199-10-92)
Supplement: Additional file 2 — Boundaries imposed by the reduction of RNAP concentration to one tenth of Kp on the sigmoid dependence of promoter occupancy probability on Kd for A) activator sites and B) repressor sites. This file can be open with PDF viewer. [file 1471-2199-10-92-S2.pdf]

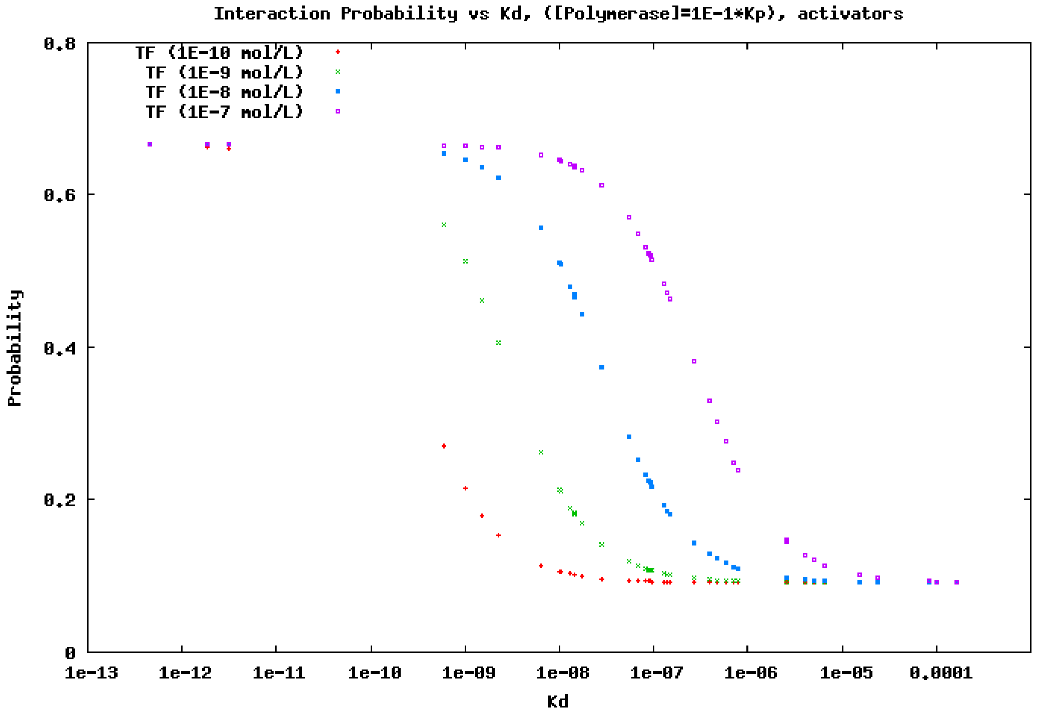

A

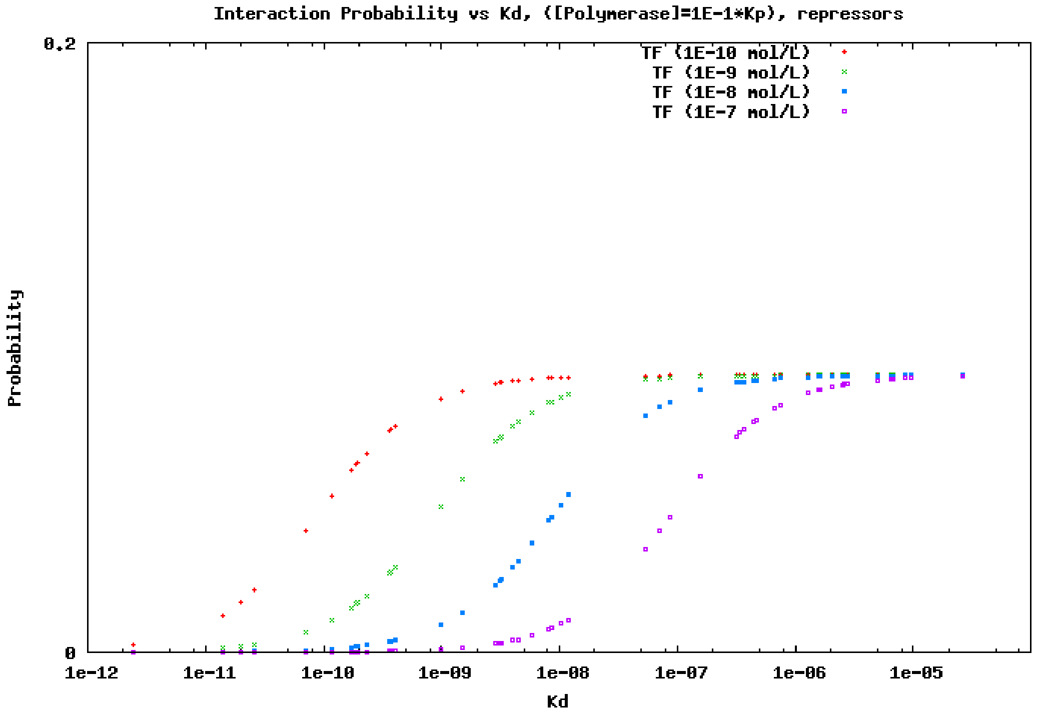

B

Figure add_file2: Probability of polymerase-promoter interaction computed for: **A**, activator sites, at polymerase concentration equal to one tenth of the Kp of promoters, at four TF concentrations; **B**, repressor sites at polymerase concentration equal to one tenth of the Kp of promoters, at four TF concentrations
