# Supplementary material for: From sequence to dynamics: the effects of transcription factor and polymerase concentration changes on activated and repressed promoters
Source: BMC Mol Biol. 2009 Sep 22;10:92. doi: 10.1186/1471-2199-10-92 (PMC2761915; doi:10.1186/1471-2199-10-92)
Supplement: Additional file 3 — Results of confronting data produced by our model with microarray data from FNR-activated TUs in three experimental conditions. This file can be open with PDF viewer. [file 1471-2199-10-92-S3.pdf]

Additional file 3: Results of confronting data produced by our model with microarray data from FNR-activated TUs in three experimental conditions

We obtained microarray data for FNR-activated TUs in three experimental conditions from Constantinidou *et al.* (2006)[1]. Specifically, we extracted the relative abundance of FNR-activated TUs' mRNAS in *E. coli* wild type cells cultivated with oxygen (O_2), nitrate (NO_3^-) or nitrite (NO_2^-), with respect to cells that had been grown in complete anaerobiosis ($Ø_2$). These ratios may be readily used to compute the activated FNR concentration in those three situations. To do so, we isolated the [TF] term in equations I, II and III combined under the assumption that the activated FNR concentration in the three tested conditions is negligible compared to the one in the reference culture. (The polymerase concentration used to compute [TF] was 1E–09).

The TF concentration calculated for each promoter-site complex for each experimental condition (compared to the reference culture) was used to compute (through the theoretical model represented by equations I, II and III combined) the ratio of the probability of Polymerase-promoter binding for all other promoter-site complexes under the same simulated condition to the reference culture. That means that, for example, the TF concentration calculated from the promoter-site complex of the *nikABCDER* TU for the $O_2/Ø_2$ ratio was used to compute the Polymerase-promoter binding probability ratio corresponding to $O_2/Ø_2$ for all other TUs.

We then calculated 2 quotients that represented the trend of activation of each TU of cells cultivated with nitrate (or nitrite) with respect to those cultivated under aerobiosis. (Since they are quotients of ratios with equal denominators we represented them abbreviatedly by NO_3^-/ O_2 and NO_2^-/ O_2). We calculated these quotients both for experimentally measured expression ratios and theoretically computed Polymerase-promoter binding probability ratios. Since these ratios should be equivalent under our model, we expected to observe similar quotients — i.e. similar activation trends of TUs under diverse conditions — in experimental data and equivalent theoretical calculations.

The results of this comparison are shown in the Table at the end of this file, which presents experimental and theoretical quotients calculated for each TU from another predictor TU (TU employed to compute the activated FNR concentration) and the ratio of theoretical to experimental quotient. To analyze the results, we considered three levels of consistency between theoretically predicted trends and experimental ones. First, the ratio of theoretical to experimental quotient is between 0.5 and 2; second, the ratio is between 0.25 and 4; third, the ratio is between 0.1 and 10. We found that 65% of the predicted trends fall within the first boundaries; the disagreement of 81% of them with experimental ratios is no more than four-fold; and 93% of them are of the same order of magnitude than experimental ratios. We think that these levels of consistency between

[1] To carry out this confrontation with experimental data, we used all FNR-activated TUs with a calculated promoter score regardless of whether or not they were under the regulation of other TF. The reasoning here is that we were looking at the conservation of activation trends in the comparison of the three experimental conditions, rather than exact reproduction of experimental data by the theoretical model, and co-activation may make an important contribution to such trends.

theoretical and experimental trends is fairly good, considering the limitations of our model, the data available to carry on the computations, and the noisy nature of microarray data, as well as the difficulty in reproducing them.

Reference:

Constantinidou C, Hobman JL, Griffiths L, Patel MD, Penn CW, Cole JA, Overton TW: **A Reassessment of the FNR Regulon and Transcriptomic Analysis of the Effects of Nitrate, Nitrite, NarXL, and NarQP as *Escherichia coli* K12 Adapts from Aerobic to Anaerobic Growth.** *JBC* 2006, **281:**4802-4815

		Experimental		Theoretical		Theo/Exp	
Predictor TU	TU calculated	NO_3^-/O_2	NO_2^-/O_2	NO_3^-/O_2	NO_2^-/O_2	NO_3^-/O_2	NO_2^-/O_2
aspA_dcuA	hypBCDE	2.95	3.35	0.11	1.41	0.04	0.42
aspA_dcuA	hypBCDE	2.95	3.35	0.11	1.41	0.04	0.42
aspA_dcuA	moaABCDE	3.04	1.94	0.14	1.39	0.05	0.72
aspA_dcuA	yhjA	3	5.91	0.15	1.39	0.05	0.24
nikABCDER	hypBCDE	2.95	3.35	0.19	0.44	0.06	0.13
nikABCDER	hypBCDE	2.95	3.35	0.19	0.44	0.06	0.13
nikABCDER	moaABCDE	3.04	1.94	0.2	0.45	0.07	0.23
nikABCDER	yhjA	3	5.91	0.21	0.45	0.07	0.08
aspA_dcuA	dcuB_fumB	1.5	4	0.12	1.4	0.08	0.35
aspA_dcuA	nirBDC_cysG	1.34	1.08	0.11	1.4	0.08	1.30
nikABCDER	dcuB_fumB	1.5	4	0.19	0.45	0.13	0.11
aspA_dcuA	focA_pflB	1.46	3.62	0.19	1.37	0.13	0.38
aspA_dcuA	focA_pflB	1.46	3.62	0.19	1.37	0.13	0.38
aspA_dcuA	ydhYVWXUT	1.64	2.21	0.22	1.36	0.13	0.62
aspA_dcuA	caiTABCDE	1.04	0.94	0.14	1.39	0.13	1.48
aspA_dcuA	caiTABCDE	1.04	0.94	0.14	1.39	0.13	1.48
nikABCDER	ydhYVWXUT	1.64	2.21	0.23	0.47	0.14	0.21
nikABCDER	nirBDC_cysG	1.34	1.08	0.19	0.44	0.14	0.41
nikABCDER	focA_pflB	1.46	3.62	0.22	0.46	0.15	0.13
nikABCDER	focA_pflB	1.46	3.62	0.22	0.46	0.15	0.13
nikABCDER	caiTABCDE	1.04	0.94	0.2	0.45	0.19	0.48
nikABCDER	caiTABCDE	1.04	0.94	0.2	0.45	0.19	0.48
nikABCDER	dcuC	1.31	2.25	0.31	0.52	0.24	0.23
nikABCDER	dcuC	1.31	2.25	0.31	0.52	0.24	0.23
nikABCDER	dcuC	1.31	2.25	0.31	0.52	0.24	0.23
tdcABCDEFG	moaABCDE	3.04	1.94	0.72	1.11	0.24	0.57
tdcABCDEFG	moaABCDE	3.04	1.94	0.72	1.11	0.24	0.57
tdcABCDEFG	yhjA	3	5.91	0.73	1.11	0.24	0.19
tdcABCDEFG	yhjA	3	5.91	0.73	1.11	0.24	0.19
tdcABCDEFG	hypBCDE	2.95	3.35	0.72	1.11	0.24	0.33
tdcABCDEFG	hypBCDE	2.95	3.35	0.72	1.11	0.24	0.33
tdcABCDEFG	hypBCDE	2.95	3.35	0.72	1.11	0.24	0.33
tdcABCDEFG	hypBCDE	2.95	3.35	0.72	1.11	0.24	0.33
aspA_dcuA	dcuC	1.31	2.25	0.38	1.28	0.29	0.57
aspA_dcuA	dcuC	1.31	2.25	0.38	1.28	0.29	0.57
aspA_dcuA	dcuC	1.31	2.25	0.38	1.28	0.29	0.57
nikABCDER	aer	0.93	0.34	0.27	0.5	0.29	1.47
aer	moaABCDE	3.04	1.94	0.93	0.29	0.31	0.15
aer	yhjA	3	5.91	0.93	0.29	0.31	0.05
aer	hypBCDE	2.95	3.35	0.93	0.28	0.32	0.08
aer	hypBCDE	2.95	3.35	0.93	0.28	0.32	0.08
caiTABCDE	moaABCDE	3.04	1.94	1.04	0.94	0.34	0.48
caiTABCDE	moaABCDE	3.04	1.94	1.04	0.94	0.34	0.48
aspA_dcuA	aer	0.93	0.34	0.32	1.31	0.34	3.85
caiTABCDE	yhjA	3	5.91	1.04	0.94	0.35	0.16
caiTABCDE	yhjA	3	5.91	1.04	0.94	0.35	0.16
caiTABCDE	hypBCDE	2.95	3.35	1.04	0.94	0.35	0.28

caiTABCDE	hypBCDE	2.95	3.35	1.04	0.94	0.35	0.28
caiTABCDE	hypBCDE	2.95	3.35	1.04	0.94	0.35	0.28
caiTABCDE	hypBCDE	2.95	3.35	1.04	0.94	0.35	0.28
nikABCDER	tdcABCDEFG	0.8	1.08	0.33	0.54	0.41	0.50
nikABCDER	tdcABCDEFG	0.8	1.08	0.33	0.54	0.41	0.50
dcuB_fumB	yhjA	3	5.91	1.25	2.51	0.42	0.42
dcuB_fumB	moaABCDE	3.04	1.94	1.32	2.93	0.43	1.51
nirBDC_cysG	moaABCDE	3.04	1.94	1.33	1.08	0.44	0.56
nirBDC_cysG	yhjA	3	5.91	1.33	1.08	0.44	0.18
tdcABCDEFG	ydhYVWXUT	1.64	2.21	0.74	1.1	0.45	0.50
tdcABCDEFG	ydhYVWXUT	1.64	2.21	0.74	1.1	0.45	0.50
nirBDC_cysG	hypBCDE	2.95	3.35	1.34	1.08	0.45	0.32
nirBDC_cysG	hypBCDE	2.95	3.35	1.34	1.08	0.45	0.32
tdcABCDEFG	dcuB_fumB	1.5	4	0.72	1.11	0.48	0.28
tdcABCDEFG	dcuB_fumB	1.5	4	0.72	1.11	0.48	0.28
focA_pflB	moaABCDE	3.04	1.94	1.53	4.05	0.50	2.09
focA_pflB	moaABCDE	3.04	1.94	1.53	4.05	0.50	2.09
focA_pflB	yhjA	3	5.91	1.51	3.93	0.50	0.66
focA_pflB	yhjA	3	5.91	1.51	3.93	0.50	0.66
tdcABCDEFG	focA_pflB	1.46	3.62	0.74	1.11	0.51	0.31
tdcABCDEFG	focA_pflB	1.46	3.62	0.74	1.11	0.51	0.31
tdcABCDEFG	focA_pflB	1.46	3.62	0.74	1.11	0.51	0.31
tdcABCDEFG	focA_pflB	1.46	3.62	0.74	1.11	0.51	0.31
aspA_dcuA	tdcABCDEFG	0.8	1.08	0.42	1.26	0.53	1.17
aspA_dcuA	tdcABCDEFG	0.8	1.08	0.42	1.26	0.53	1.17
focA_pflB	hypBCDE	2.95	3.35	1.58	4.31	0.54	1.29
focA_pflB	hypBCDE	2.95	3.35	1.58	4.31	0.54	1.29
focA_pflB	hypBCDE	2.95	3.35	1.58	4.31	0.54	1.29
focA_pflB	hypBCDE	2.95	3.35	1.58	4.31	0.54	1.29
tdcABCDEFG	nirBDC_cysG	1.34	1.08	0.72	1.11	0.54	1.03
tdcABCDEFG	nirBDC_cysG	1.34	1.08	0.72	1.11	0.54	1.03
dcuC	yhjA	3	5.91	1.66	3.66	0.55	0.62
dcuC	yhjA	3	5.91	1.66	3.66	0.55	0.62
dcuC	yhjA	3	5.91	1.66	3.66	0.55	0.62
dcuC	moaABCDE	3.04	1.94	1.7	3.78	0.56	1.95
dcuC	moaABCDE	3.04	1.94	1.7	3.78	0.56	1.95
dcuC	moaABCDE	3.04	1.94	1.7	3.78	0.56	1.95
aer	ydhYVWXUT	1.64	2.21	0.93	0.31	0.57	0.14
dcuB_fumB	hypBCDE	2.95	3.35	1.72	5.28	0.58	1.58
dcuB_fumB	hypBCDE	2.95	3.35	1.72	5.28	0.58	1.58
dcuC	hypBCDE	2.95	3.35	1.77	4.08	0.60	1.22
dcuC	hypBCDE	2.95	3.35	1.77	4.08	0.60	1.22
dcuC	hypBCDE	2.95	3.35	1.77	4.08	0.60	1.22
dcuC	hypBCDE	2.95	3.35	1.77	4.08	0.60	1.22
dcuC	hypBCDE	2.95	3.35	1.77	4.08	0.60	1.22
dcuC	hypBCDE	2.95	3.35	1.77	4.08	0.60	1.22
tdcABCDEFG	dcuC	1.31	2.25	0.79	1.08	0.60	0.48
tdcABCDEFG	dcuC	1.31	2.25	0.79	1.08	0.60	0.48
tdcABCDEFG	dcuC	1.31	2.25	0.79	1.08	0.60	0.48
tdcABCDEFG	dcuC	1.31	2.25	0.79	1.08	0.60	0.48

tdcABCDEFG	dcuC	1.31	2.25	0.79	1.08	0.60	0.48
tdcABCDEFG	dcuC	1.31	2.25	0.79	1.08	0.60	0.48
aer	dcuB_fumB	1.5	4	0.93	0.28	0.62	0.07
caiTABCDE	ydhYVWXUT	1.64	2.21	1.04	0.94	0.63	0.43
caiTABCDE	ydhYVWXUT	1.64	2.21	1.04	0.94	0.63	0.43
aer	focA_pflB	1.46	3.62	0.93	0.3	0.64	0.08
aer	focA_pflB	1.46	3.62	0.93	0.3	0.64	0.08
ydhYVWXUT	yhjA	3	5.91	1.94	2.78	0.65	0.47
ydhYVWXUT	moaABCDE	3.04	1.94	2.03	2.94	0.67	1.52
dcuB_fumB	ydhYVWXUT	1.64	2.21	1.11	1.69	0.68	0.76
tdcABCDEFG	caiTABCDE	1.04	0.94	0.72	1.11	0.69	1.18
tdcABCDEFG	caiTABCDE	1.04	0.94	0.72	1.11	0.69	1.18
tdcABCDEFG	caiTABCDE	1.04	0.94	0.72	1.11	0.69	1.18
tdcABCDEFG	caiTABCDE	1.04	0.94	0.72	1.11	0.69	1.18
caiTABCDE	dcuB_fumB	1.5	4	1.04	0.94	0.69	0.24
caiTABCDE	dcuB_fumB	1.5	4	1.04	0.94	0.69	0.24
aer	nirBDC_cysG	1.34	1.08	0.93	0.28	0.69	0.26
aer	dcuC	1.31	2.25	0.93	0.36	0.71	0.16
aer	dcuC	1.31	2.25	0.93	0.36	0.71	0.16
aer	dcuC	1.31	2.25	0.93	0.36	0.71	0.16
caiTABCDE	focA_pflB	1.46	3.62	1.04	0.94	0.71	0.26
caiTABCDE	focA_pflB	1.46	3.62	1.04	0.94	0.71	0.26
caiTABCDE	focA_pflB	1.46	3.62	1.04	0.94	0.71	0.26
caiTABCDE	focA_pflB	1.46	3.62	1.04	0.94	0.71	0.26
ydhYVWXUT	hypBCDE	2.95	3.35	2.26	3.38	0.77	1.01
ydhYVWXUT	hypBCDE	2.95	3.35	2.26	3.38	0.77	1.01
caiTABCDE	nirBDC_cysG	1.34	1.08	1.04	0.94	0.78	0.87
caiTABCDE	nirBDC_cysG	1.34	1.08	1.04	0.94	0.78	0.87
caiTABCDE	dcuC	1.31	2.25	1.03	0.95	0.79	0.42
caiTABCDE	dcuC	1.31	2.25	1.03	0.95	0.79	0.42
caiTABCDE	dcuC	1.31	2.25	1.03	0.95	0.79	0.42
caiTABCDE	dcuC	1.31	2.25	1.03	0.95	0.79	0.42
caiTABCDE	dcuC	1.31	2.25	1.03	0.95	0.79	0.42
caiTABCDE	dcuC	1.31	2.25	1.03	0.95	0.79	0.42
dcuB_fumB	focA_pflB	1.46	3.62	1.15	1.91	0.79	0.53
dcuB_fumB	focA_pflB	1.46	3.62	1.15	1.91	0.79	0.53
dcuB_fumB	dcuC	1.31	2.25	1.04	1.24	0.79	0.55
dcuB_fumB	dcuC	1.31	2.25	1.04	1.24	0.79	0.55
dcuB_fumB	dcuC	1.31	2.25	1.04	1.24	0.79	0.55
nirBDC_cysG	ydhYVWXUT	1.64	2.21	1.31	1.07	0.80	0.48
aspA_dcuA	nikABCDER	0.21	0.46	0.17	1.38	0.81	3.00
tdcABCDEFG	aer	0.93	0.34	0.77	1.09	0.83	3.21
tdcABCDEFG	aer	0.93	0.34	0.77	1.09	0.83	3.21
focA_pflB	ydhYVWXUT	1.64	2.21	1.42	3.42	0.87	1.55
focA_pflB	ydhYVWXUT	1.64	2.21	1.42	3.42	0.87	1.55
nirBDC_cysG	dcuB_fumB	1.5	4	1.34	1.08	0.89	0.27
aer	caiTABCDE	1.04	0.94	0.93	0.29	0.89	0.31
aer	caiTABCDE	1.04	0.94	0.93	0.29	0.89	0.31
nirBDC_cysG	focA_pflB	1.46	3.62	1.32	1.08	0.90	0.30
nirBDC_cysG	focA_pflB	1.46	3.62	1.32	1.08	0.90	0.30

hypBCDE	yhjA	3	5.91	2.73	3.08	0.91	0.52
hypBCDE	yhjA	3	5.91	2.73	3.08	0.91	0.52
hypBCDE	moaABCDE	3.04	1.94	2.79	3.16	0.92	1.63
hypBCDE	moaABCDE	3.04	1.94	2.79	3.16	0.92	1.63
dcuC	ydhYVWXUT	1.64	2.21	1.53	3.11	0.93	1.41
dcuC	ydhYVWXUT	1.64	2.21	1.53	3.11	0.93	1.41
dcuC	ydhYVWXUT	1.64	2.21	1.53	3.11	0.93	1.41
nirBDC_cysG	dcuC	1.31	2.25	1.26	1.06	0.96	0.47
nirBDC_cysG	dcuC	1.31	2.25	1.26	1.06	0.96	0.47
nirBDC_cysG	dcuC	1.31	2.25	1.26	1.06	0.96	0.47
focA_pflB	dcuC	1.31	2.25	1.27	2.52	0.97	1.12
focA_pflB	dcuC	1.31	2.25	1.27	2.52	0.97	1.12
focA_pflB	dcuC	1.31	2.25	1.27	2.52	0.97	1.12
focA_pflB	dcuC	1.31	2.25	1.27	2.52	0.97	1.12
focA_pflB	dcuC	1.31	2.25	1.27	2.52	0.97	1.12
focA_pflB	dcuC	1.31	2.25	1.27	2.52	0.97	1.12
caiTABCDE	caiTABCDE	1.04	0.94	1.04	0.94	1.00	1.00
caiTABCDE	caiTABCDE	1.04	0.94	1.04	0.94	1.00	1.00
dcuC	dcuC	1.31	2.25	1.31	2.25	1.00	1.00
dcuC	dcuC	1.31	2.25	1.31	2.25	1.00	1.00
dcuC	dcuC	1.31	2.25	1.31	2.25	1.00	1.00
dcuC	dcuC	1.31	2.25	1.31	2.25	1.00	1.00
dcuC	dcuC	1.31	2.25	1.31	2.25	1.00	1.00
dcuC	dcuC	1.31	2.25	1.31	2.25	1.00	1.00
focA_pflB	focA_pflB	1.46	3.62	1.46	3.62	1.00	1.00
focA_pflB	focA_pflB	1.46	3.62	1.46	3.62	1.00	1.00
hypBCDE	hypBCDE	2.95	3.35	2.95	3.35	1.00	1.00
hypBCDE	hypBCDE	2.95	3.35	2.95	3.35	1.00	1.00
tdcABCDEFG	tdcABCDEFG	0.8	1.08	0.8	1.08	1.00	1.00
tdcABCDEFG	tdcABCDEFG	0.8	1.08	0.8	1.08	1.00	1.00
ydhYVWXUT	dcuC	1.31	2.25	1.31	1.59	1.00	0.71
ydhYVWXUT	dcuC	1.31	2.25	1.31	1.59	1.00	0.71
ydhYVWXUT	dcuC	1.31	2.25	1.31	1.59	1.00	0.71
moaABCDE	yhjA	3	5.91	3.01	1.92	1.00	0.32
yhjA	moaABCDE	3.04	1.94	3.16	6.31	1.04	3.25
focA_pflB	dcuB_fumB	1.5	4	1.56	4.21	1.04	1.05
focA_pflB	dcuB_fumB	1.5	4	1.56	4.21	1.04	1.05
moaABCDE	hypBCDE	2.95	3.35	3.11	1.97	1.05	0.59
moaABCDE	hypBCDE	2.95	3.35	3.11	1.97	1.05	0.59
dcuC	focA_pflB	1.46	3.62	1.58	3.32	1.08	0.92
dcuC	focA_pflB	1.46	3.62	1.58	3.32	1.08	0.92
dcuC	focA_pflB	1.46	3.62	1.58	3.32	1.08	0.92
dcuC	focA_pflB	1.46	3.62	1.58	3.32	1.08	0.92
dcuC	focA_pflB	1.46	3.62	1.58	3.32	1.08	0.92
dcuC	focA_pflB	1.46	3.62	1.58	3.32	1.08	0.92
caiTABCDE	aer	0.93	0.34	1.03	0.95	1.11	2.79
caiTABCDE	aer	0.93	0.34	1.03	0.95	1.11	2.79
dcuB_fumB	aer	0.93	0.34	1.06	1.34	1.14	3.94
dcuC	dcuB_fumB	1.5	4	1.74	3.97	1.16	0.99
dcuC	dcuB_fumB	1.5	4	1.74	3.97	1.16	0.99

dcuC	dcuB_fumB	1.5	4	1.74	3.97	1.16	0.99
focA_pflB	nirBDC_cysG	1.34	1.08	1.57	4.27	1.17	3.95
focA_pflB	nirBDC_cysG	1.34	1.08	1.57	4.27	1.17	3.95
aer	tdcABCDEFG	0.8	1.08	0.94	0.37	1.18	0.34
aer	tdcABCDEFG	0.8	1.08	0.94	0.37	1.18	0.34
ydhYVWXUT	focA_pflB	1.46	3.62	1.75	2.42	1.20	0.67
ydhYVWXUT	focA_pflB	1.46	3.62	1.75	2.42	1.20	0.67
dcuB_fumB	nirBDC_cysG	1.34	1.08	1.61	4.63	1.20	4.29
yhjA	hypBCDE	2.95	3.35	3.59	7.36	1.22	2.20
yhjA	hypBCDE	2.95	3.35	3.59	7.36	1.22	2.20
nirBDC_cysG	caiTABCDE	1.04	0.94	1.33	1.08	1.28	1.15
nirBDC_cysG	caiTABCDE	1.04	0.94	1.33	1.08	1.28	1.15
caiTABCDE	tdcABCDEFG	0.8	1.08	1.03	0.95	1.29	0.88
caiTABCDE	tdcABCDEFG	0.8	1.08	1.03	0.95	1.29	0.88
caiTABCDE	tdcABCDEFG	0.8	1.08	1.03	0.95	1.29	0.88
caiTABCDE	tdcABCDEFG	0.8	1.08	1.03	0.95	1.29	0.88
dcuB_fumB	tdcABCDEFG	0.8	1.08	1.03	1.2	1.29	1.11
dcuB_fumB	tdcABCDEFG	0.8	1.08	1.03	1.2	1.29	1.11
dcuB_fumB	caiTABCDE	1.04	0.94	1.34	3.07	1.29	3.27
dcuB_fumB	caiTABCDE	1.04	0.94	1.34	3.07	1.29	3.27
yhjA	dcuC	1.31	2.25	1.7	2.72	1.30	1.21
yhjA	dcuC	1.31	2.25	1.7	2.72	1.30	1.21
yhjA	dcuC	1.31	2.25	1.7	2.72	1.30	1.21
dcuC	nirBDC_cysG	1.34	1.08	1.76	4.03	1.31	3.73
dcuC	nirBDC_cysG	1.34	1.08	1.76	4.03	1.31	3.73
dcuC	nirBDC_cysG	1.34	1.08	1.76	4.03	1.31	3.73
nikABCDER	aspA_dcuA	0.15	1.39	0.2	0.45	1.33	0.32
nirBDC_cysG	aer	0.93	0.34	1.28	1.07	1.38	3.15
focA_pflB	aer	0.93	0.34	1.32	2.83	1.42	8.32
focA_pflB	aer	0.93	0.34	1.32	2.83	1.42	8.32
ydhYVWXUT	dcuB_fumB	1.5	4	2.17	3.21	1.45	0.80
hypBCDE	dcuC	1.31	2.25	1.9	2.08	1.45	0.92
hypBCDE	dcuC	1.31	2.25	1.9	2.08	1.45	0.92
hypBCDE	dcuC	1.31	2.25	1.9	2.08	1.45	0.92
hypBCDE	dcuC	1.31	2.25	1.9	2.08	1.45	0.92
hypBCDE	dcuC	1.31	2.25	1.9	2.08	1.45	0.92
hypBCDE	dcuC	1.31	2.25	1.9	2.08	1.45	0.92
yhjA	ydhYVWXUT	1.64	2.21	2.4	4.45	1.46	2.01
hypBCDE	ydhYVWXUT	1.64	2.21	2.42	2.72	1.48	1.23
hypBCDE	ydhYVWXUT	1.64	2.21	2.42	2.72	1.48	1.23
focA_pflB	caiTABCDE	1.04	0.94	1.54	4.08	1.48	4.34
focA_pflB	caiTABCDE	1.04	0.94	1.54	4.08	1.48	4.34
focA_pflB	caiTABCDE	1.04	0.94	1.54	4.08	1.48	4.34
focA_pflB	caiTABCDE	1.04	0.94	1.54	4.08	1.48	4.34
dcuC	aer	0.93	0.34	1.38	2.53	1.48	7.44
dcuC	aer	0.93	0.34	1.38	2.53	1.48	7.44
dcuC	aer	0.93	0.34	1.38	2.53	1.48	7.44
ydhYVWXUT	aer	0.93	0.34	1.4	1.76	1.51	5.18
focA_pflB	tdcABCDEFG	0.8	1.08	1.24	2.36	1.55	2.19
focA_pflB	tdcABCDEFG	0.8	1.08	1.24	2.36	1.55	2.19

focA_pflB	tdcABCDEFG	0.8	1.08	1.24	2.36	1.55	2.19
focA_pflB	tdcABCDEFG	0.8	1.08	1.24	2.36	1.55	2.19
nirBDC_cysG	tdcABCDEFG	0.8	1.08	1.24	1.06	1.55	0.98
nirBDC_cysG	tdcABCDEFG	0.8	1.08	1.24	1.06	1.55	0.98
ydhYVWXUT	tdcABCDEFG	0.8	1.08	1.27	1.5	1.59	1.39
ydhYVWXUT	tdcABCDEFG	0.8	1.08	1.27	1.5	1.59	1.39
dcuC	tdcABCDEFG	0.8	1.08	1.28	2.11	1.60	1.95
dcuC	tdcABCDEFG	0.8	1.08	1.28	2.11	1.60	1.95
dcuC	tdcABCDEFG	0.8	1.08	1.28	2.11	1.60	1.95
dcuC	tdcABCDEFG	0.8	1.08	1.28	2.11	1.60	1.95
dcuC	tdcABCDEFG	0.8	1.08	1.28	2.11	1.60	1.95
dcuC	tdcABCDEFG	0.8	1.08	1.28	2.11	1.60	1.95
dcuC	caiTABCDE	1.04	0.94	1.71	3.82	1.64	4.06
dcuC	caiTABCDE	1.04	0.94	1.71	3.82	1.64	4.06
dcuC	caiTABCDE	1.04	0.94	1.71	3.82	1.64	4.06
dcuC	caiTABCDE	1.04	0.94	1.71	3.82	1.64	4.06
dcuC	caiTABCDE	1.04	0.94	1.71	3.82	1.64	4.06
dcuC	caiTABCDE	1.04	0.94	1.71	3.82	1.64	4.06
ydhYVWXUT	nirBDC_cysG	1.34	1.08	2.22	3.31	1.66	3.06
moaABCDE	ydhYVWXUT	1.64	2.21	2.84	1.85	1.73	0.84
hypBCDE	focA_pflB	1.46	3.62	2.55	2.86	1.75	0.79
hypBCDE	focA_pflB	1.46	3.62	2.55	2.86	1.75	0.79
hypBCDE	focA_pflB	1.46	3.62	2.55	2.86	1.75	0.79
hypBCDE	focA_pflB	1.46	3.62	2.55	2.86	1.75	0.79
yhjA	focA_pflB	1.46	3.62	2.62	4.98	1.79	1.38
yhjA	focA_pflB	1.46	3.62	2.62	4.98	1.79	1.38
moaABCDE	dcuC	1.31	2.25	2.43	1.66	1.85	0.74
moaABCDE	dcuC	1.31	2.25	2.43	1.66	1.85	0.74
moaABCDE	dcuC	1.31	2.25	2.43	1.66	1.85	0.74
hypBCDE	dcuB_fumB	1.5	4	2.89	3.28	1.93	0.82
hypBCDE	dcuB_fumB	1.5	4	2.89	3.28	1.93	0.82
ydhYVWXUT	caiTABCDE	1.04	0.94	2.05	2.99	1.97	3.18
ydhYVWXUT	caiTABCDE	1.04	0.94	2.05	2.99	1.97	3.18
moaABCDE	focA_pflB	1.46	3.62	2.91	1.88	1.99	0.52
moaABCDE	focA_pflB	1.46	3.62	2.91	1.88	1.99	0.52
yhjA	tdcABCDEFG	0.8	1.08	1.6	2.48	2.00	2.30
yhjA	tdcABCDEFG	0.8	1.08	1.6	2.48	2.00	2.30
yhjA	aer	0.93	0.34	1.9	3.22	2.04	9.47
moaABCDE	dcuB_fumB	1.5	4	3.09	1.96	2.06	0.49
hypBCDE	nirBDC_cysG	1.34	1.08	2.93	3.32	2.19	3.07
hypBCDE	nirBDC_cysG	1.34	1.08	2.93	3.32	2.19	3.07
hypBCDE	aer	0.93	0.34	2.08	2.3	2.24	6.76
hypBCDE	aer	0.93	0.34	2.08	2.3	2.24	6.76
hypBCDE	tdcABCDEFG	0.8	1.08	1.8	1.97	2.25	1.82
hypBCDE	tdcABCDEFG	0.8	1.08	1.8	1.97	2.25	1.82
hypBCDE	tdcABCDEFG	0.8	1.08	1.8	1.97	2.25	1.82
hypBCDE	tdcABCDEFG	0.8	1.08	1.8	1.97	2.25	1.82
yhjA	dcuB_fumB	1.5	4	3.42	6.95	2.28	1.74
moaABCDE	nirBDC_cysG	1.34	1.08	3.1	1.97	2.31	1.82
yhjA	nirBDC_cysG	1.34	1.08	3.52	7.18	2.63	6.65

hypBCDE	caiTABCDE	1.04	0.94	2.81	3.19	2.70	3.39
hypBCDE	caiTABCDE	1.04	0.94	2.81	3.19	2.70	3.39
hypBCDE	caiTABCDE	1.04	0.94	2.81	3.19	2.70	3.39
hypBCDE	caiTABCDE	1.04	0.94	2.81	3.19	2.70	3.39
moaABCDE	aer	0.93	0.34	2.59	1.73	2.78	5.09
moaABCDE	tdcABCDEFG	0.8	1.08	2.33	1.61	2.91	1.49
moaABCDE	tdcABCDEFG	0.8	1.08	2.33	1.61	2.91	1.49
moaABCDE	caiTABCDE	1.04	0.94	3.05	1.94	2.93	2.06
moaABCDE	caiTABCDE	1.04	0.94	3.05	1.94	2.93	2.06
yhjA	caiTABCDE	1.04	0.94	3.21	6.43	3.09	6.84
yhjA	caiTABCDE	1.04	0.94	3.21	6.43	3.09	6.84
tdcABCDEFG	nikABCDER	0.21	0.46	0.73	1.11	3.48	2.41
tdcABCDEFG	nikABCDER	0.21	0.46	0.73	1.11	3.48	2.41
aer	nikABCDER	0.21	0.46	0.93	0.29	4.43	0.63
tdcABCDEFG	aspA_dcuA	0.15	1.39	0.72	1.11	4.80	0.80
tdcABCDEFG	aspA_dcuA	0.15	1.39	0.72	1.11	4.80	0.80
caiTABCDE	nikABCDER	0.21	0.46	1.04	0.94	4.95	2.04
caiTABCDE	nikABCDER	0.21	0.46	1.04	0.94	4.95	2.04
dcuB_fumB	nikABCDER	0.21	0.46	1.21	2.23	5.76	4.85
aer	aspA_dcuA	0.15	1.39	0.93	0.29	6.20	0.21
nirBDC_cysG	nikABCDER	0.21	0.46	1.32	1.08	6.29	2.35
caiTABCDE	aspA_dcuA	0.15	1.39	1.04	0.94	6.93	0.68
caiTABCDE	aspA_dcuA	0.15	1.39	1.04	0.94	6.93	0.68
focA_pflB	nikABCDER	0.21	0.46	1.49	3.82	7.10	8.30
focA_pflB	nikABCDER	0.21	0.46	1.49	3.82	7.10	8.30
dcuC	nikABCDER	0.21	0.46	1.63	3.53	7.76	7.67
dcuC	nikABCDER	0.21	0.46	1.63	3.53	7.76	7.67
dcuC	nikABCDER	0.21	0.46	1.63	3.53	7.76	7.67
dcuB_fumB	aspA_dcuA	0.15	1.39	1.27	2.62	8.47	1.88
nirBDC_cysG	aspA_dcuA	0.15	1.39	1.33	1.08	8.87	0.78
ydhYVWXUT	nikABCDER	0.21	0.46	1.87	2.64	8.90	5.74
focA_pflB	aspA_dcuA	0.15	1.39	1.52	3.97	10.13	2.86
focA_pflB	aspA_dcuA	0.15	1.39	1.52	3.97	10.13	2.86
dcuC	aspA_dcuA	0.15	1.39	1.67	3.69	11.13	2.65
dcuC	aspA_dcuA	0.15	1.39	1.67	3.69	11.13	2.65
dcuC	aspA_dcuA	0.15	1.39	1.67	3.69	11.13	2.65
hypBCDE	nikABCDER	0.21	0.46	2.66	3	12.67	6.52
hypBCDE	nikABCDER	0.21	0.46	2.66	3	12.67	6.52
ydhYVWXUT	aspA_dcuA	0.15	1.39	1.97	2.83	13.13	2.04
yhjA	nikABCDER	0.21	0.46	2.85	5.54	13.57	12.04
moaABCDE	nikABCDER	0.21	0.46	2.97	1.91	14.14	4.15
hypBCDE	aspA_dcuA	0.15	1.39	2.75	3.11	18.33	2.24
hypBCDE	aspA_dcuA	0.15	1.39	2.75	3.11	18.33	2.24
moaABCDE	aspA_dcuA	0.15	1.39	3.02	1.93	20.13	1.39
yhjA	aspA_dcuA	0.15	1.39	3.05	6.02	20.33	4.33
