# Supplementary material for: From sequence to dynamics: the effects of transcription factor and polymerase concentration changes on activated and repressed promoters
Source: BMC Mol Biol. 2009 Sep 22;10:92. doi: 10.1186/1471-2199-10-92 (PMC2761915; doi:10.1186/1471-2199-10-92)

Additional file 4: Polymerase—*glmUS* promoter interaction probability as a function of NagC concentration. Four different ω values (10, 20, 50, 100) were used in these simulations. ([*Pol*] = 5E—09.)

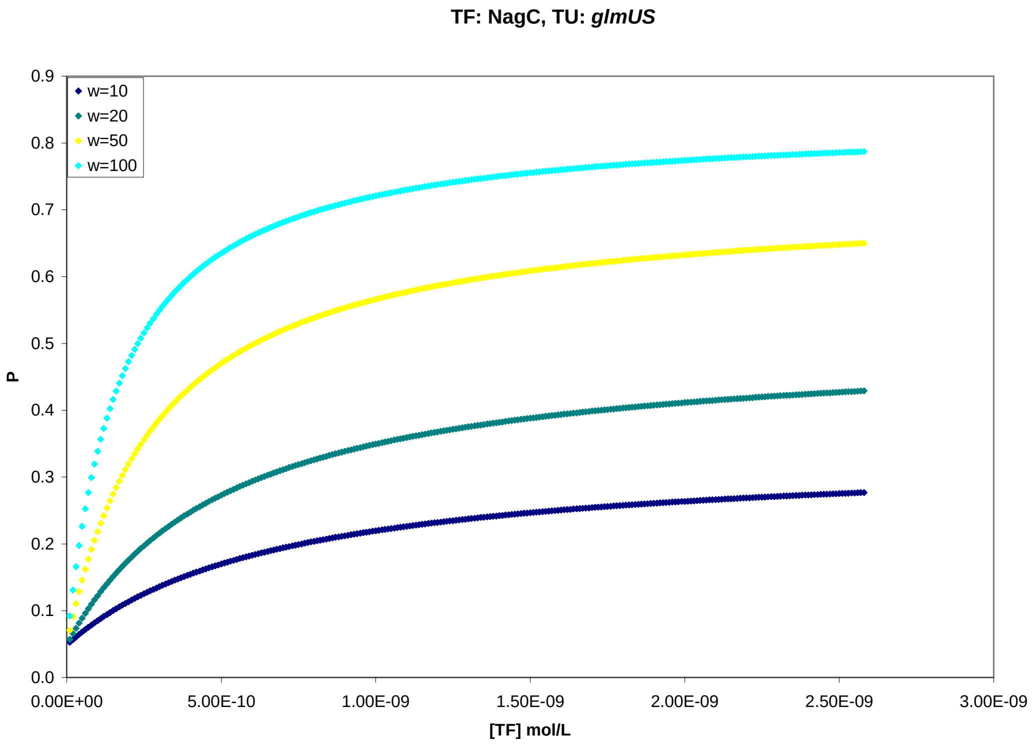

Supplement: Additional file 4 — Polymerase-promoter probability vs TF concentration calculated using four different ω values. This fail can be open with PDF viewer. [file 1471-2199-10-92-S4.pdf]
